# Click Chemistry Functionalization of Harmonic Nanoparticles with Lanthanide Complexes Towards Tunable Platforms for Multimodal Imaging

**DOI:** 10.3390/nano16100591

**Published:** 2026-05-12

**Authors:** Simon Dumolard, Volodymyr Multian, Adrian Gheata, Alessandra Spada, Katarzyna Pierzchala, Bernard Lanz, Ameni Dhouib, Yannick Mugnier, Jérémie Teyssier, Luigi Bonacina, Anne-Sophie Chauvin, Sandrine Gerber-Lemaire

**Affiliations:** 1 Group for Functionalized Biomaterials, Institute of Chemical Sciences and Engineering, Ecole Polytechnique Fédérale de Lausanne (EPFL), CH-1015 Lausanne, Switzerland; simon.dumolard@epfl.ch (S.D.); adrian.gheata@gmail.com (A.G.); alessandra.spada@epfl.ch (A.S.); 2 Nonlinear BioImaging LAB, Department of Applied Physics, Université de Genève, CH-1205 Genève, Switzerland; volodymyr.multian@unige.ch (V.M.); luigi.bonacina@unige.ch (L.B.); 3 Department of Quantum Matter Physics, Université de Genève, CH-1211 Genève, Switzerland; jeremie.teyssier@unige.ch; 4 CIBM Center for Biomedical Imaging, Ecole Polytechnique Fédérale de Lausanne (EPFL), CH-1015 Lausanne, Switzerland; katarzyna.pierzchala@epfl.ch (K.P.); bernard.lanz@epfl.ch (B.L.); 5 CIBM Pre-Clinical Imaging EPFL Metabolic Imaging Section, École Polytechnique Fédérale de Lausanne (EPFL), CH-1015 Lausanne, Switzerland; 6 SYMME, Université Savoie Mont-Blanc, F-74000 Annecy, Franceyannick.mugnier@univ-smb.fr (Y.M.); 7 Laboratory of Supramolecular Chemistry, Institute of Chemical Sciences and Engineering, Ecole Polytechnique Fédérale de Lausanne (EPFL), CH-1015 Lausanne, Switzerland

**Keywords:** click chemistry, lanthanides photoluminescence, magnetic resonance imaging, nonlinear optics, multimodal imaging

## Abstract

Nanoplatforms combining multiple imaging contrast modalities are gaining interest across life sciences and beyond. Here, we disclose a proof-of-concept series of harmonic nanoparticles (HNPs) conjugated with a variety of lanthanide (Ln) complexes, enabling tunable imaging properties. Building on our previous approach for the conjugation of Gd(III) complexes at the surface of HNPs through copper-catalyzed click chemistry, we first establish a copper-free alternative by benchmarking the signals of the resulting conjugates in magnetic resonance imaging phantoms. We then extend this system to Eu, Tb and Yb conjugates and investigate their photophysical properties, successfully detecting long-lived Ln emissions spanning the visible and near-infrared spectrum. Interestingly, the Ln ion can be efficiently removed and exchanged, allowing reuse of the same HNP with a new optical signature. Most notably, we demonstrate that the Eu luminescence can be indirectly activated via second-harmonic generation from the HNP core upon femtosecond-pulsed irradiation in parallel to direct two-photon excitation. This nonlinear activation scheme paves the way for the preparation of mixtures with multidimensional optical signatures using a single excitation source. Altogether this work provides a versatile framework to further explore HNP-Ln conjugates as multimodal imaging probes.

## 1. Introduction

Imaging plays a key role in life sciences to investigate biological processes and to provide tools enabling medical diagnostics [1]. While individual imaging techniques have reached a high level of performance, they remain intrinsically limited by parameters like sensitivity, resolution and penetration depth [2,3,4]. Multimodal imaging, based on the acquisition of different contrast mechanisms, has gained interest as it can complement limitations of single techniques and acquire multidimensional data for improved accuracy [4,5]. For example, Baranski et al. reported a prostate-targeted dual probe that allowed to conduct pre-operative positron emission tomography (PET)/computed tomography (CT) and assist tumor resection via fluorescent visualization [6,7]. The combination of magnetic resonance imaging (MRI) with optical signals also holds great potential for increased sensitivity and functional readout across multiple length scale [8,9,10]. It is worth mentioning that multimodal imaging is increasingly used beyond the biomedical field for applications like cultural heritage preservation [11], material manufacturing [12] or anti-counterfeiting [13,14].

One barrier to the widespread use of multimodal imaging is the requirement for contrast agents (CAs) able to perform across different modalities [15,16]. Compared to small-molecule-based probes, nanoparticles (NPs) can be engineered for multiple imaging techniques while maintaining stability via modulation of their composition and surface chemistry. Harmonic nanoparticles (HNPs) represent a particularly interesting substrate for CA development thanks to their well-established nonlinear optical properties and biocompatibility [17,18,19]. For example, their tunable second- and third-harmonic generation (SHG and THG, respectively) have been successfully applied to selective cancer cell imaging [20], and have demonstrated their potential to detect stem cells in tissue imaging [21,22] and in flowing blood samples [23].

Trivalent lanthanide (Ln) ions possess unique electronic configurations giving rise to narrow, long-lived f-f emission bands, making them excellent optical probes with high contrast abilities [24]. In addition to their photoluminescence (PL) properties, Ln can provide strong paramagnetism relevant to MRI (e.g., Gd) [25], high mass attenuation coefficients applicable to X-ray CT (e.g., Yb, Dy, Lu) [26], or stable isotope (e.g., ^177^Lu, ^153^Sm) for PET and single-photon emission computed tomography (SPECT) [27]. Ln ions have thus been incorporated into inorganic or hybrid NP designs [24]. This includes functionalized iron oxide NPs [28], mesoporous silica NPs [29] or core–shell mesoporous silica–gadolinium oxide composite NPs [30], as well as core–shell [31] and core–multi-shell [32] upconversion NPs. The optical signal can further be leveraged for multicolor barcoding [33,34] or for in situ sensing of tumor microenvironment markers [35], pH [36] and temperature [37,38]. Ln doping can however lead to undesired luminescence quenching [39] or loss of nonlinear optical response in harmonic nanocrystals [40,41], which requires careful control over synthetic conditions. Surface functionalization with Ln complexes offers an interesting alternative by maintaining crystal structure integrity thanks to robust conjugation chemistries [42]. Design optimization, e.g., efficient Ln sensitization via the ligand (antenna effect) or enhanced emission lifetimes, can therefore be performed separately with high modularity [43]. Moreover, this approach allows to fine-tune the contact with the surrounding environment, which is required to generate a stable contrast in MRI and for sensing applications [44,45]. Though a few examples of such covalent strategies can be found with iron oxide NPs [46,47], gold NPs [48], silica NPs [49] and polymeric NPs [50], the investigation of nanomaterials post-modification with Ln complexes remains elusive.

**Figure 1:**
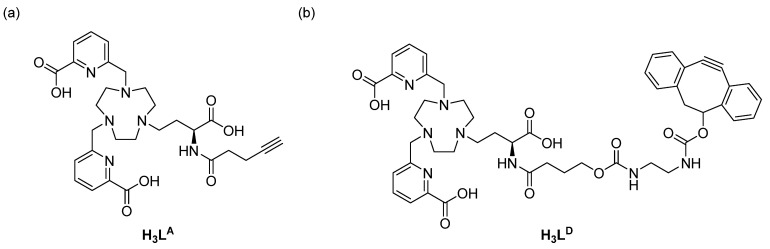
Chemical structures of (**a**) the terminal alkyne-bearing **H_3_L^A^** ligand and (**b**) its DIBO-bearing **H_3_L^D^** counterpart.

Tripodal 1,4,7-triazacyclononane (TACN) ligands have been widely investigated as efficient and versatile Ln binders, resulting in efficient constructs for PL and MRI applications [51,52,53,54]. In our previous study, an analog to one such ligand allowing fast water exchange (**H_3_ebpatcn**) [55] was derivatized with a terminal alkyne (Figure 1a, **A**lkyne ligand named **H_3_L^A^**), complexed with Gd(III) and conjugated to the surface of LiNbO_3_ (LNO) HNPs via Cu-catalyzed [3+2] azide–alkyne cycloaddition (CuAAC) [56]. The resulting suspension was successfully applied to bimodal nonlinear microscopy and MRI with both *T*_1_ and *T*_2_ contrast capabilities. While highly efficient and well-studied, CuAAC can be associated with a number of challenges during conjugation including possible azide side reactions [57] and competitive Cu(I) or Cu(II) complexation against Ln [58,59]. In addition, the presence of Cu residues can generate reactive oxygen species (ROS) that can degrade the ligand and cause cytotoxicity when applied in biological systems [60,61,62]. Conversely, Cu-free click chemistries such as strain-promoted [3+2] azide–alkyne cycloaddition (SPAAC) and inverse-electron-demand Diels–Alder (IEDDA) cycloaddition allow for milder reaction conditions, but require the use of bulkier, synthetically more-demanding substrates [42,63]. In the present work, an analogous ligand containing a strained dibenzocyclooctyne (DIBO) residue was designed (Figure 1b, **D**IBO ligand named **H_3_L^D^**) to enable SPAAC conjugation, thereby avoiding the use of a copper catalyst. This new ligand was first complexed with Gd(III) ions and benchmarked against the established dual MRI–nonlinear microscopy capacity of the previous platform. Moreover, both **H_3_L^A^** and **H_3_L^D^** ligands were reacted with Gd, Eu, Yb and Tb ions and the resulting complexes were assessed for their PL properties, before and after conjugation to LNO HNPs. Proof-of-concept nanoplatform recycling with Tb, and the combination of Eu and SHG signals demonstrated the potential of LNO@[Ln] conjugates for versatile multidimensional imaging.

## 2. Materials and Methods

### 2.1. Synthesis Procedures and Characterizations

The **TACNB** [56] and **DIBO-NH_2_** [64,65] intermediates were synthesized according to our reported procedures. The ^1^H-NMR and mass spectra were in accordance with the reported data.

#### 2.1.1. Compound **1**

The synthesis of compound 1 was adapted from Li et al. [66]. To a round-bottom flask, γ-butyrolactone (2.02 mL, 26 mmol, 1.0 eq.), PMBBr (9.5 mL, 66 mmol, 2.5 eq.) and KOH (6.8 mg, 106 mmol, 4.0 eq.) were dissolved under Ar in dry toluene (100 mL). The reaction was stirred under reflux for 4 days. The reaction was cooled to r.t. and diluted with AcOEt (250 mL) and washed with H_2_O (3×). The combined organic layers were dried over MgSO_4_, filtered and concentrated under reduced pressure to retrieve the product without further purification as a yellow oil (5.6 g, 25 mmol, 95%).

**MS** (ESI-Quad) m/z: [M − H]^−^ calcd for C_12_H_16_O_4_^−^ 223.0976; found 223.0975.

**^1^H-NMR** (400 MHz, Chloroform-*d*) δ 7.25 (d, 3H), 6.88 (d, 2H), 4.44 (s, 2H), 3.80 (s, 3H), 3.50 (t, *J* = 6.1 Hz, 2H), 2.47 (t, *J* = 7.3 Hz, 2H), 1.93 (p, *J* = 6.1 Hz, 2H).

The data were in accordance with the literature.

#### 2.1.2. Compound **2**

To a round-bottom flask under Ar, **1** (3.227 g, 14.39 mmol, 1.0 eq.) and NHS (1.662 g, 14.44 mmol, 1.0 eq.) were dissolved in dry THF (19.2 mL), and then cooled to 0 °C. To a separate flask under Ar, DCC (2.969 g, 14.39 mmol, 1.0 eq.) was dissolved in dry THF (9.6 mL), and the solution was then added dropwise into the first solution. The reaction was stirred at 0 °C for 1 h, and then at r.t. for 3 h. The reaction was filtered and concentrated under reduced pressure. The residue was redissolved in AcOEt (100 mL), filtered, and the filtrate was washed with NaHCO_3_ sat. (100 mL) and brine (100 mL). The organic layer was dried over MgSO_4_, filtered and concentrated under reduced pressure to retrieve the crude product as a yellow oil (4.235 g, 13.18 mmol, 92%).

To a round-bottom flask under Ar, the previous crude (1.652 g, 5.14 mmol, 1.0 eq.) and (S)-2-amino-4-butyrolactone hydrobromide (1.028 g, 5.65 mmol, 1.1 eq.) were dissolved in dry DCM (50 mL). To a separate flask under Ar, TEA (2.15 mL, 15.43 mmol, 3.0 eq.) was dissolved in dry DCM (2.6 mL), and the solution was then added dropwise into the first solution. The reaction was stirred at r.t. for 2 h. The reaction was then washed with NH_4_Cl sat. (50 mL) and extracted with DCM (50 mL). The combined organic layers were dried over MgSO_4_, filtered and concentrated under reduced pressure. The crude was purified by FC eluting with a gradient of EtOAc/MeOH (1:0 then 25:1) to retrieve the product as a white powder (1.065 g, 3.46 mmol, 67%).

**MS** (ESI-Quad) m/z: [M + H]^+^ calcd for C_16_H_22_NO_5_^+^ 308.1492; found 307.8016, and [M + Na]^+^ calcd for C_16_H_21_NNaO_5_^+^ 330.1312; found 330.1271.

**^1^H-NMR** (400 MHz, Chloroform-*d*) δ 7.28–7.23 (m, 2H, Ar-*H*), 6.91–6.86 (m, 2H, Ar-*H*), 6.40 (s, 1H, N*H*), 4.60–4.15 (m, 5H, C*H-CH2-C*H2* and O-C*H2*-Ar and C*H), 3.80 (s, 3H, O-C*H3*), 3.51 (t, *J* = 5.8 Hz, 2H, CH2-C*H2*-O), 2.76–2.66 (m, 1H, C*H-C*H2*-CH2), 2.38 (t, *J* = 7.0 Hz, 2H, CO-C*H2*-CH2), 1.98–1.90 (m, 2H. CH2-C*H2*-CH2), 1.90–1.83 (m, 1H, C*H-C*H2*-CH2).

**^13^C-NMR** (101 MHz, Chloroform-d): δ 175.30 (*C*OO-C*H), 173.45 (*C*ONH), 159.30 (*C*-OCH3), 130.25 (O-CH2-*C*), 129.57 (2× *C*H-C-OCH3), 113.87 (2× O-CH2-C-*C*H), 72.71 (O-*C*H2-C), 69.00 (CH2-*C*H2-O), 65.92 (C*H-CH2-*C*H2), 55.30 (O-*C*H3), 49.04 (COO-C*H), 33.41 (*C*H2-CH2-CH2), 30.25 (C*H-*C*H2-CH2), 25.40 (CH2-*C*H2-CH2).

#### 2.1.3. Compound **3**

To a round-bottom flask, **2** (503.3 mg, 1.64 mmol, 1.0 eq.) and H_2_SO_4_ (100 μL) were dissolved in EtOH (10 mL). The reaction was stirred at r.t. for 24 h. The reaction was then neutralized in an ice bath with NaHCO_3_ (s), and EtOH was added to maintain stirring. The mixture was then filtered and concentrated under reduced pressure at 20 °C. The crude was not purified and was directly used in the next step.

To a round-bottom flask under Ar, (COCl)_2_ (0.56 mL, 6.55 mmol, 4.0 eq.) was dissolved in dry DCM (10 mL). To a separate round-bottom flask under Ar, DMSO (0.70 mL, 9.82 mmol, 6.0 eq.) was dissolved in dry DCM (1.30 mL), and then added dropwise to the (COCl)_2_ solution. The mixture was stirred at −78 °C for 10 min. To a separate round-bottom flask under Ar, the crude alcohol intermediate was dissolved in dry DCM (5 mL). The solution was then added dropwise into the reaction mixture and stirred at −78 °C for 30 min. TEA (1.83 mL, 13.10 mmol, 8.0 eq.) was then added dropwise, the reaction was allowed to warm up to 0 °C and was stirred for 2 h. The reaction was further allowed to warm up to r.t. for 1 h. The reaction was quenched with H_2_O (10 mL) and extracted with DCM (3 × 10 mL). The combined organic layers were dried over MgSO_4_, filtered and concentrated under reduced pressure. The crude was purified by FC eluting with PE/EtOAc (1:3) to retrieve the product as a yellow oil (253.5 mg, 0.72 mmol, 44%).

**MS** (ESI-Quad) m/z: [M + H]^+^ calcd for C_18_H_26_NO_6_^+^ 352.1755; found 351.9747, and [M + Na]^+^ calcd for C_18_H_25_NNaO_6_^+^ 374.1574; found 374.2094.

**^1^H-NMR** (400 MHz, Chloroform-*d*) δ 9.67 (s, 1H, C*H*O), 7.29–7.22 (m, 2H, Ar-*H*), 6.91–6.84 (m, 2H, Ar-*H*), 6.56 (d, *J* = 7.7 Hz, 1H, N*H*), 4.85–4.78 (m, 1H, C*-*H*), 4.42 (s, 2H, O-C*H2*-Ar), 4.20 (q, *J* = 7.1 Hz, 2H, O-C*H2*-CH3), 3.80 (s, 3H, O-C*H3*), 3.47 (td, *J* = 6.1, 2.3 Hz, 2H, CH2-C*H2*-O), 3.10–2.94 (m, 2H, CHO-C*H2*-C*H), 2.33 (td, *J* = 7.2, 3.0 Hz, 2H, CO-C*H2*-CH2), 1.91 (q, 2H, CH2-C*H2*-CH2), 1.25 (t, *J* = 7.1 Hz, 3H, O-CH2-C*H3*).

**^13^C-NMR** (101 MHz, Chloroform-d): δ 199.29 (*C*HO), 172.64 (*C*ONH), 170.67 (*C*OO), 159.22 (*C*-OCH3), 130.41 (O-CH2-*C*), 129.38 (2× *C*H-C-OCH3), 113.81 (2× O-CH2-C-*C*H), 72.61 (O-*C*H2-C), 68.92 (CH2-*C*H2-O), 61.97 (O-*C*H2-CH3), 55.29 (O-*C*H3), 47.32 (COO-C*H), 45.69 (C*H-*C*H2-CHO), 33.29 (*C*H2-CH2-CH2), 25.50 (CH2-*C*H2-CH2), 14.06 (O-CH2-*C*H3).

#### 2.1.4. Compound **4**

To a round-bottom flask under Ar, **TACNB** (153.0 mg, 0.34 mmol, 1.1 eq.) was dissolved in dry DCE (5 mL). To a separate round-bottom flask under Ar, **3** (107.3 mg, 0.31 mmol, 1.0 eq.) and AcOH (17.3 µL, 0.30 mmol, 1.0 eq.) were dissolved in dry DCE (2.5 mL), and the solution was then added dropwise into the first solution. The reaction was stirred at r.t. for 5 h. NaBH(OAc)_3_ (320.9 mg, 1.51 mmol, 5.0 eq.) was then added, the flask was rinsed with dry DCE (1 mL), and the mixture was briefly ultrasonicated. The reaction was then stirred at r.t. for an additional 16 h. The reaction was quenched with NaHCO_3_ sat. (10 mL) and extracted with CHCl_3_ (4 × 5 mL). The organic layers were washed with brine (10 mL), dried over Na_2_SO_4_, filtered and concentrated under reduced pressure. The crude was purified by FC (Al_2_O_3_ basic III, 4.9% H_2_O) eluting with a DCM/MeOH gradient (1:0 to 30:1) to retrieve the product as a yellow oil (133.6 mg, 0.17 mmol, 55%).

**HRMS** (ESI/QTOF) m/z: [M + H]^+^ Calcd for C_42_H_59_N_6_O_9_^+^ 791.4338; found 791.4356.

**^1^H-NMR** (400 MHz, Acetonitrile-*d*_3_) δ 7.95–7.90 (m, 2H, Py-*H*), 7.88–7.81 (m, 2H, Py-*H*), 7.76–7.70 (m, 2H, Py-*H*), 7.25–7.18 (m, 3H, Ar-*H*), 6.89–6.83 (m, 2H, Ar-*H*), 4.48–4.41 (m, 1H, C*-*H*), 4.41–4.31 (m, 6H, O-C*H2*-CH3), 4.09 (m, 2H, CH2-C*H2*-C*H), 3.89 (s, 2H, O-C*H2*-Ar), 3.83 (s, 4H, Py-C*H2*-N), 3.75 (s, 3H, O-C*H3*), 3.42 (t, *J* = 6.3 Hz, 2H, CH2-C*H2*-O), 2.92–2.65 (m, 12H, N-C*H2*-C*H2*-N), 2.55–2.47 (br, 2H, N-C*H2*-CH2-C*), 2.22 (t, *J* = 7.3 Hz, 2H, CO-C*H2*-CH2), 1.84–1.76 (m, 2H, CH2-C*H2*-CH2), 1.35 (t, *J* = 7.1 Hz, 6H, O-CH2-C*H3*), 1.19 (t, *J* = 7.1 Hz, 3H, O-CH2-C*H3*).

**^13^C-NMR** (101 MHz, CD_3_CN) δ 173.47 (*C*ONH), 166.73 (*C*OO), 166.23 (*C*OO), 160.12 (*C*-O-CH3), 148.53 (*C*-Py), 138.36 (*C*-Py), 131.94 (O-CH2-*C*), 130.20 (2× *C*H-C-OCH3), 127.59 (*C*-Py), 127.46 (*C*-Py), 124.14 (*C*-Py), 114.58 (2× O-CH2-C-*C*H), 72.98 (O-*C*H2-CH3), 70.01 (CH2-*C*H2-O), 62.29 (O-*C*H2-CH3), 61.68 (CH2-*C*H2-C*H), 55.85 (O-*C*H3), 55.33 (N-*C*H2-CH2-C*H), 53.01 (O-*C*H2-Ar), 33.29 (CO-*C*H2-CH2), 26.55 (CH2-*C*H2-CH2), 14.59 (O-CH2-*C*H3), 14.55 (O-CH2-*C*H3).

#### 2.1.5. Compound **5**

To a round-bottom flask, **4** was dissolved in DCM (4 mL). PBS (1 mL) was added and the mixture was cooled to 0 °C. DDQ (109.3 mg, 0.48 mmol, 3.0 eq.) was then added and the reaction was stirred at 0 °C for 1 h. The reaction was then allowed to warm up to r.t. and stirred for an additional 3 h. The reaction was briefly ultrasonicated every 1 h. A second portion of DDQ (19.1 mg, 0.08 mmol, 0.5 eq.) was added if complete conversion was not achieved, and the reaction was stirred at r.t. for an additional 30 min. The reaction was transferred to an extraction funnel. The reaction flask was washed and ultrasonicated with DCM (2×) and then transferred to the extraction funnel. The reaction was quenched directly in the reaction flask with Na_2_S_2_O_3_ sat. (2 × 7.5 mL) and NaHCO_3_ sat. (2 × 7.5 mL). The mixture was ultrasonicated for 2 min after every addition and transferred to the extraction funnel using a glass Pasteur pipette. The reaction was then extracted with DCM (3 × 15 mL). The combined organic layers were washed with NaHCO_3_ sat. (15 mL) and brine (15 mL), dried over Na_2_SO_4_, filtered and concentrated under reduced pressure. The crude was purified by FC (Al_2_O_3_ basic III, 4.9% H_2_O) eluting with a gradient of DCM/MeOH (1:0 to 20:1) to retrieve the product as a brown oil (50.7 mg, 0.08 mmol, 47%).

**HRMS** (nanochip-ESI/LTQ-Orbitrap) m/z: [M + H]^+^ Calcd for C_34_H_51_N_6_O_8_^+^ 671.3763; found 671.3746.

**^1^H-NMR** (400 MHz, Acetonitrile-*d*_3_) δ 7.97–7.91 (m, 2H, Py-*H*), 7.90–7.83 (m, 2H, Py-*H*), 7.79–7.67 (m, 2H, Py-*H*), 7.28–7.13 (br, 1H, N*H*), 4.50–4.42 (m, 1H, C*-*H*), 4.36 (q, *J* = 7.1 Hz, 4H, O-C*H*2-CH3), 4.16–4.03 (m, 2H, CH2-C*H2*-C*H), 3.88–3.83 (m, 4H, Py-C*H*2-N), 3.49 (t, *J* = 6.2 Hz, 2H, CH2-C*H2*-O), 2.97–2.68 (m, 12H, N-C*H2*-C*H2*-N), 2.69–2.43 (m, 2H, N-C*H*2-CH2-C*), 2.23 (t, *J* = 6.8 Hz, 2H, CO-C*H2*-CH2), 1.72 (p, *J* = 6.8 Hz, 2H, CH2-C*H*2-CH2), 1.41–1.31 (m, 6H, O-CH2-C*H*3), 1.19 (t, *J* = 7.1 Hz, 3H, O-CH2-C*H*3).

#### 2.1.6. Compound **6**

To a round-bottom flask under Ar, 4-nitrophenyl chloroformate (22.3 mg, 0.111 mmol, 2.0 eq.) was dissolved in dry DCM (1 mL). To a separate round-bottom flask under Ar, **5** (38.0 mg, 0.057 mmol, 1.0 eq.) and dry pyridine (23 µL, 0.286 mmol, 5.0 eq.) were dissolved in dry DCM (2 mL), and the solution was added dropwise into the first solution. The reaction was stirred at r.t. for 16 h. The reaction was concentrated under reduced pressure and redissolved in dry DMAc (1 mL). To a separate round-bottom flask under Ar, DIBO-NH_2_ (34.7 mg, 0.113 mmol, 2.0 eq.) and DIPEA (50 µL, 0.287 mmol, 5.0 eq.) were dissolved in dry DMAc (2 mL), and then added dropwise into the previous solution. The reaction was stirred at r.t. for 3 h, and then concentrated under reduced pressure. The residue was redissolved in CHCl_3_ (3 mL), and washed with NH_4_Cl sat (3 mL) and brine (3 mL). Each aqueous layer was extracted with CHCl_3_ (3 × 3 mL). The combined organic layers were dried over Na_2_SO_4_, filtered and concentrated under reduced pressure. The crude was purified by FC (Al_2_O_3_ basic III, 4.9% H_2_O) eluting with a ternary gradient of Hexane/[DCM/MeOH (20:1)] (1:1 to 1:7) to retrieve the product as a light-orange oil (32.1 mg, 0.032 mmol, 56%).

**HRMS** (ESI/QTOF) m/z: [M + H]^+^ Calcd for C_5_4H_67_N_8_O_11_^+^ 1003.4924; found 1003.4896.

**^1^H-NMR** (400 MHz, Acetonitrile-*d*_3_) δ 7.94–7.89 (m, 2H, Py-*H*), 7.88–7.80 (m, 2H, Py-*H*), 7.74–7.69 (m, 2H, Py-*H*), 7.60–7.51 (m, 1H, DIBO-Ar*H*), 7.42–7.28 (m, 7H, DIBO-Ar*H*), 6.35–6.25 (m, 2H, N*H*), 5.86–5.81 (m, 1H, N*H*), 5.37–5.29 (m, 1H, DIBO-C*H*), 4.48–4.40 (m, 1H, C*H), 4.35 (q, *J* = 7.1 Hz, 4H, O-C*H*2-CH3), 4.14–4.03 (m, 2H, CH2-C*H2*-C*H), 4.02–3.92 (m, 2H, CH2-C*H2*-OCON), 3.83 (s, 4H, Py-C*H*2-N), 3.24–3.08 (m, 5H, NH-C*H*2-C*H*2-NH and DIBO-C*H*2), 2.87–2.63 (m, 13H, N-C*H*2-C*H*2-N and DIBO-C*H*2′), 2.55–2.46 (m, 2H, N-C*H*2-CH2-C*), 2.14–2.09 (m, 2H, CO-C*H*2-CH2), 1.88–1.75 (m, 2H, CH2-C*H*2-CH2), 1.34 (t, *J* = 7.1 Hz, 6H, O-CH2-C*H*3), 1.18 (t, *J* = 7.0 Hz, 3H, O-CH2-C*H*3).

**^13^C-NMR** (101 MHz, CD_3_CN) δ 165.80, 153.06, 151.98, 148.07, 137.99, 130.75, 128.94, 128.87, 127.86, 127.83, 127.74, 127.07, 126.74, 126.45, 124.59, 123.96, 123.77, 121.42, 113.14, 110.34, 76.65, 64.24, 61.92, 61.35, 54.91, 46.55, 42.32, 41.36, 40.31, 32.51, 25.48, 14.18, 14.13.

#### 2.1.7. **H_3_L^D^** Ligand

To a round-bottom flask, **6** (32.7 mg, 0.033 mmol, 1.0 eq.) was dissolved in EtOH (4 mL) and H_2_O (0.5 mL). To a separate round-bottom flask, LiOH (2.58 mg, 0.108 mmol, 3.3 eq.) was dissolved in H_2_O (0.5 mL), and the solution was added dropwise to the first solution. The reaction was stirred at r.t. for 4 h. EtOH was evaporated under reduced pressure and the aqueous layer was acidified with 1M HCl (pH < 2). The solution was then purified by dialysis against H_2_O/EtOH (9:1) (4× 3 h cycle), and then against H_2_O (4× 3 h cycle). The solution was lyophilized to retrieve the product as a light-brown solid (27.1 mg, 0.029 mmol, 90%).

**HRMS** (ESI/QTOF) m/z: [M + H]^+^ Calcd for C_48_H_55_N_8_O_11_^+^ 919.3985; found 919.4017.

**IR** (ATR with MeOH, cm^−1^): ν = 3368 (br), 2956 (m), 2921 (m), 2852 (m), 2153 (w), 1697 (m), 1619 (s), 1586 (s), 1548 (m), 1461 (m), 1438 (m), 1388 (m), 1263 (m), 1150 (w), 1115 (w), 1084 (w), 1036 (w), 889 (w), 761 (m), 678 (w).

**^1^H-NMR **(400 MHz) (400 MHz, Methanol-*d*_4_) δ 8.02–7.95 (m, 2H, H3-3′), 7.91–7.82 (m, 2H, H4-4′), 7.61–7.53 (m, 1H, H-DIBO), 7.50–7.43 (m, 2H, H5-5′), 7.42–7.26 (m, 7H, H-DIBO), 5.44–5.37 (m, 1H, H26), 4.29–4.21 (m, 1H, H16), 4.15–3.93 (m, 6H, H7+H21), 3.26–2.94 (m, 13H, H8-9-10-11-12-13+H27), 2.83–2.75 (m, 1H, H27′), 2.75–2.63 (m, 4H, H23-24), 2.41–2.31 (m, 2H, H14), 1.94–1.79 (m, 4H, H15+H19), 1.36–1.32 (m, 2H, H20).

The remaining signals are attributed to impurities from the FC eluting solvents, i.e., DCM, alkanes and light alcohols, or to traces of non-hydrolyzed starting materials evidenced by ethyl esters signals. HPLC purification of advanced intermediates might help achieve higher purity but was not attempted due to the low product amounts available.

**^13^C-NMR** (101 MHz, Methanol-*d*_4_) δ 175.03 (C17), 172.17 (C3-3′), 158.34 (C18), 155.59 (C22), 153.60 (C-33 or C41), 152.49 (C25), 139.57 (C4-4′), 131.18 (C-DIBO), 129.35 (C-DIBO), 128.33 (C-DIBO), 128.26 (C-DIBO), 127.14 (C-DIBO), 126.49 (C5), 125.15 (C-DIBO), 124.28 (C3-3′), 122.33 (C28 or C33 or C36 or C41), 113.81 (C28 or C33 or C36 or C41), 110.98 (C28 or C33 or C36 or C41), 77.95 (C26), 73.84 (C34 or C35), 69.37 (C34 or C35), 65.09 (C21), 62.28 (C7), 55.49 (C-TACN), 53.72 (C16), 51.63 (C-TACN), 47.18 (C27), 41.79 (C-TACN), 41.65 (C-TACN), 33.06 (C14), 28.76(C15 or C19), 26.20 (C15 or C19), 23.72 (C20).

The two missing C-TACN, C23 and C24 presumably are hidden by the solvent peaks. The remaining signals’ chemical shifts are in agreement with the hypothesized structures, supporting the aforementioned impurities.

#### 2.1.8. **[LnL^A^]** Complexes

The procedure was adapted from our previous method. The analytical result was in accordance with previously reported data [56].

The pH of a solution of **H_3_L^A^** (12.5 mg, 21.5 µmol, 1.0 eq.) in H_2_O (1 mL) was adjusted between 5 and 6 with 0.1M HCl or 0.1M NH_4_OH. Separately, LnCl_3_.6H_2_O (25.8 µmol, 1.2 eq.) was dissolved in H_2_O (200 µL), the pH was adjusted similarly, and the solution was then added to the reaction. The reaction was stirred at 37–45 °C for 16 h, and the pH was maintained between 5 and 7 by 0.1 M NH_4_OH addition. The reaction was transferred to a dialysis tube (MWCO 500–1000 Da) and purified against EtOH/H_2_O 1:9 (3 cycles) and H_2_O (3 cycles). The solution was lyophilized to retrieve the complexes as a yellow solid in quantitative yields.

**HRMS** (ESI/QTOF): m/z calcd for C_29_H_34_EuN_6_O_7_^+^ ([M + H]^+^): 731.1696; found 731.1691.

**HRMS** (ESI/QTOF): m/z calcd for C_29_H_34_GdN_6_O_7_^+^ ([M + H]^+^): 736.1652; found 736.1657.

**HRMS** (ESI/QTOF): m/z calcd for C_29_H_34_N_6_O_7_Yb^+^ ([M + H]^+^): 752.1872; found 752.1888.

**HRMS** (nanochip-ESI/LTQ-Orbitrap): m/z calcd for C_29_H_33_N_6_NaO_7_Tb^+^ ([M + Na]^+^): 759.1556; found 759.1575.

#### 2.1.9. **[LnL^D^]** Complexes

The pH of a solution of **H_3_L^D^** (13.5 mg, 14.69 µmol, 1.0 eq.) in H_2_O (400 µL) was adjusted between 5 and 6 with 0.1M HCl or 0.1M NH_4_OH. Separately, LnCl_3_.6H_2_O (17.7 µmol, 1.2 eq.) was dissolved in H_2_O (100 µL), the pH was adjusted similarly, and the solution was then added to the reaction. The reaction was stirred at 37–45 °C for 24 h, and the pH was maintained between 5 and 7 by 0.1 M NH_4_OH addition. The reaction was transferred to a dialysis tube (MWCO 500–1000 Da) and purified against EtOH/H_2_O 1:9 (3 cycles) and H_2_O (3 cycles). The solution was lyophilized to retrieve the complexes as a brown solid in quantitative yields.

**HRMS** (ESI/QTOF) m/z: calcd for C_48_H_52_EuN_8_O_11_^+^ ([M + H]^+^): 1069.2962; found 1069.2986.

**HRMS** (ESI/QTOF) m/z: calcd for C_48_H_51_GdN_8_NaO_11_^+^ ([M + Na]^+^): 1096.2811; found 1096.2863.

**HRMS** (ESI/QTOF) m/z: calcd for C_48_H_52_N_8_O_11_Yb^+^ ([M + H]^+^): 1090.3139; found 1090.3117.

**HRMS** (nanochip-ESI/LTQ-Orbitrap): m/z calcd for C_48_H_51_N_8_NaO_11_Tb^+^ ([M + Na]^+^) 1097.2823; found 1097.2846.

### 2.2. HNP Surface Modifications

#### 2.2.1. Bare LNO HNP Synthesis

Solvothermal preparation of phase-pure lithium niobate (LiNbO_3_, LNO) nanocrystals has been performed according to the recently detailed aqueous alkoxide route [67]. Briefly, in a standard procedure, ethoxides of niobium (Nb(OCH_2_CH_3_)_5_, 99.95% trace metal basis, Sigma-Aldrich, Buchs, Switzerland) and lithium (LiOCH_2_CH_3_, at 1.0 M in ethanol, Sigma-Aldrich) were first stored under argon in a glove box to avoid any unwanted reaction with air. In a typical synthesis, niobium ethoxide (154 μL, 0.6 mmol) was first diluted under argon in 3.75 mL of absolute ethanol before the addition of lithium ethoxide (600 μL, 0.6 mmol). Homogenization with magnetic stirring for 5 min was then carried out outside of the glove box thus resulting in a transparent yellow solution. In this study, 1,4-butanediol (2.25 mL) was the only co-solvent used to promote ligand exchange with the ethoxy groups. To promote condensation and formation of a homogeneous sol–gel solution, a mixture of 100 μL of distilled water and 900 μL of absolute ethanol was then added dropwise. After 24 h of stirring at room temperature, the reaction medium is solvothermally treated at 235 °C for 1 day within a 23 mL Teflon-lined stainless-steel autoclave (Parr Instruments Co., Moline, IL, USA). After cooling down to room temperature, the as-obtained nanocrystals were isolated from the reaction medium upon centrifugation (13,500 rpm) before being redispersed twice in ethanol for additional washing and centrifugation. After drying at 100 °C for 1–2 h, LNO nanocrystals can be easily redispersed under sonication to prepare colloidal suspensions.

XRD patterns (Figure S16a,b), confirming the absence of any amorphous contribution in the prepared samples, were measured from two PANalytical X’Pert^3^ powder diffractometers (Malvern Panalytical, Palaiseau, France) either equipped with a Co or a Cu anticathode and with a rotating zero-background silicon sample holder. The apparent nanocrystal size S_hkl_ was estimated from the peak broadening for a given [hkl] direction after extraction of the integrated intensities within FullProf and according to the Le Bail global fitting procedure. A pseudo-Voigt function and a platelet morphology were assumed to fit the anisotropic broadening of the different XRD peaks after a careful assessment of the instrument resolution previously obtained from a Si calibration powder.

Transmission electron microscopy (TEM) images (Figure S16c,e) were acquired from a JEOL 2100 HT apparatus operating at 200 kV (JEOL Europe SAS, Croissy Sur Seine, France) to further estimate the mean nanocrystal size and size distribution. Size histograms (Figure S16d,f) were obtained from the surface analysis of at least 100 nanocrystals.

#### 2.2.2. Coated LNO Intermediate Synthesis

**LNO@Si-Talys** were prepared following a reported procedure [68]. The size, polydispersity index (PDI) and ZP (Table S1) at each surface modification step were in accordance with reported data. It is important to point out that **LNO@Si-Talys** batches in this work were prepared from two different bare LNO HNPs batches (LNO S2 and LNO S95), which initially have slight variations in size. Entries 1 to 3 in Table S1 correspond to the preparation of **LNO@[GdL^D^]** used in MRI studies, and entries 4 to 5 correspond to the **LNO@[LnL^A^]** and **LNO@[LnL^D^]** preparations for photoluminescence experiments.

#### 2.2.3. **LNO@[LnL^A^]**—CuAAC Conjugation

The procedure was adapted from our previous method using solutions and solvents that were freshly degassed with argon for 30 min, except for the HNP stock suspension. The characterization was in accordance with previously reported data [56].

**LNO@Si-Talys** from stock in EtOH (1 mg) were added to a glass tube, centrifuged (4255 rcf, 10 min, 20 °C) and the supernatant was discarded. The LNO was washed with freshly degassed EtOH and then resuspended in EtOH (200 μL). A CuSO_4_.5H_2_O (0.25 mg, 1 µmol, 0.5 eq.) and THPTA (0.43 mg, 1 µmol, 0.5 eq.) solution in H_2_O (100 µL), and a **[LnL^A^]** (2 µmol, 1 eq.) solution in H_2_O/DMSO (1:1, 100 µL) were added to the LNO. A NaAsc (0.9 mg, 4.5 µmol, 2.25 eq.) solution in H_2_O (100 µL) was then added. The reaction was stirred at r.t. for 4 h. The mixture was centrifuged (4255 rcf, 10 min, 20 °C) and the supernatant discarded. The residue was washed with H_2_O (2×) and EtOH (2×), dried under a N_2_ flux, and the solid residue was resuspended in EtOH (500 µL, 2 mg/mL) for storage at 4 °C. Aliquots (10 µL, 0.02 mg) were diluted with relevant dispersants (1 mL), sonicated for 1 min and analyzed by DLS. Complementary aliquots were diluted in EtOH for FTIR (0.2 mg in 200 μL) and STEM-EDX (200 μL at 0.5 mg/mL).

#### 2.2.4. **LNO@[LnL^D^]**—SPAAC Conjugation

To a glass centrifugation tube containing a suspension of **LNO@Si-Talys** (1 mg) in EtOH (200 µL), DMAc/H_2_O (19:1) (200 µL) was added. A suspension of **[LnL^D^]** (1.3 µmol) in EtOH/[DMAc/H_2_O (19:1)] (1:1) (600 µL) was then added, the tube sealed and the reaction stirred at 40 °C for 24 h. The reaction mixture was centrifuged (4255 rcf, 10 min, 20 °C) and the solid residue was washed with EtOH/DMAc (1:1) (4×) and heptane (2×). The LNOs were resuspended in EtOH (500 µL, 2 mg/mL) for storage at 4 °C. Aliquots (10 µL, 0.02 mg) were diluted with relevant dispersants (1 mL), sonicated for 1 min and analyzed by DLS. Complementary aliquots were diluted in EtOH for FTIR (0.2 mg in 200 μL) and STEM-EDX (200 μL at 0.5 mg/mL).

#### 2.2.5. **LNO@[GdL^D^]** Recycling into **LNO@[TbL^D^]**

To a glass centrifugation tube containing a suspension of **LNO@[GdL^D^]** (0.9 mg) in H_2_O (100 μL), 0.05M HCl (1.4 mL) was added. The suspension was stirred at rt for 2 h, the reaction mixture was centrifuged (4255 rcf, 10 min, 20 °C) and the solid residue was washed with H_2_O (3×). The HNPs were resuspended in H_2_O at pH 5.5–6.5 (1 mL). TbCl_3_.6H_2_O (1 mg/mg HNP) dissolved in H_2_O at pH 5.5–6.5 (100 μL) was then added, the tube sealed, and the reaction stirred at 40 °C for 16 h. The reaction mixture was centrifuged (4255 rcf, 10 min, 20 °C) and the solid residue was washed with H_2_O (3×). An aliquot (0.4 mg) was collected for luminescence analysis and the remaining volume was further washed with EtOH (1×) and heptane (1×), and then resuspended in EtOH (250 μL, 2 mg/mL) for storage at 4 °C. Aliquots (10 µL, 0.02 mg) were diluted with relevant dispersants (1 mL), sonicated for 1 min and analyzed by DLS.

### 2.3. MRI Phantom Imaging

**LNO@[GdL^D^]** (suspended in water, 5 mg/mL) was mixed with a transparent solution of agarose (1.5%) in PBS (pH = 7.4, 144 mg/L KH_2_PO_4_, 9000 mg/L NaCl, 795 mg/L Na_2_HPO_4_·7H_2_O) at 11 different concentrations: 0.00, 0.01, 0.05, 0.10, 0.15, 0.20, 0.25, 0.50, 0.75, 1.00, and 1.25 mg/mL (tubes 1 to 11, respectively). The agarose gels were transferred into 5 mm NMR tubes. The *T*_1_ and *T*_2_ relaxation constants were measured at 37 °C and 14.1 T (600 MHz for 1H) on a Bruker spectrometer equipped with a 5 mm CPPBBOz probe using saturation recovery and CPMG sequences. To avoid relaxation damping during saturation due to high 1H concentration, the probe was slightly detuned. The variable *T*_1_ relaxation delays were set to 0.001, 0.010, 0.050, 0.075, 0.100, 0.250, 0.500, 0.750, 2.5, 5, 10, and 25 s. The relaxation times *T*_1_ were extracted, fitting the data with a mono-exponential build-up. The *T*_2_ echo delay was set to 2 ms, and the variable CPMG loop counters were set to 2, 4, 6, 8, 10, 12, 16, 24, 32, 48, 64, and 128. The relaxation times *T*_2_ were extracted, fitting the data with a mono-exponential decay. For MRI, the NMR tubes were then inserted into a homemade Teflon NMR tube holder phantom. The MRI experiments were performed on a 14.1 T magnet (Magnex Scientific, Yarnton, UK) equipped with a 1 T/m peak strength and 5500 T/m/s slew rate shielded gradient set (Resonance Research, Billerica, MA, USA) interfaced to a Bruker console (BioSpec AVANCE Neo system using ParaVision 360 software, Bruker BioSpin, Ettlingen, Germany). A RAPID Biomedical linearly polarized volume coil (RAPID Biomedical GmbH, Rimpar, Germany) was used for transmission and reception (74 mm inner diameter, 75 mm resonator length).

2D *T*_1_-weighted contrast images were collected using a gradient-echo sequence/fast low-angle shot (FLASH) with the following parameters: TE/TR = 2.75/500 ms, flip angle = 45°, matrix size = 186 × 186, FOV = 27.9 × 27.9 mm^2^, 9 axial 1.5 mm slices, and 1 average. An ultra-short TE was chosen to minimize *T*_2_ weighting, while a medium TR of 500ms was selected to be sensitive to *T*_1_ in the expected relaxation value range.

2D *T*_2_-weighted contrast images were collected using a spin-echo TurboRARE sequence with the following parameters: TE/TR = 12/5000 ms, excitation flip angle = 90° and refocusing flip angle = 136.42°, matrix size = 186 × 186, FOV = 27.9 × 27.9 mm^2^, 9 axial 1.5 mm slices, and 1 average. The repetition time (TR) was chosen to minimize the *T*_1_ contrast (fully relaxed signal), while a moderate TE was selected to be sensitive to *T*_2_ in the expected relaxation value range.

### 2.4. Photophysical Property Investigation

The emission and excitation spectra were recorded at room temperature on a Horiba-Jobin Yvon Fluorolog FL-3-22 fluorimeter (Unterhaching, Germany) equipped with CW 450W Xenon source for fluorescence mode and UV xenon flash tube for phosphorescence. Data were collected by using a thermoelectrically cooled R2658P PMT (Hamamatsu, Unterhaching, Germany; range 220–1010 nm) or a NIR PMT (950–1700 nm, thermoelectrically cooled H10330-75 NIR-PMT; Hamamatsu, Unterhaching, Germany). The spectra were corrected by the instrumental correction function. Data processing was performed with the program Origin 8^®^. Lifetime decays were recorded by using TCSPC Delta time unit, using a xenon flash tube source. The data were analyzed by using DAS software (version 6), by using a mono-, bi- or tri-exponential function, and the best fits were kept. They are the averages of at least three independent measurements. Quantum yield measurements were performed using a G8 GMP integrating sphere [Φ = (Ec − Ea)/(La − Lc), where Ec is the integrated emission spectrum of the sample, Ea is the integrated “blank” emission spectrum, La is the “blank” absorption, and Lc is the sample absorption at the excitation wavelength).

Aerated powders as well as aerated solutions of the samples in DMSO and H_2_O were analyzed in a quartz capillary. The concentration was 4 mM for ligand and complexes, and 2 mg/mL for all nanoparticles.

For spatially resolved measurements, we used a custom-modified confocal Raman microscope *LabRAM HR Evolution* equipped with a fs laser source emitting at 800 nm (Coherent Vitesse, 100~ fs, 80~ MHz). The angle *ϕ* of the linearly polarized light from the laser was tuned by rotating a half-wave plate. The laser beam was transmitted through a nonpolarizing dichroic beamsplitter [69] for precise control of the incident polarization and focused onto the sample by an infinity-corrected objective (Olympus ×100, NA = 0.9). The same objective was employed to collect the emitted signals in epi-detection. After propagation through the nonpolarizing dichroic beamsplitter, second-harmonic generation (SHG) and photoluminescence (PL) were spectrally separated and analyzed in two separate channels. The first one was used for direct detection of the SHG signal by a CCD detector through a 10 nm width bandpass filter centered at 400 nm and an analyzer oriented parallel (*ϕ*) or orthogonal (*ϕ + π*/2) to the excitation polarization. This approach allows analyzing the full polarization-resolved response of SHG. On the other hand, the PL spectra were acquired without detection analyzer for greater sensitivity. In this case, the signal was processed by line subtraction of the background followed by spectral integration of the band.

The **LNO@[EuL^A^]** suspension in EtOH (0.1 mg/mL) was dropped (5 μL) at an angle on a glass slide to create a density gradient and the solvent was evaporated at rt for 5 min.

## 3. Results and Discussion

### 3.1. Synthesis of the **H_3_L^D^** Ligand

**Scheme 1:**
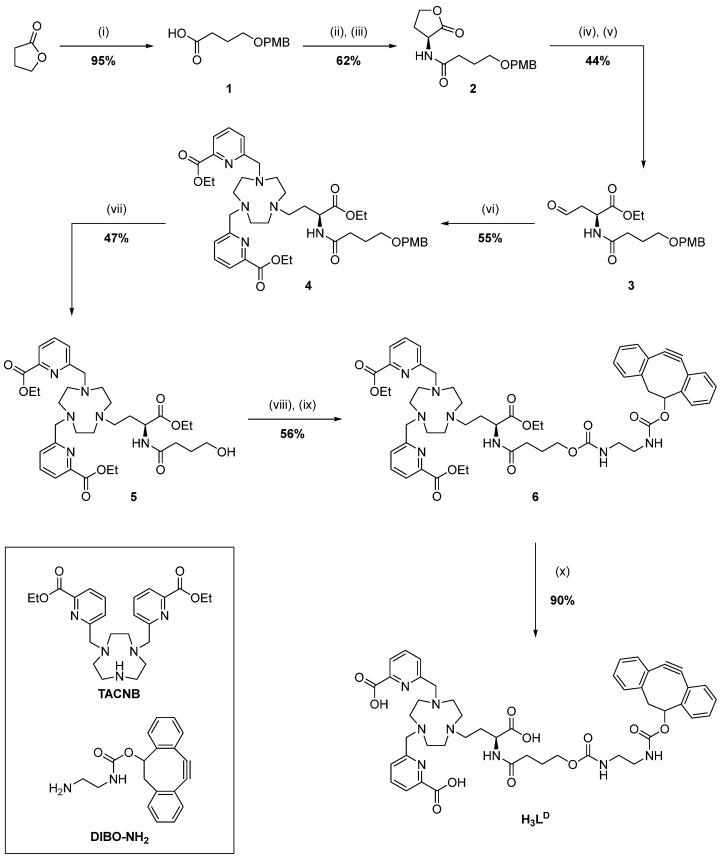
**H_3_L^D^** synthesis. (i) PMBBr, KOH, Tol, reflux; (ii) DCC, NHS, THF, r.t.; (iii) (S)-(−)-α-amino-γ-butyrolactone hydrobromide, TEA, DCM, r.t.; (iv) H_2_SO_4_, EtOH, r.t.; (v) (COCl)_2_, DMSO, TEA, DCM, −78 °C; (vi) **TACNB**, AcOH, NaBH(OAc)_3_, DCE, r.t.; (vii) DDQ, DCM/PBS, r.t.; (viii) 4-NPCF, Py, DCM, r.t.; (ix) **DIBO-NH_2_**, DIPEA, DMAc, r.t.; (x) LiOH, DCM/MeOH, r.t.

A new synthetic pathway was designed from the previously established **H_3_L^A^** to replace the terminal alkyne with a strained DIBO that enables copper-free click chemistry via SPAAC (Scheme 1). This approach was successfully applied in the past to conjugate a cellular-targeting ligand [64] and different photoresponsive drug delivery systems [65,70,71] to the surface of HNPs. The synthesis starts with the γ-butyrolactone ring opening using a strong base to generate the corresponding hydroxy acid, whose alcohol position was directly protected with *p*-methoxybenzylbromide (PMBBr) in a one-pot reaction to afford **1** in a nearly quantitative yield. After amide coupling with (S)-(−)-α-amino-γ-butyrolactone hydrobromide, the acid-catalyzed ring opening of **2** and in situ trans-esterification generated an unstable alcohol intermediate, which was successfully oxidized under Swern conditions to the aldehyde **3**. This aldehyde was reacted with the secondary amine position of the previously synthesized 1,4-bipicolinate-1,4,7-triazacyclononane (**TACNB**) via reductive amination in the presence of NaBH(OAc)_3_ to afford **4** in moderate yield. The primary hydroxyl was then selectively deprotected under radical conditions using 2,3-dichloro-5,6-dicyano-1,4-benzoquinone (DDQ) and the resulting alcohol **5** was activated with 4-nitrophenyl chloroformate (4-NPCF) for subsequent coupling with **DIBO-NH_2_** to afford the ester-protected ligand precursor. The final DIBO ligand **H_3_L^D^** was isolated after saponification with LiOH.

### 3.2. **H_3_L^A^** and **H_3_L^D^** Complexes Synthesis

**Scheme 2:**
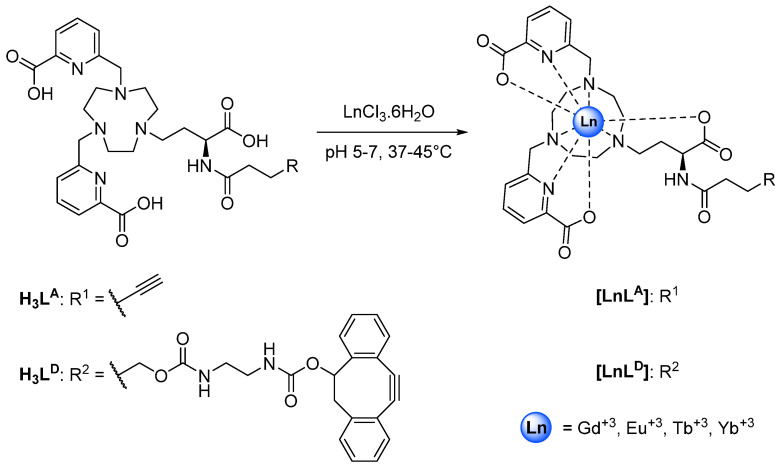
Synthesis of Ln^+3^ complexes in mildly acidic aqueous conditions.

Four trichloride salts based on Gd^+3^, Eu^+3^, Tb^+3^ and Yb^+3^ were selected to cover several imaging modalities, including MRI, red and green visible as well as NIR PL, respectively. Each Ln chloride salt was reacted with both **H_3_L^A^** and **H_3_L^D^** under mild heating and controlled pH conditions (Scheme 2) to afford the corresponding complexes. The consistent complexes’ formation of all four Ln ions by both ligands under similar conditions reflects their versatility toward diverse click handles while maintaining strong Ln binding, mirroring the characteristics of the analogous **H_3_ebpatcn** scaffold despite the presence of an extra methylene group on the third carboxylate [51,55].

### 3.3. LNO Functionalization with Ln Complexes

**Figure 2:**
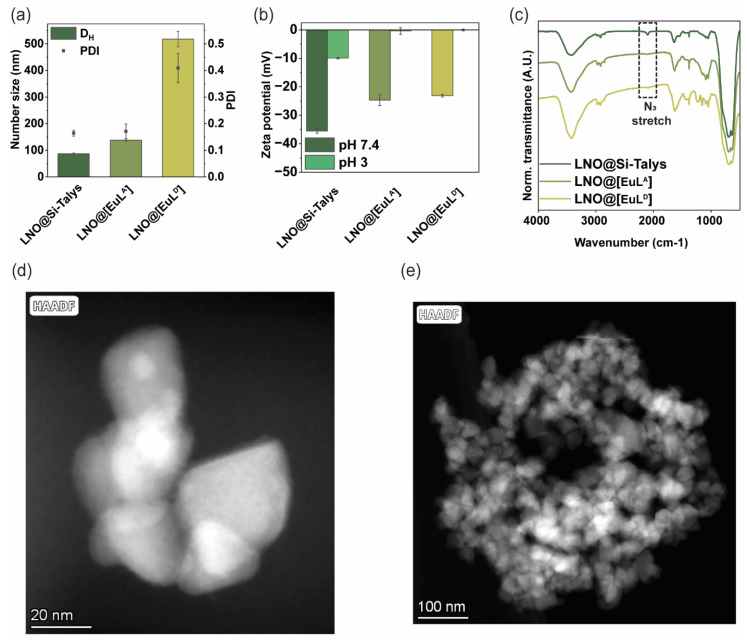

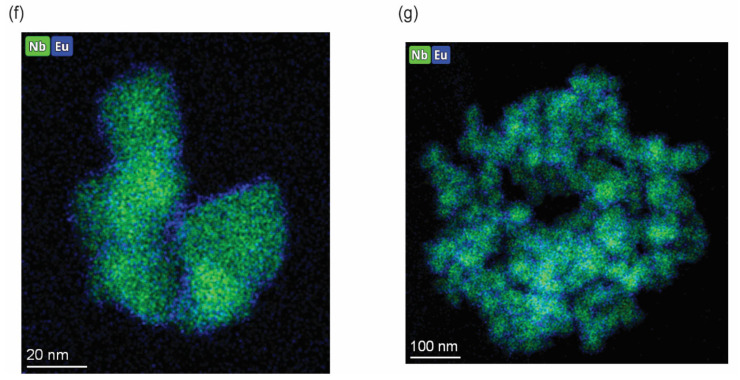
Comparative characterization of representative **LNO@[Eu]** conjugates relative to their parent **LNO@Si-Talys**. (**a**) Number mean D_H_ and PDI values in PBS 0.1X, and (**b**) ZP values at pH 7.4 and 3 (PBS 0.1X). (**c**) FTIR spectra normalized to the Nb-O band (750 cm^−1^), highlighting a decrease in N_3_ band (2100 cm^−1^). STEM HAADF micrographs of (**d**) **LNO@[EuL^A^]** and (**e**) **LNO@[EuL^D^]**, and EDX Nb/Eu elemental maps of (**f**) **LNO@[EuL^A^]** and (**g**) **LNO@[EuL^D^]**.

**Scheme 3:**
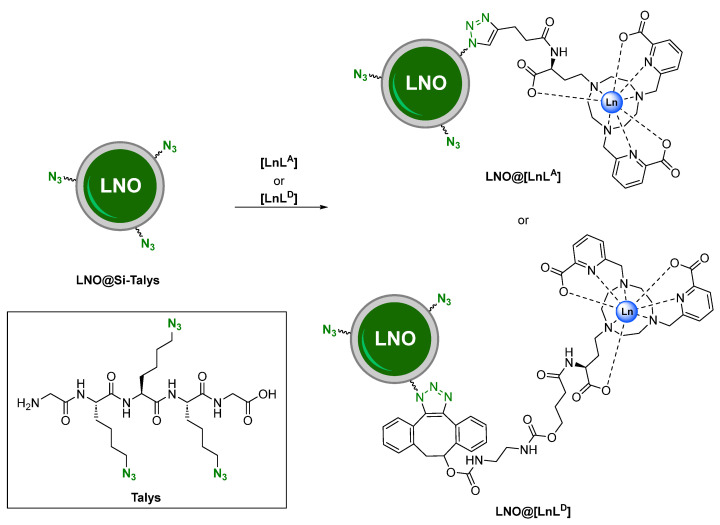
Post-functionalization of LiNbO_3_ (LNO) HNPs via CuAAC or SPAAC using **[LnL^A^]** or **[LnL^D^]**, respectively.

The surface modification of silanized LNO HNPs with a short tri-azidolysine (**Talys**) pentapeptide was previously reported to allow for the simultaneous introduction of surface azido and carboxyl reactive handles while maintaining good colloidal properties both in aqueous and polar organic media [68]. Talys-modified LNO HNPs (**LNO@Si-Talys**) were therefore synthesized (precursor characterization in Supplementary Materials, Table S1), and used for further conjugation with **[LnL^A^]** and **[LnL^D^]** via CuAAC or SPAAC, respectively (Scheme 3). In the case of CuAAC, the aqueous hydrodynamic diameter (D_H_) increased compared to the **LNO@Si-Talys** while the zeta potential (ZP) maintained similar pH-dependent values (Table 1, entries 2–4, Figure 2a,b and Figures S17–S20a), suggesting efficient surface modification without altering the carboxylate groups. The simultaneous D_H_ decrease in EtOH and D_H_ increase in PBS further corroborated a modification in solvation due to the introduction of a neutral moiety, i.e., **[LnL^A^]**. In the case of SPAAC, while a similar trend was observed in EtOH, the size increase in PBS was larger and in agreement with the introduction of a bulkier hydrophobic complex on the HNP surface (Table 1, entries 5–8, Figure 2a,b and Figures S17–S20a). Importantly, the corresponding correlograms (Figures S17–S20b) indicated no sedimentation during measurement, and only suggested aggregation in the cases where the polydispersity index (PDI) was above 0.2. The pH-dependent ZP was maintained as well, indicating functionalization at the azide position. The disappearance of the azide stretching band in FTIR spectroscopy further confirmed azide–alkyne cycloaddition (Figure 2c and Figures S17–S20c). We anticipate that future efforts to incorporate a hydrophilic spacer within **[LnL^D^]** will allow to decrease the aggregation of conjugated LNOs D_H_ in aqueous medium.

The different LNO HNP conjugates were further analyzed by scanning transmission electron microscopy (STEM) in high-angle annular dark field (HAADF) mode and their elemental composition was mapped by energy-dispersive X-ray spectroscopy (EDX) (Figure 2d–g and Figures S17–S20d–k). The samples exhibited a clear core–shell structure with the Ln atoms homogeneously distributed around the LNO core, therefore giving evidence for successful surface functionalization. The size and shape of functionalized LNO HNPs were in accordance with DLS values and previous literature [67,68]. Overall, these data confirm that Ln complexes can be efficiently conjugated to the surface of silanized LNO HNPs using either CuAAC or SPAAC click chemistry.

### 3.4. Validation of MR Nonlinear Optical Dual Imaging Using **LNO@[GdL^D^]**

**Figure 3:**
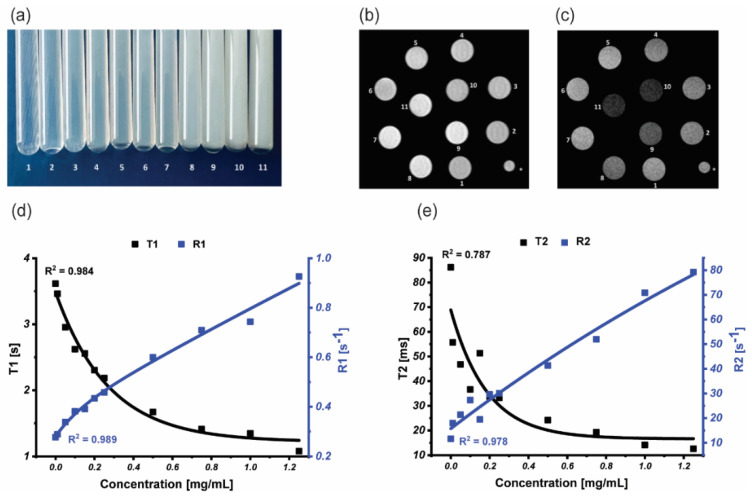

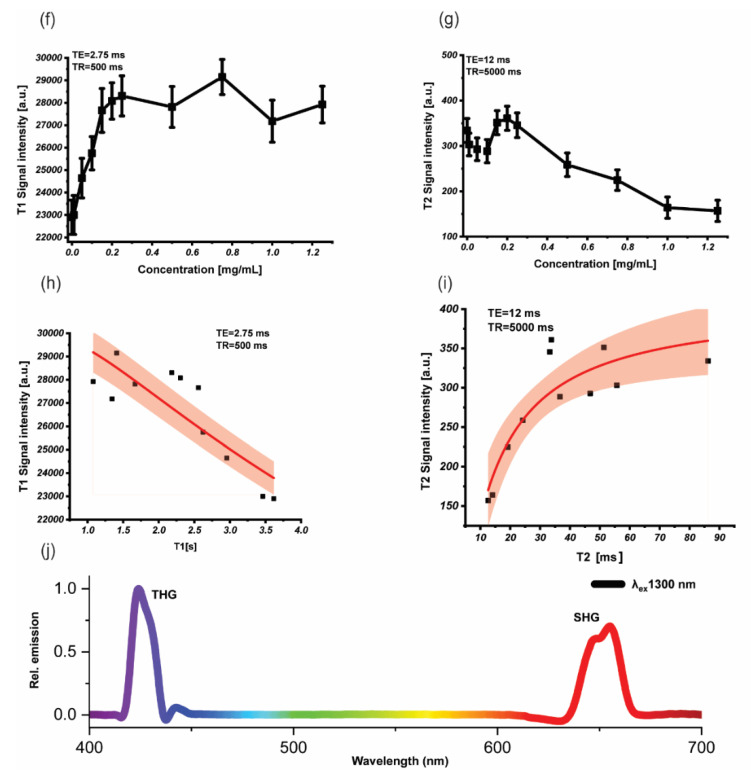
MRI and nonlinear optical response of **LNO@[EuL^D^]**. (**a**) Agarose gel phantom tubes containing 0 to 1.25 mg/mL NPs (exact composition in Section 2.3) and their corresponding (**b**) T_1_-weighted and (**c**) T_2_-weighted contrast images of the columnar phantom, FOV = 27.9 × 27.9 mm^2^, *—water sample. (**d**) T_1_ relaxation times and R1 relaxation rate curve and (**e**) T_2_ relaxation times and R2 relaxation rate curve. (**f**) T_1_ signal intensity quantification curve and (**g**) T_2_ signal intensity quantification curve. (**h**) Realistic monotonic T_1_ signal intensity variation over the T_1_ relaxation time range and (**i**) mono-exponential T_2_ signal intensity dependence over the T_2_ relaxation time range related to the **LNO@[GdL^D^]** NP concentration increase. The fitting parameters are given in Figure S21. (**j**) Emission spectrum upon fs-pulsed excitation at 1300 nm showing SHG and THG peaks. The spectrum was corrected by the spectral sensitivity of the system and response of the substrate to remove the contribution of THG emitted from the air/glass interface. Note that the SHG/THG ratio depends on excitation intensity and polarization used in the measurement.

The magnetic resonance (MR) properties of **LNO@[GdL^D^]** were first investigated following an MRI phantom approach [56]. Briefly, **LNO@[GdL^D^]** were incorporated into an agarose gel phantom at varying concentrations (Figure 3a), and their relaxation properties were measured. These data were compared with the recorded *T*_1_- (Figure 3b) or *T*_2_-weighted (Figure 3c) phantom images. The samples exhibited an almost linear increase in both R1 (Figure 3d) and R2 (Figure 3e) relaxation rates with increasing NP concentration, which in turn translated into a concentration-dependent increase in *T*_1_-weighted MR signal (Figure 3f) and decrease in *T*_2_-weighted MR signal (Figure 3g) on the phantom images, respectively. Strikingly, the contrast magnitude nearly doubled in the *T*_1_-weighted image compared to the reported system based on CuAAC conjugation, while the *T*_2_-weighted image was characterized by a slightly more rapid decrease in signal intensity [56]. The subsequent quantitative phantom mapping of the *T*_1_- and *T*_2_-weighted MR signal intensity variations exhibited a clear mono-exponential signal dependence over the *T*_2_ range (Figure 3i), and a realistic monotonic signal variation over the *T*_1_ range (Figure 3h) related to the NP concentration, respectively, in line with the reported data. These results therefore confirm that **[GdL^D^]** can be conjugated to LNO HNPs via SPAAC resulting in a promising *T*_1_ and *T*_2_ MRI CA. The LNO core’s ability to produce multi-harmonic emission was further evaluated upon femtosecond (fs) pulsed irradiation at 1300 nm, in the second NIR (NIR-II) window. The obtained visible spectrum (Figure 3j) displayed a strong nonlinear response, with simultaneous detection of SHG (650 nm) and THG (433 nm), similarly to reported LNO nanomaterials [71,72,73]. The combined **LNO@[GdL^D^]** nonlinear imaging and MRI properties therefore demonstrate the effectiveness of the SPAAC conjugation pathway relative to the copper-catalyzed version. It also suggests that this approach may further improve the overall performance in a biological environment by reducing the cytotoxicity associated with copper residues. To further develop this type of MRI CA, it would be interesting to investigate the interplay between the overall system hydrophilicity (i.e., the HNP coating and the complex molecular structure) and the observed MRI properties which strongly rely upon water accessibility [31,45,74].

### 3.5. Investigation of Photophysical Properties

**Figure 4:**
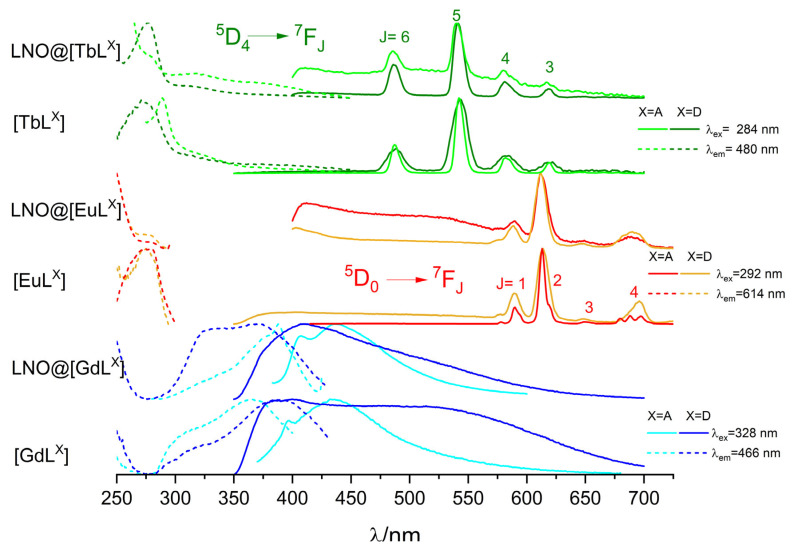
Normalized corrected emission and excitation spectra of the **[LnL^X^]** complexes and **LNO@[LnL^X^]** (X = A or D), at room temperature, in solid state. The wavelengths were chosen at the maximum of emission and excitation spectra, respectively.

We first investigated the ligand-centered emission of the Gd complexes in the solid state. At room temperature, excitation within the ligand absorption band at 328 nm gives rise to a broad emission envelope originating from both singlet ^1^ππ* and triplet ^3^ππ* excited states of the ligand (Figure 4). Under phosphorescence detection conditions, still at room temperature, ligand emission from the singlet state remains observable alongside the triplet-state emission (Figure S23). The latter displays a maximum at ca. 518 nm (19,305 cm^−1^) together with a well-defined 0–0 transition at 470 nm (21,277 cm^−1^), accompanied by vibronic fine structure, consistent with a ligand-centered ring-breathing mode. The corresponding triplet-state lifetime is 0.9 ± 0.1 ms. Upon grafting onto the surface of the NPs, a broad ligand-centered emission is still observed, although significantly broadened and extending up to ca. 700 nm.

The ligand also efficiently sensitizes the luminescence of Eu^3+^ and Tb^3+^ ions in the visible region at room temperature, as illustrated in Figure 4.

For the Eu^3+^ complexes, the excitation spectra are blue-shifted, with maxima at 272 nm for **[EuL^A^]** and 292 nm for the corresponding NP-supported systems. Upon ligand excitation, the emission spectra are dominated by the characteristic metal-centered ^5^D_0_ → ^7^F_J_ (J = 1–4) transitions of Eu^3+^. The ^5^D_0_ → ^7^F_0_ transition appears as a very weak band, while the other ^5^D_0_ → ^7^F_J_ transitions exhibit two, two and four main components for J = 1, 2 and 4, respectively. This splitting pattern is indicative of a single dominant Eu^3+^ species undergoing a low-symmetry crystal field, consistent with a pseudo-D_3_ local symmetry around the metal center.

Notably, the emission profiles and relative intensities of **LNO@[EuL^A^]** match those of the molecular complex **[EuL^A^]**, indicating that grafting onto the NP surface does not significantly perturb the coordination environment of the Eu^3+^ ion. This conclusion is further supported by the mono-exponential emission decays observed for both systems (in Table 2 (solid state) and Table S2 (DMSO and water)). The slightly shorter lifetime observed for the NP-supported complex points to additional non-radiative deactivation pathways at the NP surface.

In addition to the narrow metal-centered emission, residual ligand-centered triplet phosphorescence is observed for both **[EuL^A^]** and **LNO@[EuL^A^]** in the solid state; however, the integrated intensity of the Eu^3+^-centered emission is significantly higher, evidencing efficient ligand-to-metal energy transfer. In DMSO solution, neither the ^1^ππ* nor the ^3^ππ* ligand emission is detected, which can be attributed to reduced aggregation effects in solution and further confirms efficient sensitization of the Eu^3+^ excited states. In contrast, ligand phosphorescence re-emerges in aqueous solution, and Eu^3+^ emission is strongly decreased (Figures S24 and S25), reflecting efficient non-radiative deactivation mediated by O–H vibrational oscillators of the water molecules.

For the Tb^3+^ systems, the characteristic sharp emission lines arising from the ^5^D_4_ → ^7^F_J_ (J = 6–3) transitions are observed for both the molecular complexes and the NP-supported materials. Ligand-centered triplet phosphorescence is also detected for **LNO@[TbL^A^]** in the solid state, but not for **LNO@[TbL^D^]**. For the latter, it is also negligible in water (Figure S27). The luminescence decay of all Tb^3+^-emitting species is mono-exponential in the microsecond range, and is shorter for the **LNO@[TbL^X^]** compared to the complexes.

Efficient sensitization of Tb^3+^ and Eu^3+^ by the ligand depends on the alignment between the ligand triplet energy (^3^ππ*) and the lanthanide emitting levels. For Tb^3+^ (^5^D_4_ ≈ 20,500 cm^−1^), the energy gap ΔE(^3^ππ*-^5^D_4_) is ca. 780 cm^−1^, which lies within the range compatible with efficient energy transfer while minimizing back-transfer, consistent with the intense Tb^3+^ emission observed. In the case of Eu^3+^ (^5^D_0_ ≈ 17,200 cm^−1^), the corresponding energy gap ΔE(^3^ππ*-^5^D_0_) is ca. 4100 cm^−1^. Although this gap is theoretically suitable for sensitization, the Eu^3+^ emission is noticeably weaker, reflecting the generally lower efficiency of ligand-to-Eu^3+^ energy transfer relative to Tb^3+^ in this TACN-containing system.

The photophysical behavior of the lanthanide complexes reported here highlights the intrinsic compromise between ligand design optimized for MRI applications and the requirements for efficient lanthanide-centered luminescence. In particular, the presence of inner-sphere water molecules, which is essential for high relaxivity in Gd^3+^-based MRI CAs, is well known to be detrimental to Eu^3+^ emission due to efficient non-radiative deactivation pathways involving O–H vibrational oscillators, as Eu^3+^ luminescence is extremely sensitive to its first coordination sphere. The presence of one or more coordinated water molecules introduces high-energy vibrational modes that efficiently quench the ^5^D_0_ excited state, leading to shortened lifetimes and reduced quantum yields. This behavior is clearly evidenced in the present systems by the pronounced decrease in both lifetime and emission intensity observed in aqueous media. Furthermore, the hydration number *q* was estimated using established phenomenological equations [75] for **[EuL^A^],** based on the observed luminescence lifetimes in H_2_O and D_2_O, and a value of the hydration number *q* ≈ 1.7 was obtained. Ligands such as DOTA or TACN, which are widely employed in Gd^3+^ MRI CAs, are deliberately designed to leave one coordination site available for water in order to maximize relaxivity, while maintaining high thermodynamic stability and kinetic inertness. Although this design strategy is ideal for MRI, it is intrinsically incompatible with the requirements for efficient Eu^3+^ luminescence, which implies *q* ≈ 0. Consequently, Eu^3+^ complexes derived from such ligand frameworks typically exhibit limited emission efficiencies, as observed in the present study. Interestingly, the reported **[GdL^A^]** relaxivity [56] and the measured emission lifetimes for **[EuL^A^]** and **[TbL^A^]** are still comparable to the corresponding complexes based on the H_3_ebpatcn ligand [55] as well as Eu-conjugated Au NPs [48], highlighting the efficient design compromise to achieve both MR and lanthanide luminescence detection. This also suggests that the coordination properties of the latter are maintained within the new **H_3_L^A^** and **H_3_L^D^** ligands, even though we cannot exclude an equilibrium with the arm of the linker as it forms a seven-member ring. In addition, even in these detrimental conditions, the quantum yield of **[EuL^A^]** amounts to 14.0 ± 0.3% in DMSO, whereas a markedly lower value (<2%) is observed for **LNO@[EuL^A^]**, reflecting additional quenching processes at the NP interface. Although low, this quantum yield remains significant. The additional decrease observed for the NP-supported systems is attributed to surface-related quenching processes, including enhanced vibrational coupling and possible energy migration towards non-radiative traps.

**Figure 5:**
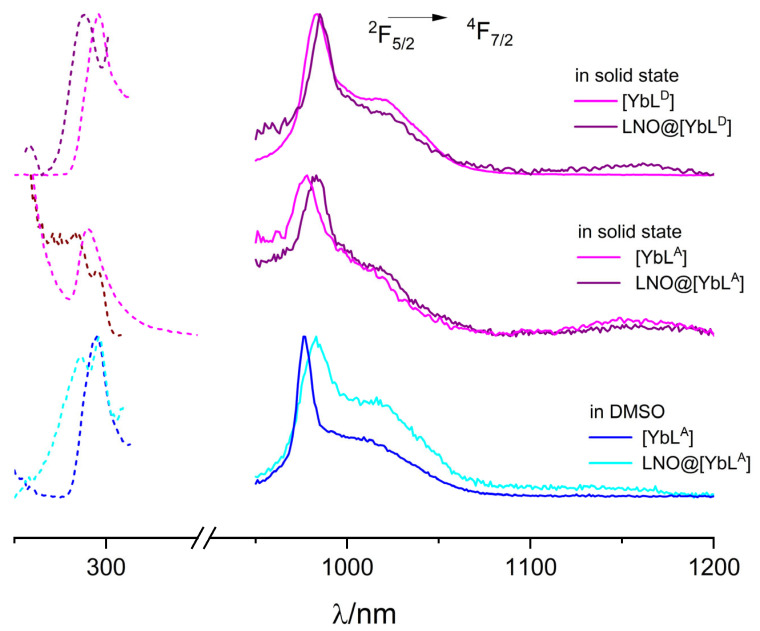
Normalized corrected emission and excitation spectra of the **[YbL^X^]** complex and NPs, at room temperature, in solid state and in solution in 4 mM DMSO; λ_ex_ = 295 nm; λ_em_ = 984 nm.

In the case of the Yb complex and functionalized LNOs, the characteristic emission of Yb (III) in the NIR region at 900–1100 nm can be observed, for both **[YbL^X^]** and **LNO @[YbL^X^]** compounds (X = A, D), in solid state and in solution (Figure 5). The emission is corresponding to the ^2^F_5/2_ → ^2^F_7/2_ transition. Although the intensity of the emission is lower for the NP, it is easily observed at room temperature. This observation also confirms that the complexation and surface conjugation was successful in the case of the Yb, and shows how multiple emission wavelengths can be achieved through variation in the lanthanide species. Another interesting perspective for the **LNO@[YbL^X^]** resides in the fact that Yb (III) complexes are reported to behave as efficient X-ray CT CAs, which could pave the way for a supplementary imaging modality for the nanoplatform [26].

### 3.6. Proof-of-Concept of SHG-Induced Eu Luminescence

**Figure 6:**
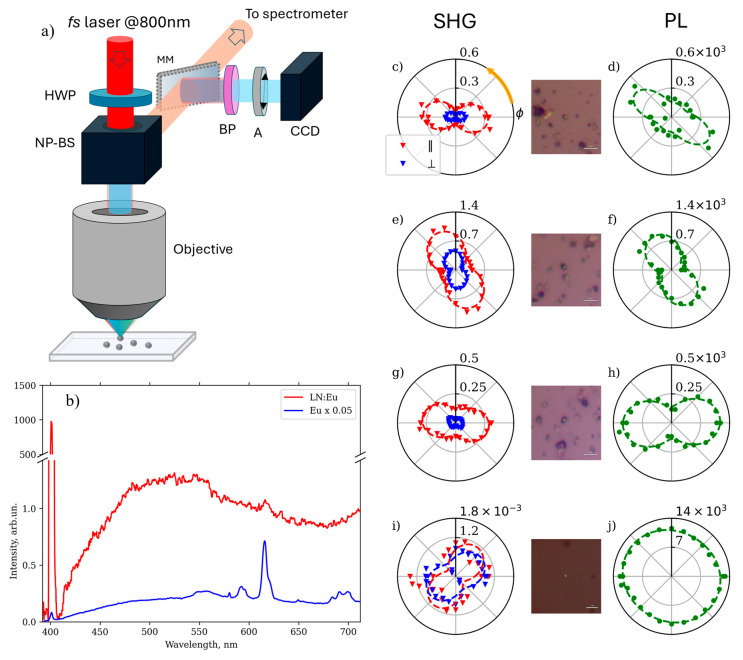
(**a**) Experimental setup used for studying SHG and PL responses of **LNO@[EuL^A^]** NP. NP-BS—nonpolarizing dichroic beamsplitter, HWP—half-wave plate for control of input polarization angle ϕ, A—analyzer oriented at angle ϕ for parallel (||) and ϕ +π/2 for crossed (⟂) SHG detection mode. BP—bandpass filter (center at 400 nm and 10 nm bandwidth), MM—movable dichroic mirror for switching between SHG and spectroscopic detection channels. Focusing was performed with 100× objective (NA = 0.9). (**b**) Spectral response of a single **LNO@[EuL^A^]** subdiffraction-limited particle (red trace) and of the reference **[EuL^A^]** sample (blue trace). The polar plots show a comparison of SHG and PL intensities as a function of polarization angle ϕ for (**c**–**h**) isolated particles and (**i**,**j**) a homogeneous **[EuL^A^]** film. The central panels correspond to brightfield 10 μm × 10 μm images with the position of the polarization-resolved acquisition in the center of the frame.

To study the feasibility of truly combining the Ln complex PL with the nonlinear optical properties of the LNO core, we sought to sensitize the Ln center in conjugated LNOs via their SHG. To circumvent the inaccessible ligand excitation maximum (λ_ex_ = 284–292 nm) with the available laser source, we opted for a direct excitation of the Ln center instead, even though the resulting PL efficiency is intrinsically lower compared to the antenna effect approach employed in the previous section. This approach can be achieved using the **^5^L_6_** energy level of Eu. For spatially resolved measurements, we used a custom-modified confocal Raman microscope *LabRAM HR Evolution* equipped with a fs laser source emitting at 800 nm (Coherent Vitesse, 100~ fs, 80~ MHz). In Figure 6b, we present a comparison of the spectra from a **LNO@[EuL^A^]** subdiffraction-limited particle and a reference, LNO-free sample containing **[EuL^A^]**. The latter (blue trace) presents a characteristic PL peak at 612 nm over a broad PL background extending over the full detection spectral range and a comparatively very weak SHG peak at 400 nm. A **LNO@[EuL^A^]** particle measured under the same conditions shows the opposite trend (red trace), yielding a PL peak at 612 nm about four orders of magnitude weaker than the SHG peak. The difference in intensity can be explained by the low concentration of surface Eu complexes in **LNO@[EuL^A^]** and by the intrinsically lower PL efficiency associated with the experimental design. The ^5^D_0_ → ^7^F_2_ signal is indeed similar to the one observed upon continuous, direct excitation of the Eu cation, without any antenna effect, as depicted in Figure S26. The broadband PL in **LNO@[EuL^A^]** spectrum can be explained by emission from the organic coating as previously reported [71].

The excitation of Eu PL in **LNO@[EuL^A^]** particles can simultaneously occur upon direct two-photon absorption by Eu or one-photon absorption of SHG emitted by the LNO core. To demonstrate that this second excitation channel mediated by LNO emission is also active, we devised an experimental protocol based on polarization-resolved modulation of SHG, as a simple power-dependence measurement would not allow to distinguish the two processes. The selected approach relies on previous works by our and other groups highlighting the strong excitation polarization dependence in the emission of HNPs [76,77,78].

A schematic representation of the experimental setup is presented in Figure 6a. Examples of polarization-resolved SHG and PL intensities, as well as polarization-resolved PL maps, are shown in Figure 6. Different **LNO@[EuL^A^]** NPs display clear polarization-dependent SHG (Figure 6c,e,g) and PL (Figure 6d,f,h) responses that broadly correlate with each other, whereas the reference sample of **[EuL^A^]** shows polarization-independent PL (Figure 6j) and unresolvable SHG (Figure 6i). The maxima of PL and SHG signals correspond to different orientations of the NP crystal structure. It has to be noted that PL and SHG signals do not vanish at polarization angles orthogonal to the LNO *c*-axis, which can be explained by the three-dimensional alignment of the particles and by the complex distribution of the pump field on the substrate due to tight focusing of the beam [79]. In addition, PL excitation can be obtained upon direct multiphoton absorption independently of the presence of the LNO core, adding a polarization-independent component to the total response as in the control measurement of Figure 6j. Figure S22 further highlights that PL and SHG are colocalized and associated with the presence of NPs. Overall, these results constitute a demonstration of Ln PL predominantly triggered by SHG, providing foundations for future developments of optical probes based on these signals. We further anticipate that system optimizations to enable antenna sensitization by a better matching of SHG with Ln complex absorption will significantly increase the PL efficiency.

### 3.7. Recyclability of the **LNO@[LnL^X^]** System

We ultimately established a proof-of-concept experiment highlighting the potential to recycle the **LNO@[LnL^X^]** nanoplatform by modifying its imaging signature. The Gd ion of **LNO@[GdL^D^]** was uncomplexed upon acidic treatment, and the HNPs were then reacted with TbCl_3_. The obtained **LNO@[Gd → TbL^D^]** exhibited spectral properties identical to those of freshly prepared **LNO@[TbL^D^]** (Figure S27). This observation provides evidence for surface functionalization robustness, enabling the preservation of efficient Ln binding abilities to iteratively introduce different Ln ions according to the intended application. This feature opens up promising perspectives for the development of tunable bioimaging [80] and anti-counterfeiting [14] probes based on mixed Ln tags.

## 4. Conclusions

This work describes the synthesis and evaluation of two Ln ligands incorporating either a strained cyclooctyne moiety (**H_3_L^D^**), or a terminal alkyne analog (**H_3_L^A^**), amenable to SPAAC and CuAAC click chemistry, respectively. Thorough NMR analysis confirmed the ligand structure, while HRMS and PL evidenced successful Ln complexation. The photophysical properties of the ligands, their Ln complexes and LNO surface functionalization were indeed explored with particular emphasis towards multimodal imaging. Besides the standard DLS, FTIR and STEM characterizations, an EDX elemental mapping allowed to confirm the homogenous surface distribution of the complexes using both Cu-free and Cu-catalyzed click chemistry. The SPAAC-conjugated Gd system was first studied for nonlinear optical-MR bimodal imaging, demonstrating the ability of these HNP conjugates to act as dual *T*_1_*/T*_2_ MRI CA while preserving their capacity to produce strong SHG and THG signals upon NIR light irradiation. Furthermore, the photophysical properties of all eight Ln complexes and their conjugates were studied, demonstrating the characteristic green Tb, red Eu and NIR Yb emissions, as well as triplet-state Gd emission. In addition, both freshly conjugated **LNO@[LnL^X^]** (X = A, D) samples and recycled batches exhibited luminescence, suggesting high potential for development into a reusable and tunable tagging platform. The Eu signal could further be triggered by using the SHG of the LNO core upon fs-pulsed excitation at 800 nm, providing the first example of a truly multidimensional HNP-Ln conjugate. Considering the wide application range of Ln complexes, this work not only paves the way for multimodal bioimaging combining nonlinear optics, Ln luminescence footprints and/or MRI, but also provides a synthetic framework for future research in areas like sensing [35,40,81], nuclear medicine [82] or anti-counterfeiting [83,84].

## Figures and Tables

**Table 1:** DLS characterization of **LNO@Si-Talys** and **LNO@[LnL^X^]** (X = A, D).

Entry	Sample	EtOH		PBS 0.1X		Zeta Potential (mV)	
		D_H_ ^[a]^ (nm)	PDI	D_H_ ^[a]^ (nm)	PDI	pH 7.4	pH 3
**1**	**LNO@Si-Talys**	364.5 ± 54.1	0.16 ± 0.02	86.9 ± 3.1	0.16 ± 0.01	−35.5 ± 0.8	−9.9 ± 0.3
**2**	**LNO@[GdL^A^]**	191.5 ± 16.8	0.26 ± 0.01	143.6 ± 13.5	0.24 ± 0.01	−35.1 ± 1.1	0.0 ± 0.0
**3**	**LNO@[EuL^A^]**	221.9 ± 52.8	0.30 ± 0.02	138.3 ± 4.8	0.17 ± 0.03	−24.7 ± 1.9	−0.3 ± 1.2
**4**	**LNO@[YbL^A^]**	182.3 ± 11.0	0.19 ± 0.03	144.0 ± 17.6	0.25 ± 0.07	−32.3 ± 1.8	4.3 ± 0.4
**5**	**LNO@[GdL^D^]**	272.7 ± 2.2	0.22 ± 0.02	509.8 ± 86.0	0.22 ± 0.02	−31.7 ± 1.7	−10.1 ± 6.4
**6**	**LNO@[EuL^D^]**	296.6 ± 3.6	0.19 ± 0.01	518.0 ± 29.0	0.41 ± 0.06	−23.1 ± 0.5	0.0 ± 0.3
**7**	**LNO@[YbL^D^]**	359.1 ± 13.2	0.23 ± 0.02	675.6 ± 82.1	0.50 ± 0.31	−22.1 ± 1.2	3.5 ± 0.2
**8**	**LNO@[TbL^D^]**	176.3 ± 11.9	0.16 ± 0.03	864.6 ± 29.0	0.30 ± 0.06	−26.9 ± 2.8	8.1 ± 0.2

*N.B.* DLS data for **LNO@[TbL^A^]** are not reported due to sample background fluorescence which interfered with the measurement (Figure S4b). ^[a]^ Number mean value.

**Table 2:** Lifetimes of **[LnL^X^]** and **LNO@[LnL^X^]** (X = A, D) in solid state.

Entry	Sample	τ_1_/μs	τ_2_/μs
**1**	**[EuL^A^]**	171.6 ± 2.2	-
**2**	**LNO@[EuL^A^]**	84.3 ± 2.3	14.4 ± 1.2
**3**	**[EuL^D^]**	293.5 ± 4.5	-
**4**	**LNO@[EuL^D^]**	119.9 ± 3.4	51.1 ± 3.3
**5**	**[TbL^A^]**	243.6 ± 1.2	56.2 ± 2.3
**6**	**LNO@[TbL^A^]**	81.9 ± 0.5	-
**7**	**[TbL^D^]**	503.3 ± 10.6	77.3 ± 6.3
**8**	**LNO@[TbL^D^]**	257.1 ± 1.4	56.2 ± 2.2

## Data Availability

The original data presented in the study are openly available in Zenodo at https://doi.org/10.5281/zenodo.19693995.

## Supplementary Materials

The following supporting information can be downloaded at: https://www.mdpi.com/article/10.3390/nano16100591/s1, Synthetic procedures and characterizations: Mass spectra, NMR spectra and IR spectra; Bare LNO characterization (XRD and TEM); DLS characterization of LNO precursors; Comparative characterization of [Ln]-conjugated LNOs via CuAAC and SPAAC; Detailed parameters for T1 and T2 signal intensity fitting; SHG and PL maps overlaid to the bright field optical image of **LNO@[EuL^A^]** HNPs; Lifetimes of **[LnL^A^]** and **LNO@[LnL^A^]** in DMSO or in H_2_O (conc: 4 mM); Normalized, corrected emission and excitation spectra of **[GdL^A^]** and **LNO@[GdL^A^]** recorded in the solid state at room temperature; Normalized, corrected emission and excitation spectra of the **[LnL^A^]** complexes and the corresponding nanoparticle systems **LNO@[LnL^A^]** recorded in DMSO solutions (4 mM) at room temperature; Normalized, corrected emission spectra of the **[EuL^A^]** and **LNO@[EuL^A^]** recorded in the solid state at room temperature, upon excitation at 395 nm to populate the ^5^L_6_ excited level of trivalent europium from the ground ^7^F_0_ state; Emission- and excitation-corrected spectra of the **LNO@[GdL^D^]** and **LNO@[Gd→TbL^D^]** in H_2_O at room temperature.

## Abbreviations

The following abbreviations are used in this manuscript:

AcOH	Acetic acid
Alys	Azidolysine
CA	Contrast agent
CT	Computed tomography
CuAAC	Copper-catalyzed azide–alkyne [3+2]-cycloaddition
DCC	N,N′-Dicyclohexylcarbodiimide
DCE	Dichloroethane
DCM	Dichloromethane
DDQ	2,3-dichloro-5,6-dicyano-1,4-benzoquinone
D_H_	Hydrodynamic diameter
DIBO	Dibenzocyclooctyne
DIBO-NH_2_	Amino-modified dibenzocyclooctyne
DIPEA	Diisopropylethylamine
DLS	Dynamic light scattering
DMAc	N,N-Dimethylacetamide
DMF	N,N-Dimethylformamide
DMSO	Dimethylsulfoxide
EDX	Energy-dispersive X-ray spectroscopy
FCC	Flash column chromatography
FHG	Fourth harmonic generation
FTIR	Fourier transform infrared
IR	Infrared
Hex	Hexane
HNP	Harmonic nanoparticle
Ln	Lanthanide
LNO	Lithium niobate
MR	Magnetic resonance
MRI	Magnetic resonance imaging
MWCO	Molecular weight cut-off
NHS	N-Hydroxy succinimide
NIR	Near-infrared
NP	Nanoparticle
NPCF	Nitrophenyl chloroformate
PBS	Phosphate buffer saline
PDI	Polydispersity index
PEG	Poly(ethylene glycol)
PET	Positron emission tomography
PL	Photoluminescence
PMBBr	p-methoxybenzylbromide
Py	Pyridine
rt	Room temperature
SHG	Second-harmonic generation
SPAAC	Strain-promoted azide–alkyne [3+2]-cycloaddition
SPECT	Single-photon emission computed tomography
STEM	Scanning transmission electron microscopy
TACNB	1,4-bipicolinate-1,4,7-triazacyclononane
Talys	Tri-azidolysine peptide
TEA	Triethylamine
THF	Tetrahydrofuran
THG	Third-harmonic generation
TLC	Thin-layer chromatography
Tol	Toluene
UV	Ultraviolet
Vis	Visible
ZP	Zeta potential
