# Supplementary material for: Click Chemistry Functionalization of Harmonic Nanoparticles with Lanthanide Complexes Towards Tunable Platforms for Multimodal Imaging
Source: Nanomaterials (Basel). 2026 May 12;16(10):591. doi: 10.3390/nano16100591 (PMC13209949; doi:10.3390/nano16100591)
Supplement: Supplementary file 1 [file nanomaterials-16-00591-s001.zip › nanomaterials-4307452-supplementary.pdf]

Supporting Information

Click Chemistry Functionalization of Harmonic Nanoparticles with Lanthanide Complexes towards Tunable Platforms for Multimodal Imaging

Simon Dumolard [1], Volodymyr Multian [2,3], Adrian Gheata [1], Alessandra Spada [1], Katarzyna Pierzchala [4,5], Bernard Lanz [4,5], Ameni Dhouib [6], Yannick Mugnier [6], Jérémie Teyssier [3], Luigi Bonacina [2], Anne-Sophie Chauvin [7,*] and Sandrine Gerber-Lemaire [1,*]

Table of Content

1. Materials and Methods 3

2. Synthesis procedures and characterizations 4

1.1 Compound 1 4

1.2 Compound 2 6

1.3 Compound 3 8

1.4 Compound 4 10

1.5 Compound 5 12

1.6 Compound 6 13

1.7 H_3L^D ligand 15

2 NP surface modifications 20

2.1 Bare LNO HNP synthesis 20

2.2 Coated LNO intermediates synthesis 21

2.3 LNO@[LnL^A] and LNO@[LnL^D] characterization 22

3 Imaging experiments 27

3.1 MRI phantom imaging 27

3.2 Nonlinear optical microscopy & Ln luminescence via LNO SHG 27

3.3 Photophysical properties 29

1. Materials and Methods

Reagents and solvents were purchased from commercial sources (Aldrich, Acros, Merck, Fluka and VWR international) and preserved under argon. More sensitive compounds were stored in a desiccator if required. Reagents were used without further purification unless otherwise noted. All the reactions were performed in flame dried glassware under an inert atmosphere of argon when necessary. Evaporation and concentration under reduced pressure were conducted using Rotavapor devices from Büchi. All products were dried under medium vacuum (10^{-2} Bar) before analytic characterization. All dialysis purifications mentioned were performed against distilled H_2O or small alcohol mixtures when required at room temperature (dialysate changed every 3 h), with dialysis membranes from Roth (dialysis tubes Float-A-Lyzer G2 CE, 10 mL, exclusion limit 100-500 Da or 500-1'000 Da, dry packed treated with glycerine; unless otherwise mentioned). Samples were lyophilised in a VaCo 5 Zirbus technology freeze dryer (0.3 mbar, -80 °C).

Thin Layer Chromatography (TLC): Reactions were monitored using Merck Kieselgel 60F254 aluminium or glass backed plates. TLCs were visualized by UV fluorescence (254 nm) then one of the following reagent: $KMnO_4$, molybdenate, ninhydrine, pancaldi, p-anisaldehyde, vanillin.

Flash Column Chromatography (FCC): SiO_2 60A (230-400 mesh, particle size 40-63 µm) and Al_2O_3 60A (50-200 mesh, basic Brockmann I) were obtained from Fluka.

^{1}H-NMR and ^{13}C-NMR spectra: NMR spectra were recorded on a Bruker Avance III-400, Bruker Avance-400 or Bruker DRX-400 spectrometer at room temperature, ^{1}H frequency is at 400.13 MHz, ^{13}C frequency is at 100.62 MHz. Chemical shifts (δ) were reported in parts per million (ppm) relative to residual solvent peaks rounded to the nearest 0.01 ppm for proton and 0.1 ppm for carbon (ref: $CHCl_3$ [^{1}H: 7.26 ppm, ^{13}C: 77.2 ppm], MeOH [^{1}H: 3.31 ppm, ^{13}C 49.0 ppm], ACN [^{1}H: 1.94 ppm, ^{13}C: 1.3 ppm], DMSO [^{1}H: 2.50 ppm, ^{13}C: 39.5 ppm]). Coupling constants (J) were reported in Hz to the nearest 0.1 Hz. Peak multiplicity was indicated as follows s (singlet), d (doublet), t (triplet), q (quartet), quin (quintet), sex (sextet), m (multiplet) and br (broad). Attribution of peaks was done using the multiplicities and integrals of the peaks. The attributed peaks are described with an * to indicate a stereocenter and the letter in italics corresponds to the described atom.

Accurate Mass: The qualitative accurate masses were measured by ESI-TOF using the Xevo G2-S QTOF (Waters) and nanoESI-FT-MS using the Elite™ Hybrid Ion Trap-Orbitrap (ThermoFisher) Mass Spectrometer.

FT-IR: Spectra acquired on a Nicolet 6700 from ThermoFisher Scientific and on a Spectrum 3 from PerkinElmer using KBr pellet.

Dynamic Light Scattering (DLS) and Zeta potential: measurements made with Malvern NanoZ instrument. All reported hydrodynamic diameters are expressed in number mean values. Size distribution plots show the intensity size data. Correlograms were used to aid identification of sedimentation and aggregation processes. Each value is expresses as mean value of at least 3 replicates with corresponding standard deviation.

Sonicator: Elmasonic Branson 1800 Ultrasonic Cleaner sonicator.

Centrifuge: Beckman Coulter Allegra X-30 centrifuge.

STEM-EDX: Scanning transmission electron microscopy (STEM) and Energy-dispersive X-ray spectroscopy (EDX) were performed at the Interdisciplinary Centre for Electron Microscopy (CIME, EPFL, Lausanne, Switzerland). High-angle annular dark field (HAADF) STEM images were acquired on a FEI Tecnai Osiris at 200 kV using the following parameters: probe 0.6-1 nA, CL 115 mm. EDX spectroscopy elemental maps were acquired on a FEI Tecnai Osiris using a super-X detector. The samples were prepared by drop-casting a colloidal suspension of NPs on thin amorphous carbon films or on Lacey TEM grids (Electron Microscopy Sciences).

2. Synthesis procedures and characterizations

1.1 Compound 1

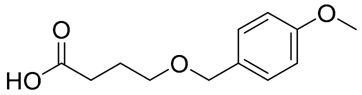

MS (ESI-Quad) m/z: $[M-H]^-$ calcd for $C_{12}H_{16}O_4^-$ 223.0976; Found 223.0975.

^{1}H-NMR (400 MHz, Chloroform-*d*) δ 7.25 (d, 3H), 6.88 (d, 2H), 4.44 (s, 2H), 3.80 (s, 3H), 3.50 (t, *J* = 6.1 Hz, 2H), 2.47 (t, *J* = 7.3 Hz, 2H), 1.93 (p, *J* = 6.1 Hz, 2H).

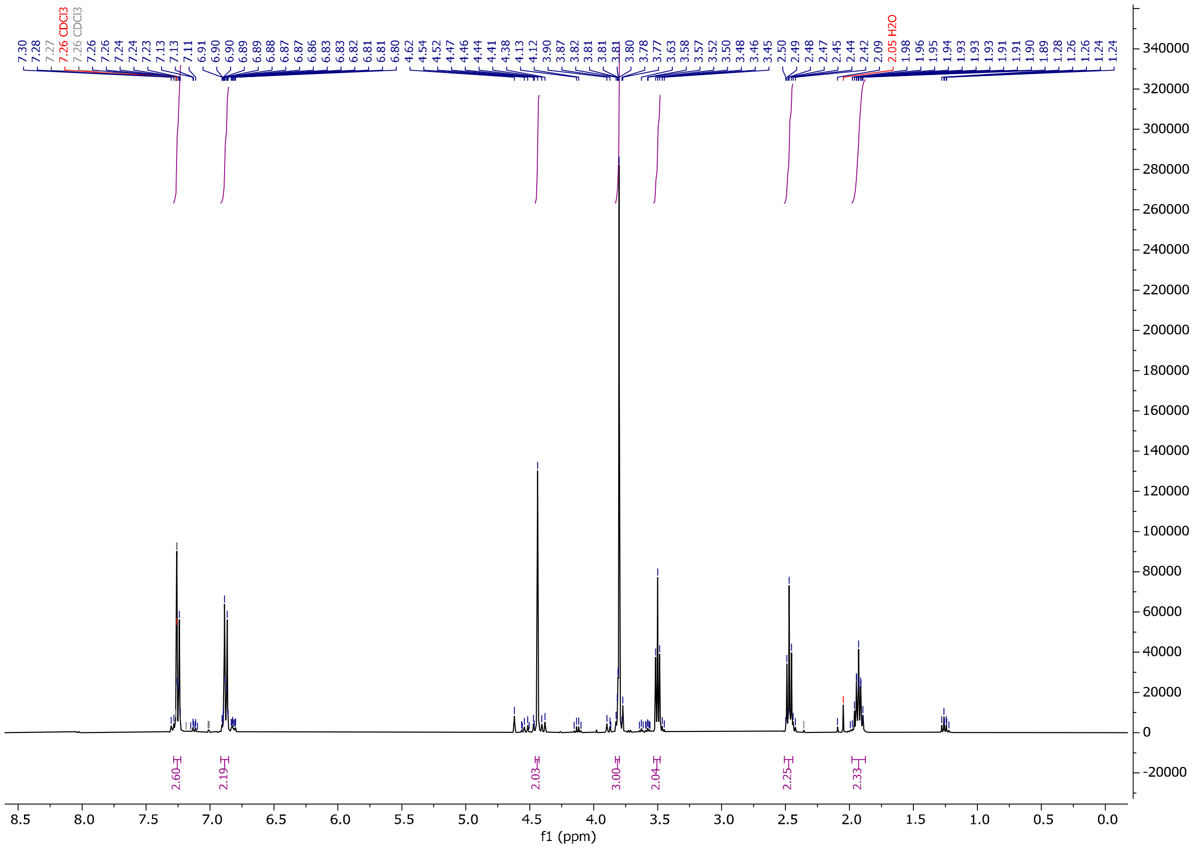

Figure S1. ^{1}H-NMR spectrum of compound **1**.

1.2 Compound 2

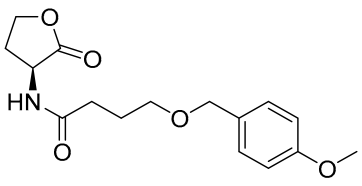

MS (ESI-Quad) m/z: $[M+H]^+$ calcd for $C_{16}H_{22}NO_5^+$ 308.1492; found 307.8016, and $[M+Na]^+$ calcd for $C_{16}H_{21}NNaO_5^+$ 330.1312; found 330.1271.

^{1}H-NMR (400 MHz, Chloroform-*d*) δ 7.28 – 7.23 (m, 2H, Ar-*H*), 6.91 – 6.86 (m, 2H, Ar-*H*), 6.40 (s, 1H, N*H*), 4.60 – 4.15 (m, 5H, C*H-CH2-C*H2* and O-C*H2*-Ar and C**H*), 3.80 (s, 3H, O-C*H3*), 3.51 (t, *J* = 5.8 Hz, 2H, CH2-C*H2*-O), 2.76 – 2.66 (m, 1H, C*H-C*H2*-CH2), 2.38 (t, *J* = 7.0 Hz, 2H, CO-C*H2*-CH2), 1.98 – 1.90 (m, 2H. CH2-C*H2*-CH2), 1.90 – 1.83 (m, 1H, C*H-C*H2*-CH2).

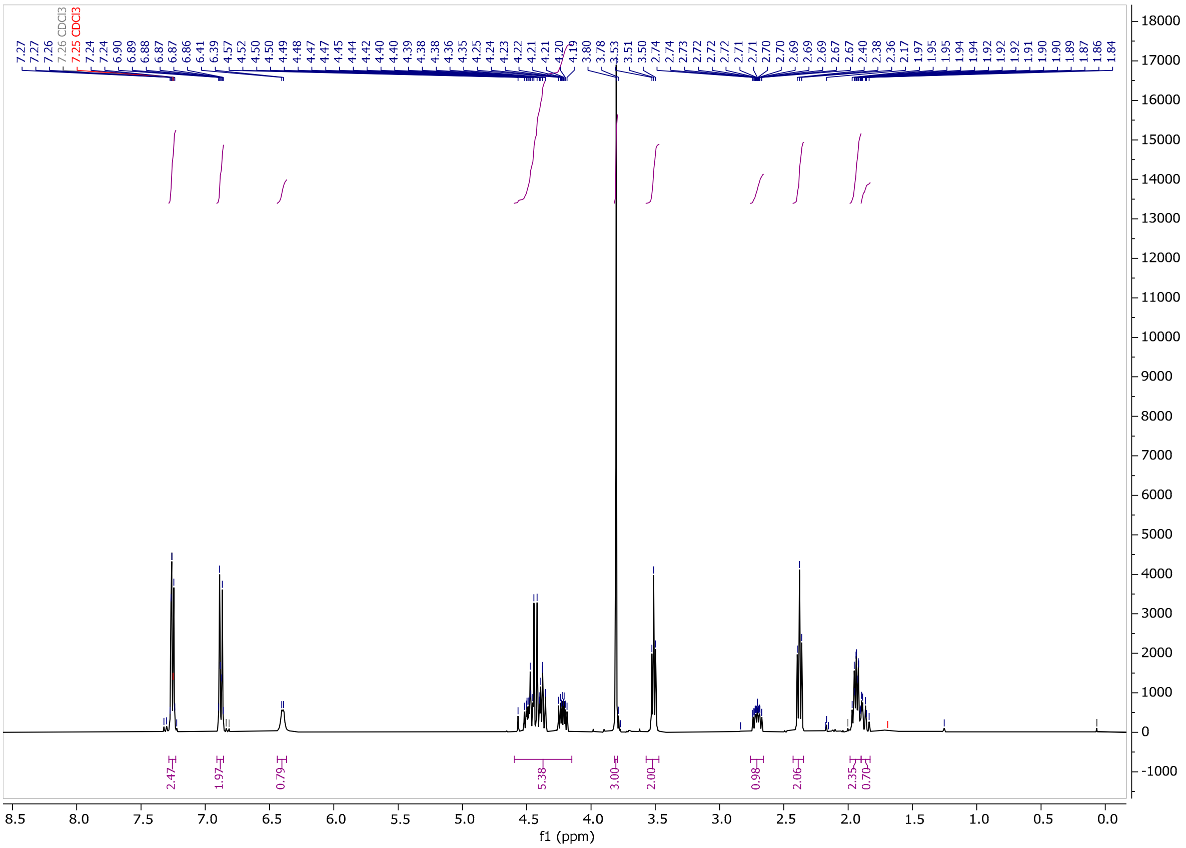

Figure S2. ^{1}H-NMR spectrum of compound **2**.

^{13}C-NMR (101 MHz, Chloroform-d): δ 175.30 (*C*OO-C*H), 173.45 (*C*ONH), 159.30 (*C*-OCH3), 130.25 (O-CH2-*C*), 129.57 (2x *C*H-C-OCH3), 113.87 (2x O-CH2-C-*C*H), 72.71 (O-*C*H2-C), 69.00 (CH2-*C*H2-O), 65.92 (C*H-CH2-*C*H2), 55.30 (O-*C*H3), 49.04 (COO-*C**H), 33.41 (*C*H2-CH2-CH2), 30.25 (C*H-*C*H2-CH2), 25.40 (CH2-*C*H2-CH2).

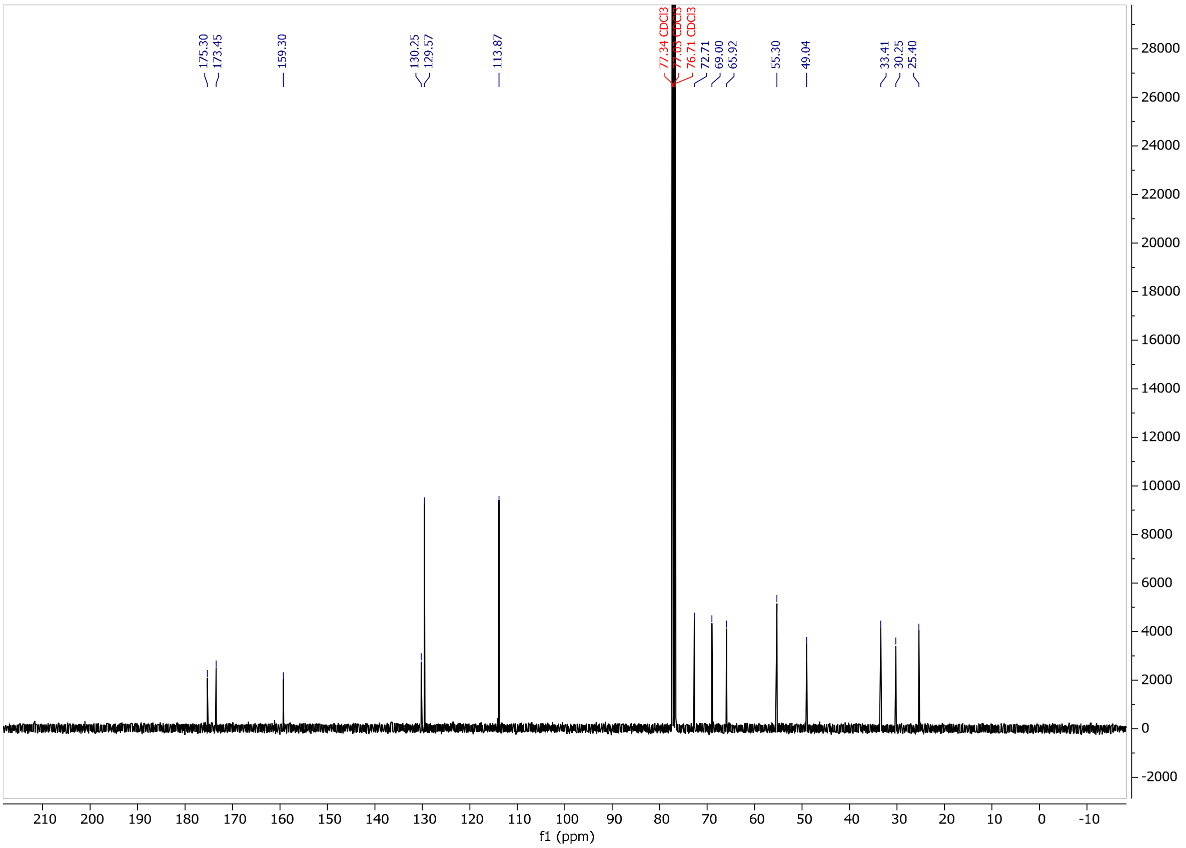

Figure S3. ^{13}C-NMR spectrum of compound **2**.

1.3 Compound 3

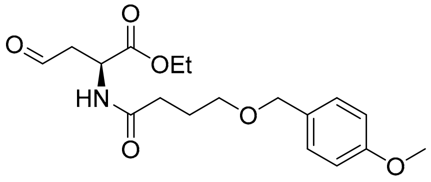

MS (ESI-Quad) m/z: $[M+H]^+$ calcd for $C_{18}H_{26}NO_6^+$ 352.1755; found 351.9747, and $[M+Na]^+$ calcd for $C_{18}H_{25}NNaO_6^+$ 374.1574; found 374.2094.

^{1}H-NMR (400 MHz, Chloroform-*d*) δ 9.67 (s, 1H, C*HO*), 7.29 – 7.22 (m, 2H, Ar-*H*), 6.91 – 6.84 (m, 2H, Ar-*H*), 6.56 (d, *J* = 7.7 Hz, 1H, N*H*), 4.85 – 4.78 (m, 1H, C*-*H*), 4.42 (s, 2H, O-C*H2*-Ar), 4.20 (q, *J* = 7.1 Hz, 2H, O-C*H2*-CH3), 3.80 (s, 3H, O-C*H3*), 3.47 (td, *J* = 6.1, 2.3 Hz, 2H, CH2-C*H2*-O), 3.10 – 2.94 (m, 2H, CHO-C*H2*-C*H), 2.33 (td, *J* = 7.2, 3.0 Hz, 2H, CO-C*H2*-CH2), 1.91 (q, 2H, CH2-C*H2*-CH2), 1.25 (t, *J* = 7.1 Hz, 3H, O-CH2-C*H3*).

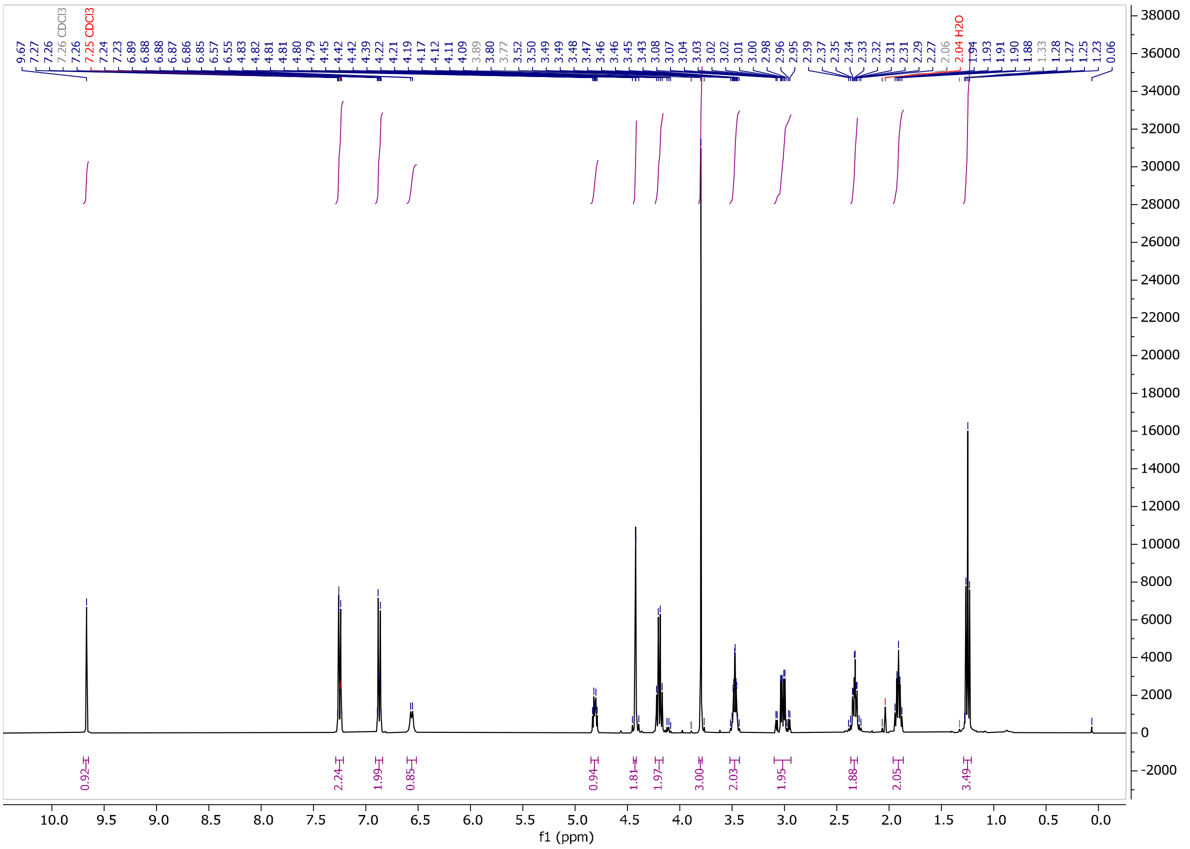

Figure S4. ^{1}H-NMR spectrum of compound **3**.

^{13}C-NMR (101 MHz, Chloroform-d): δ 199.29 (*C*HO), 172.64 (*C*ONH), 170.67 (*C*OO), 159.22 (*C*-OCH3), 130.41 (O-CH2-*C*), 129.38 (2x *C*H-C-OCH3), 113.81 (2x O-CH2-C-*C*H), 72.61 (O-*C*H2-C), 68.92 (CH2-*C*H2-O), 61.97 (O-*C*H2-CH3), 55.29 (O-*C*H3), 47.32 (COO-*C**H), 45.69 (C*H-*C*H2-CHO), 33.29 (*C*H2-CH2-CH2), 25.50 (CH2-*C*H2-CH2), 14.06 (O-CH2-*C*H3).

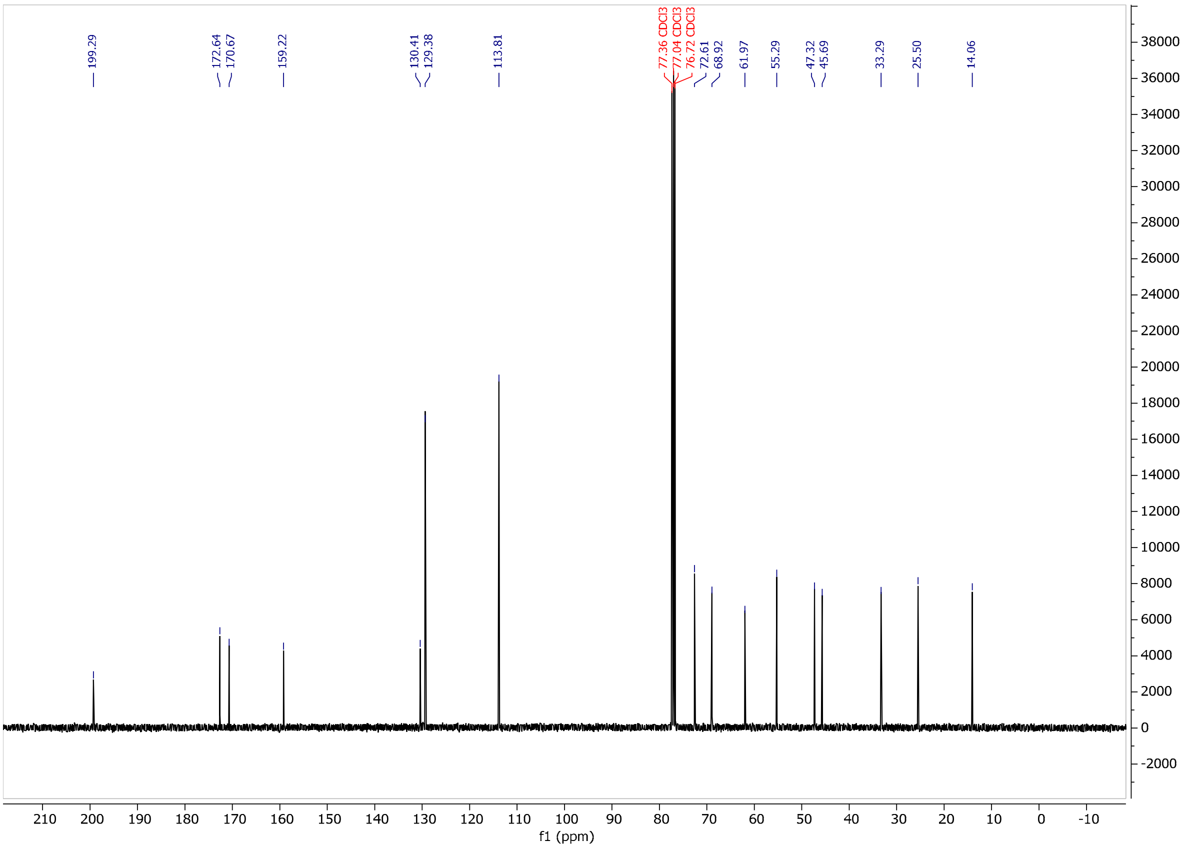

Figure S5. ^{13}C-NMR spectrum of compound **3**.

1.4 Compound 4

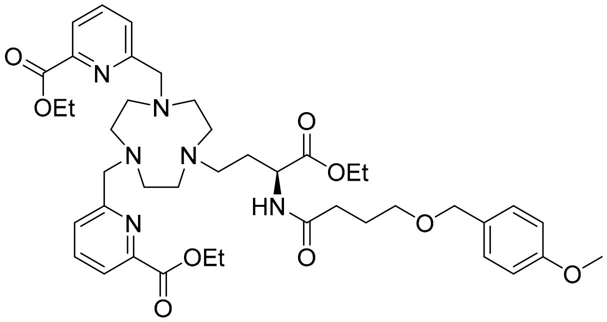

HRMS (ESI/QTOF) m/z: $[M + H]^+$ Calcd for $C_{42}H_{59}N_6O_9^+$ 791.4338; Found 791.4356.

^{1}H-NMR (400 MHz, Acetonitrile-d_3) δ 7.95 – 7.90 (m, 2H, Py-*H*), 7.88 – 7.81 (m, 2H, Py-*H*), 7.76 – 7.70 (m, 2H, Py-*H*), 7.25 – 7.18 (m, 3H, Ar-*H*), 6.89 – 6.83 (m, 2H, Ar-*H*), 4.48 – 4.41 (m, 1H, C*-*H*), 4.41 – 4.31 (m, 6H, O-C*H2*-CH3), 4.09 (m, 2H, CH2-C*H2*-C*H), 3.89 (s, 2H, O-C*H2*-Ar), 3.83 (s, 4H, Py-C*H2*-N), 3.75 (s, 3H, O-C*H3*), 3.42 (t, *J* = 6.3 Hz, 2H, CH2-C*H2*-O), 2.92 – 2.65 (m, 12H, N-C*H2*-C*H2*-N), 2.55 – 2.47 (br, 2H, N-C*H2*-CH2-C*), 2.22 (t, *J* = 7.3 Hz, 2H, CO-C*H2*-CH2), 1.84 – 1.76 (m, 2H, CH2-C*H2*-CH2), 1.35 (t, *J* = 7.1 Hz, 6H, O-CH2-C*H3*), 1.19 (t, *J* = 7.1 Hz, 3H, O-CH2-C*H3*).

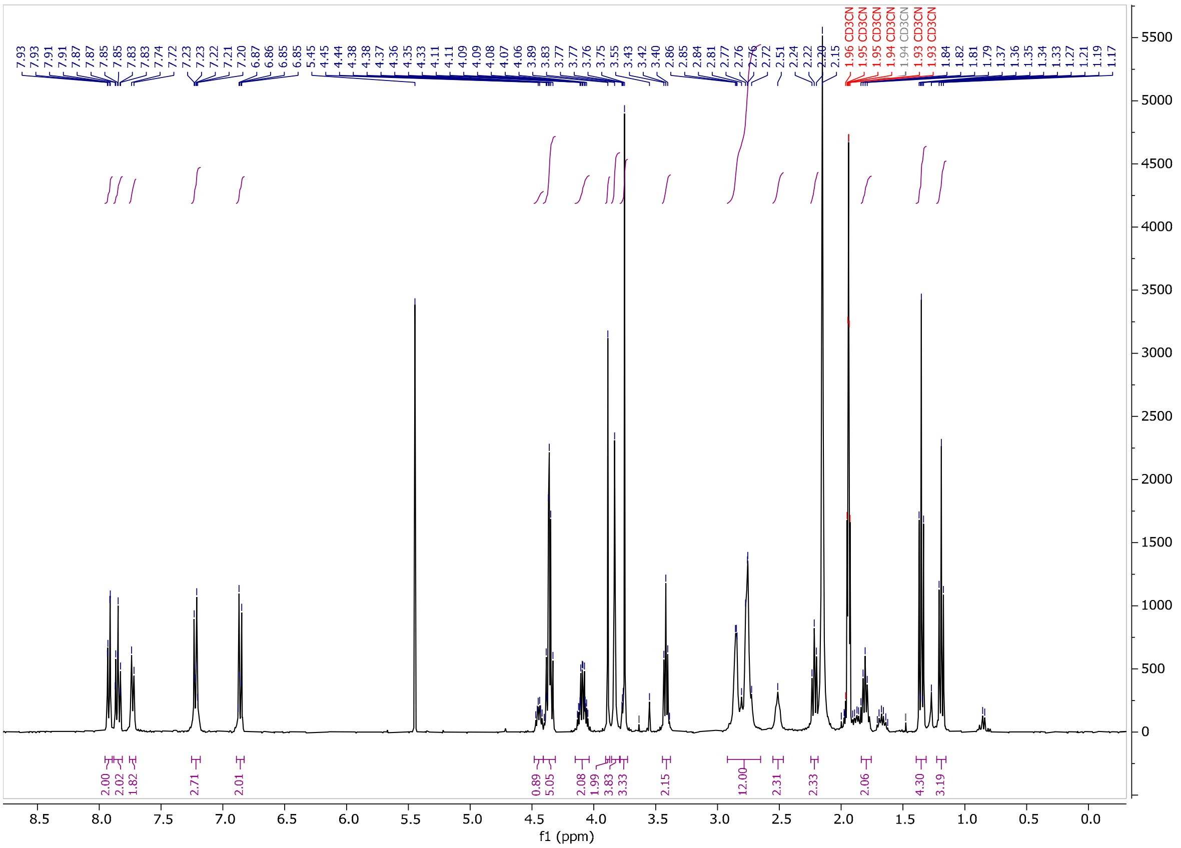

Figure S6. ^{1}H-NMR spectrum of compound **4**.

^{13}C-NMR (101 MHz, CD_3CN) δ 173.47 (*C*ONH), 166.73 (*C*OO), 166.23 (*C*OO), 160.12 (*C*-O-CH3), 148.53 (*C*-Py), 138.36 (*C*-Py), 131.94 (O-CH2-*C*), 130.20 (2x *C*H-C-OCH3), 127.59 (*C*-Py), 127.46 (*C*-Py), 124.14 (*C*-Py), 114.58 (2x O-CH2-C-*C*H), 72.98 (O-*C*H2-CH3), 70.01 (CH2-*C*H2-O), 62.29 (O-*C*H2-CH3), 61.68 (CH2-*C*H2-C*H), 55.85 (O-*C*H3), 55.33 (N-*C*H2-CH2-C*H), 53.01 (O-*C*H2-Ar), 33.29 (CO-*C*H2-CH2), 26.55 (CH2-*C*H2-CH2), 14.59 (O-CH2-*C*H3), 14.55 (O-CH2-*C*H3).

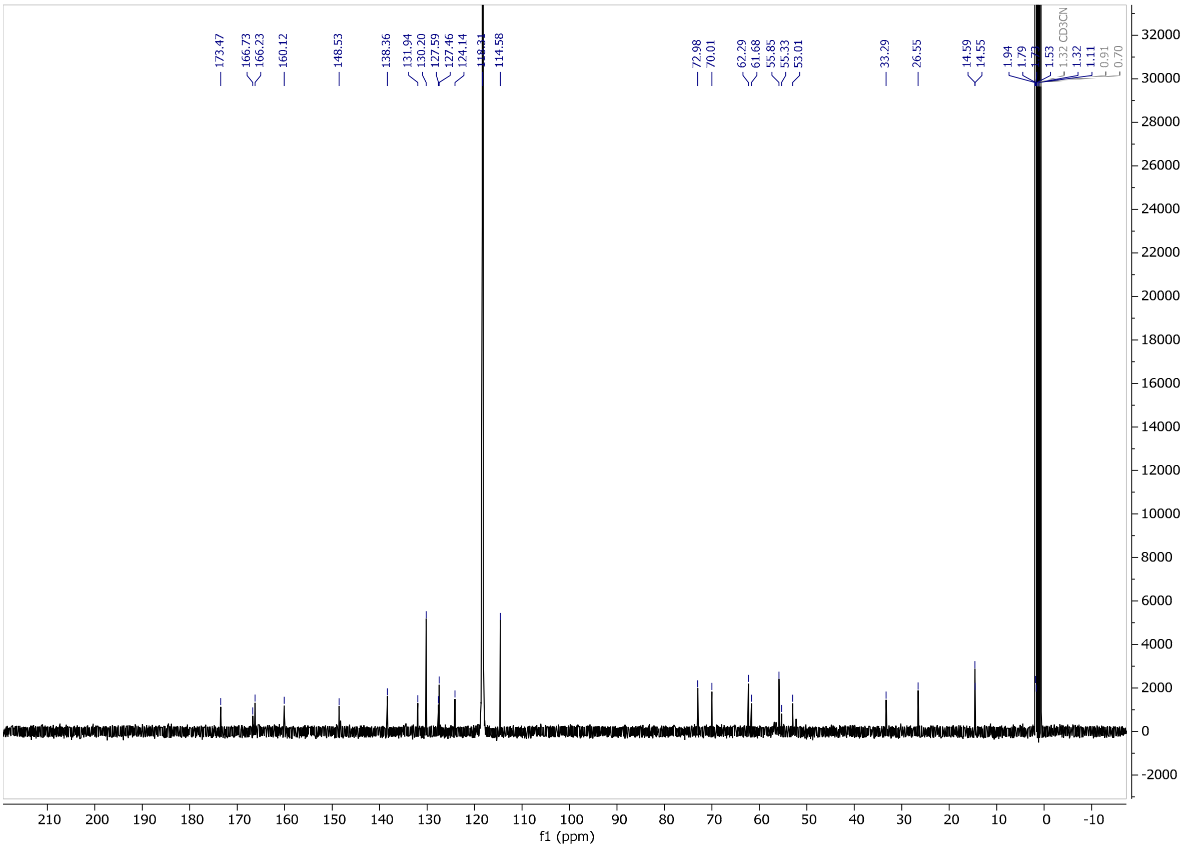

Figure S7. ^{13}C-NMR spectrum of compound **4**.

1.5 Compound 5

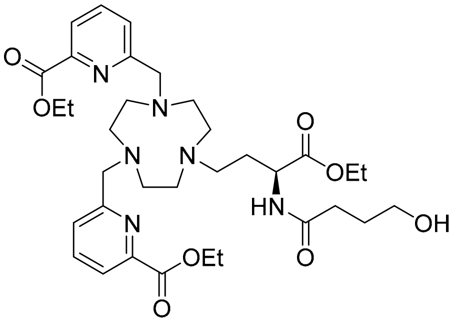

HRMS (nanochip-ESI/LTQ-Orbitrap) m/z: $[M + H]^+$ Calcd for $C_{34}H_{51}N_6O_8^+$ 671.3763; Found 671.3746.

^{1}H-NMR (400 MHz, Acetonitrile-*d*$_3$) δ 7.97 – 7.91 (m, 2H, Py-*H*), 7.90 – 7.83 (m, 2H, Py-*H*), 7.79 – 7.67 (m, 2H, Py-*H*), 7.28 – 7.13 (br, 1H, N*H*), 4.50 – 4.42 (m, 1H, C*-*H*), 4.36 (q, *J* = 7.1 Hz, 4H, O-C*H2*-CH3), 4.16 – 4.03 (m, 2H, CH2-C*H2*-C*H), 3.88 – 3.83 (m, 4H, Py-C*H2*-N), 3.49 (t, *J* = 6.2 Hz, 2H, CH2-C*H2*-O), 2.97 – 2.68 (m, 12H, N-C*H2*-C*H2*-N), 2.69 – 2.43 (m, 2H, N-C*H2*-CH2-C*), 2.23 (t, *J* = 6.8 Hz, 2H, CO-C*H2*-CH2), 1.72 (p, *J* = 6.8 Hz, 2H, CH2-C*H2*-CH2), 1.41 – 1.31 (m, 6H, O-CH2-C*H3*), 1.19 (t, *J* = 7.1 Hz, 3H, O-CH2-C*H3*).

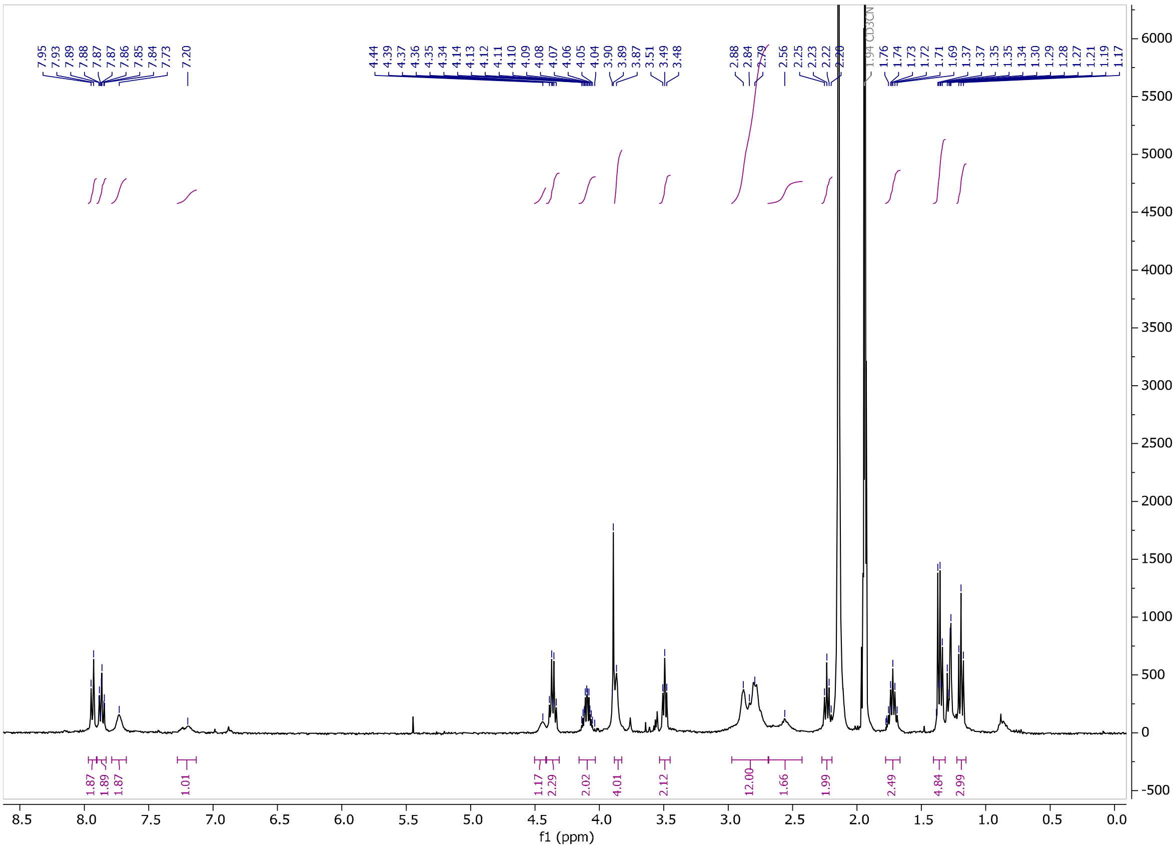

Figure S8. ^{1}H-NMR spectrum of compound **5**.

1.6 Compound 6

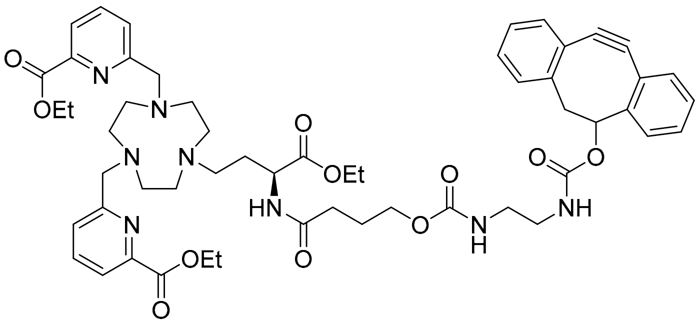

HRMS (ESI/QTOF) m/z: $[M + H]^+$ Calcd for $C_{5}4H_{67}N_{8}O_{11}^{+}$ 1003.4924; Found 1003.4896.

^{1}H-NMR (400 MHz, Acetonitrile-d_3) δ 7.94 – 7.89 (m, 2H, Py-*H*), 7.88 – 7.80 (m, 2H, Py-*H*), 7.74 – 7.69 (m, 2H, Py-*H*), 7.60 – 7.51 (m, 1H, DIBO-Ar*H*), 7.42 – 7.28 (m, 7H, DIBO-Ar*H*), 6.35 – 6.25 (m, 2H, N*H*), 5.86 – 5.81 (m, 1H, N*H*), 5.37 – 5.29 (m, 1H, DIBO-C*H*), 4.48 – 4.40 (m, 1H, C**H*), 4.35 (q, *J* = 7.1 Hz, 4H, O-C*H2*-CH3), 4.14 – 4.03 (m, 2H, CH2-C*H2*-C*H), 4.02 – 3.92 (m, 2H, CH2-C*H2*-OCON), 3.83 (s, 4H, Py-C*H2*-N), 3.24 – 3.08 (m, 5H, NH-C*H2*-C*H2*-NH and DIBO-C*H2*), 2.87 – 2.63 (m, 13H, N-C*H2*-C*H2*-N and DIBO-C*H2*'), 2.55 – 2.46 (m, 2H, N-C*H2*-CH2-C*), 2.14 – 2.09 (m, 2H, CO-C*H2*-CH2), 1.88 – 1.75 (m, 2H, CH2-C*H2*-CH2), 1.34 (t, *J* = 7.1 Hz, 6H, O-CH2-C*H3*), 1.18 (t, *J* = 7.0 Hz, 3H, O-CH2-C*H3*).

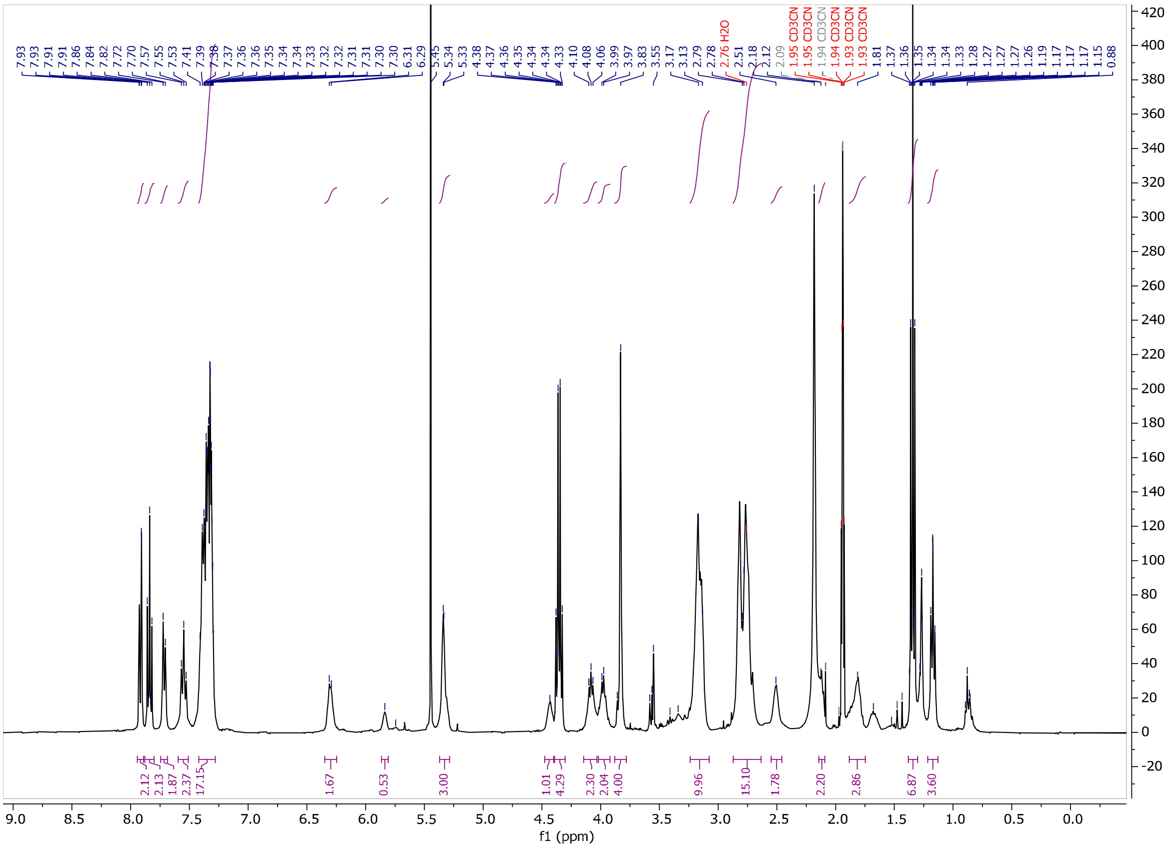

Figure S9. ^{1}H-NMR spectrum of compound **6**.

^{13}C-NMR (101 MHz, CD_3CN) δ 165.80, 153.06, 151.98, 148.07, 137.99, 130.75, 128.94, 128.87, 127.86, 127.83, 127.74, 127.07, 126.74, 126.45, 124.59, 123.96, 123.77, 121.42, 113.14, 110.34, 76.65, 64.24, 61.92, 61.35, 54.91, 46.55, 42.32, 41.36, 40.31, 32.51, 25.48, 14.18, 14.13.

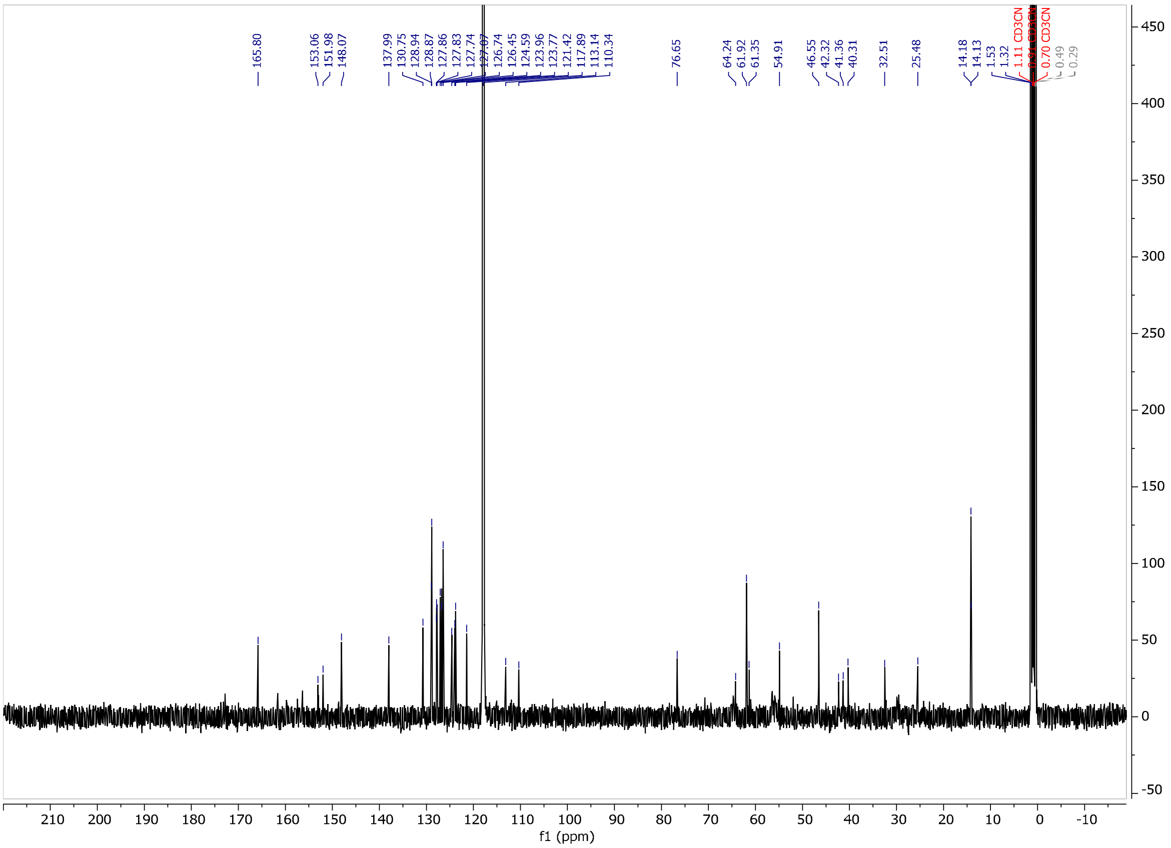

Figure S10. ^{13}C-NMR spectrum of compound **6**.

1.7 H_3L^D ligand

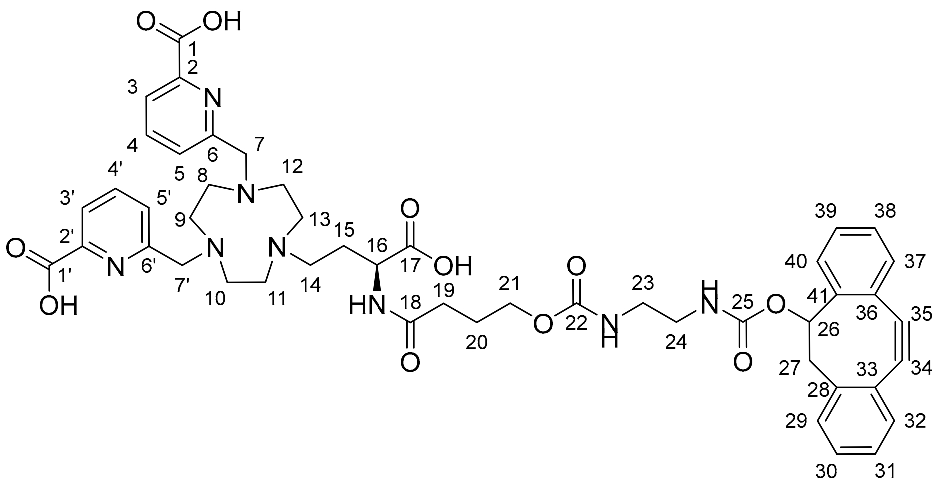

HRMS (ESI/QTOF) m/z: $[M + H]^+$ Calcd for $C_{48}H_{55}N_8O_{11}^+$ 919.3985; Found 919.4017.

IR (ATR after MeOH evaporation, cm−1): v = 3368 (br), 2956 (m), 2921 (m), 2852 (m), 2153 (w), 1697 (m), 1619 (s), 1586 (s), 1548 (m), 1461 (m), 1438 (m), 1388 (m), 1263 (m), 1150 (w), 1115 (w), 1084 (w), 1036 (w), 889 (w), 761 (m), 678 (w).

^{1}H-NMR (400 MHz) (400 MHz, Methanol-d_4) δ 8.02 – 7.95 (m, 2H, H3-3'), 7.91 – 7.82 (m, 2H, H4-4'), 7.61 – 7.53 (m, 1H, H-DIBO), 7.50 – 7.43 (m, 2H, H5-5'), 7.42 – 7.26 (m, 7H, H-DIBO), 5.44 – 5.37 (m, 1H, H26), 4.29 – 4.21 (m, 1H, H16), 4.15 – 3.93 (m, 6H, H7+H21), 3.26 – 2.94 (m, 13H, H8-9-10-11-12-13+H27), 2.83 – 2.75 (m, 1H, H27'), 2.75 – 2.63 (m, 4H, H23-24), 2.41 – 2.31 (m, 2H, H14), 1.94 – 1.79 (m, 4H, H15+H19), 1.36 – 1.32 (m, 2H, H20).

The remaining signals are attributed to impurities from the FC eluting solvents, *i.e.* DCM, alkanes and light alcohols, or to traces of non-hydrolyzed starting materials evidenced by ethyl esters signals. HPLC purification of advanced intermediates might help achieve higher purity but was not attempted due to the low product amounts available.

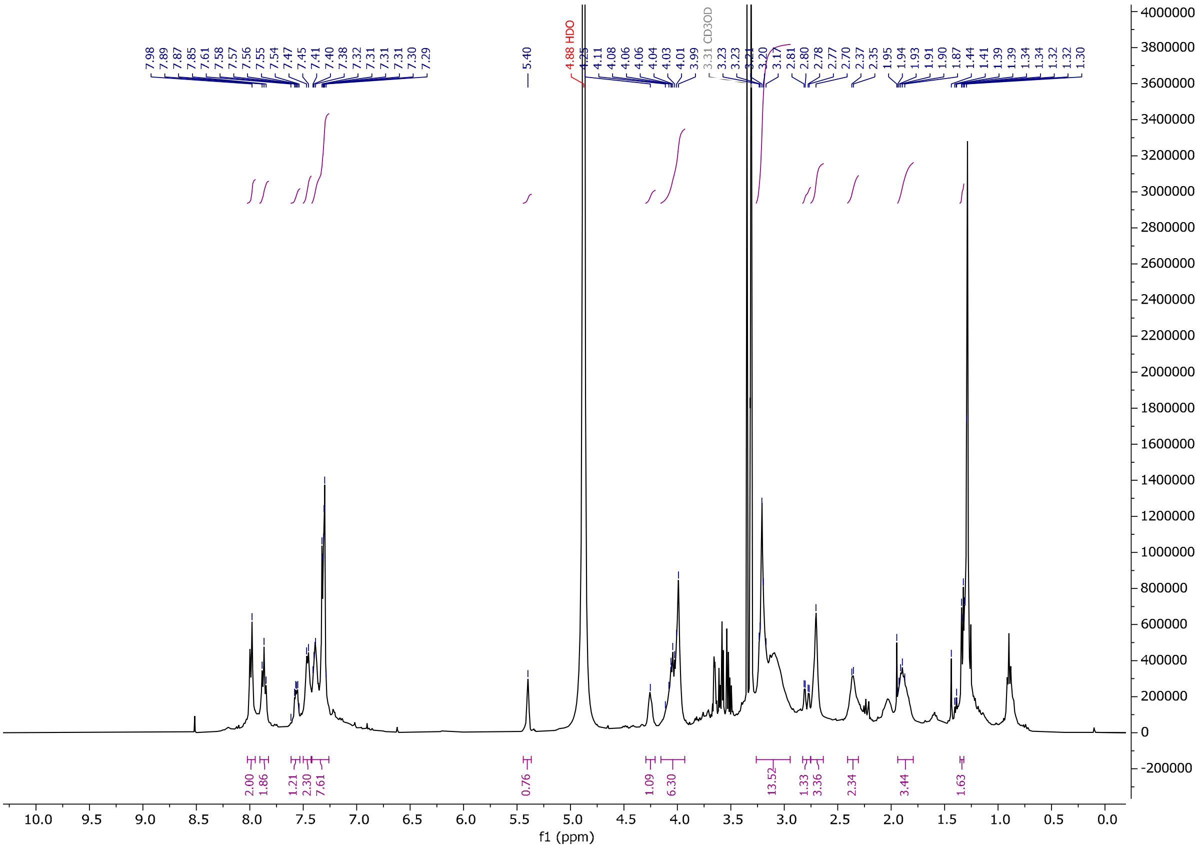

Figure S11. ^{1}H-NMR spectrum of $\mathbf{H_3L^D}$.

^{13}C-NMR (101 MHz, Methanol-*d*$_4$) δ 175.03 (C17), 172.17 (C3-3'), 158.34 (C18), 155.59 (C22), 153.60 (C-33 or C41), 152.49 (C25), 139.57 (C4-4'), 131.18 (C-DIBO), 129.35 (C-DIBO), 128.33 (C-DIBO), 128.26 (C-DIBO), 127.14 (C-DIBO), 126.49 (C5), 125.15 (C-DIBO), 124.28 (C3-3'), 122.33 (C28 or C33 or C36 or C41), 113.81 (C28 or C33 or C36 or C41), 110.98 (C28 or C33 or C36 or C41), 77.95 (C26), 73.84 (C34 or C35), 69.37 (C34 or C35), 65.09 (C21), 62.28 (C7), 55.49 (C-TACN), 53.72 (C16), 51.63 (C-TACN), 47.18 (C27), 41.79 (C-TACN), 41.65 (C-TACN), 33.06 (C14), 28.76(C15 or C19), 26.20 (C15 or C19), 23.72 (C20).

The two missing C-TACN, C23 and C24 presumably are hidden by the solvent peaks. The remaining signals chemical shifts are in agreement with the hypothesized structures, supporting the aforementioned impurities.

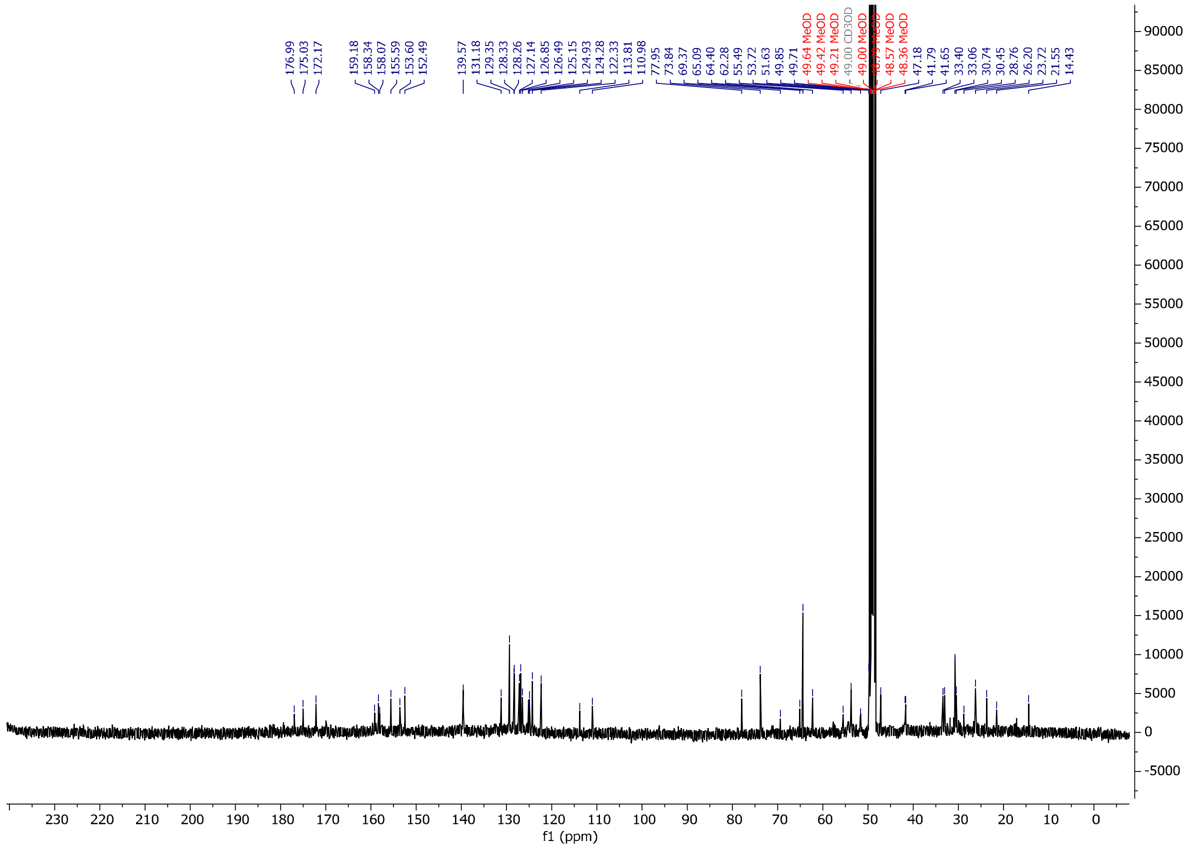

Figure S12. ^{13}C-NMR spectrum of **H_3L^D**.

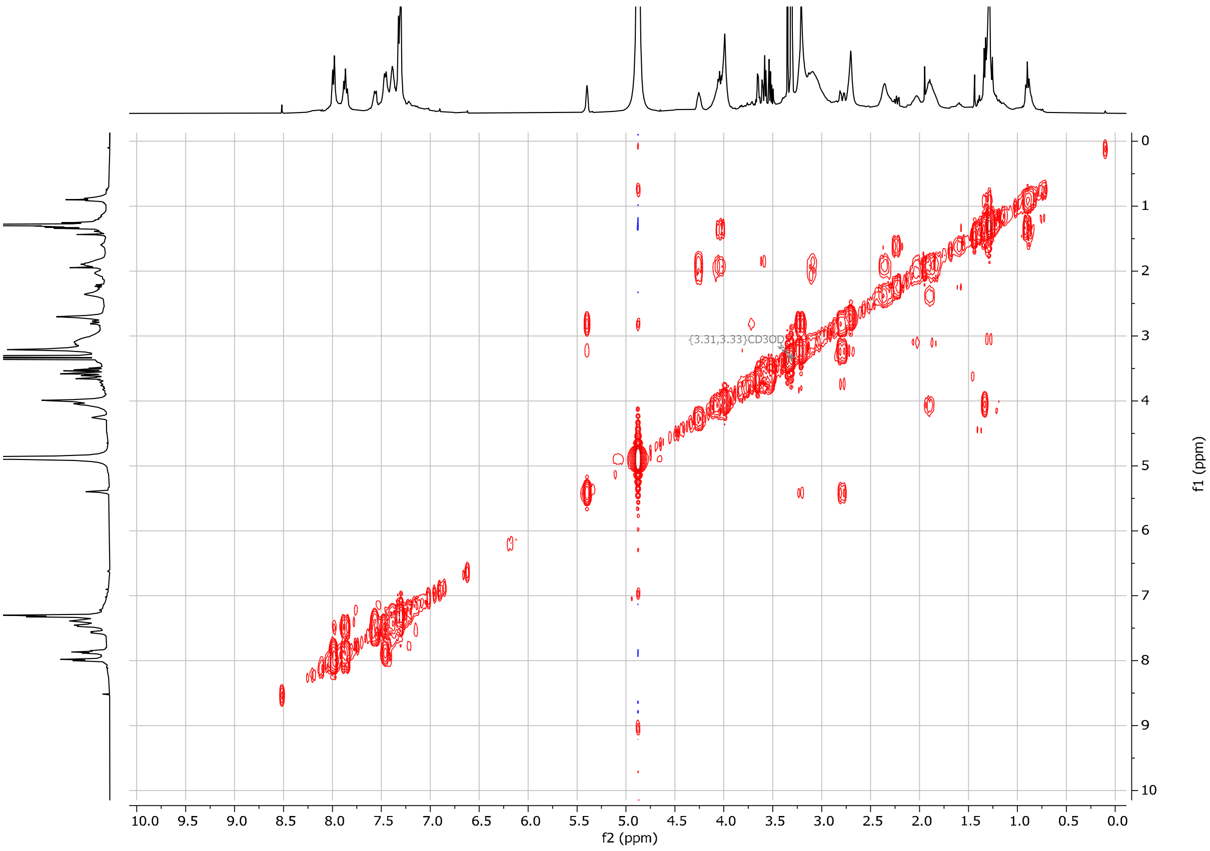

Figure S13. ^{1}H-^{1}H COSY NMR spectrum of $\mathbf{H_3L^D}$.

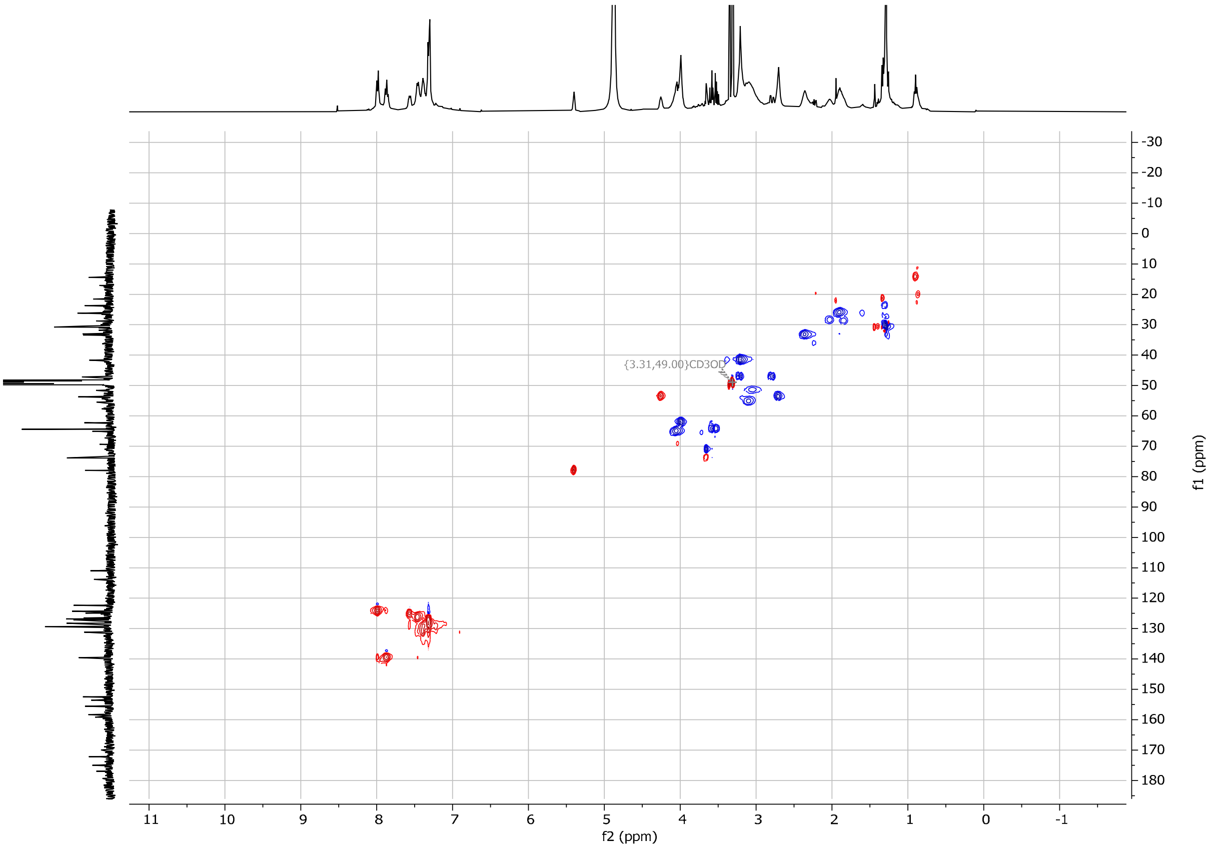

Figure S14. ^{1}H-^{13}C HSQC NMR spectrum of $\mathbf{H_3L^D}$.

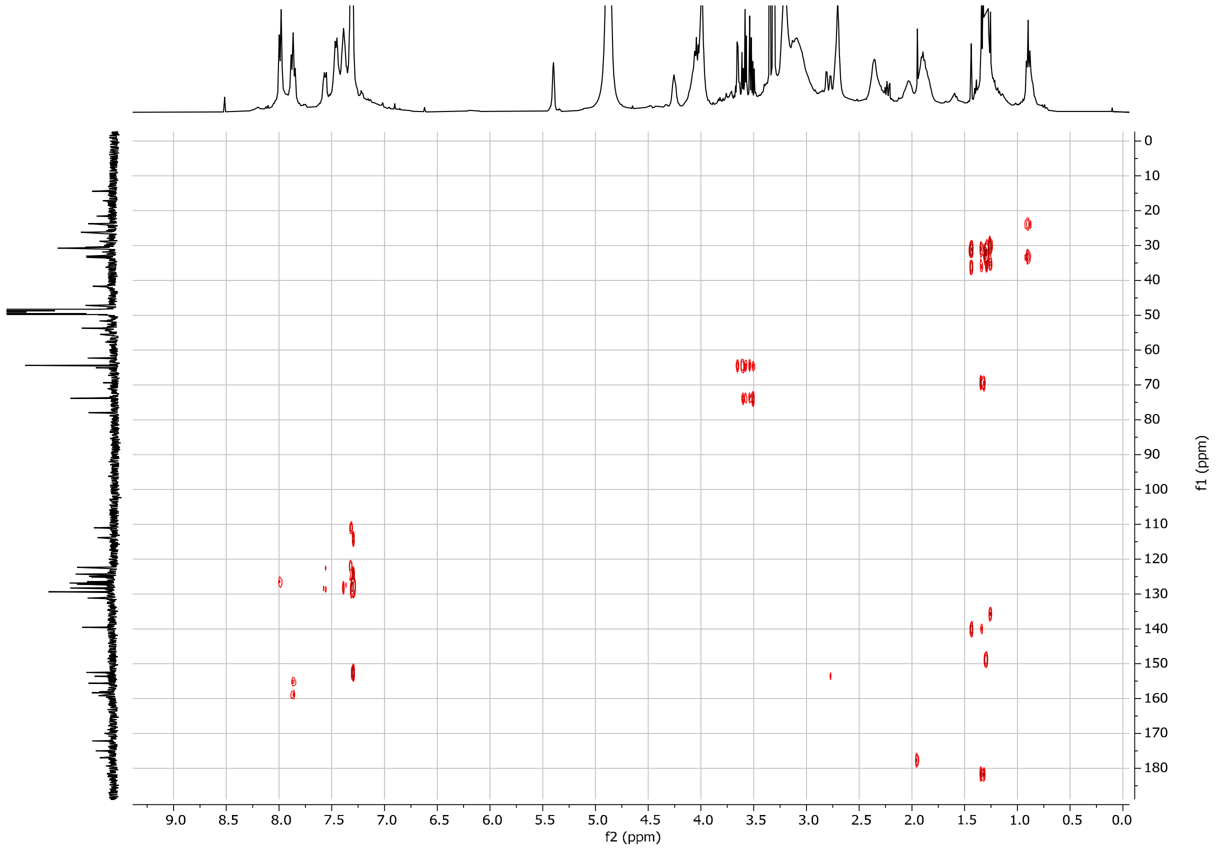

Figure S15. ^{1}H-^{13}C HMBC NMR spectrum of $\mathbf{H_3L^D}$.

2 NP surface modifications

Two batches of bare LNOs were used in this study, namely **S2** and **S95**. **Bare LNO S2** were used for MRI studies, and **bare LNO S95** were used for photophysical properties investigation.

2.1 Bare LNO HNP synthesis

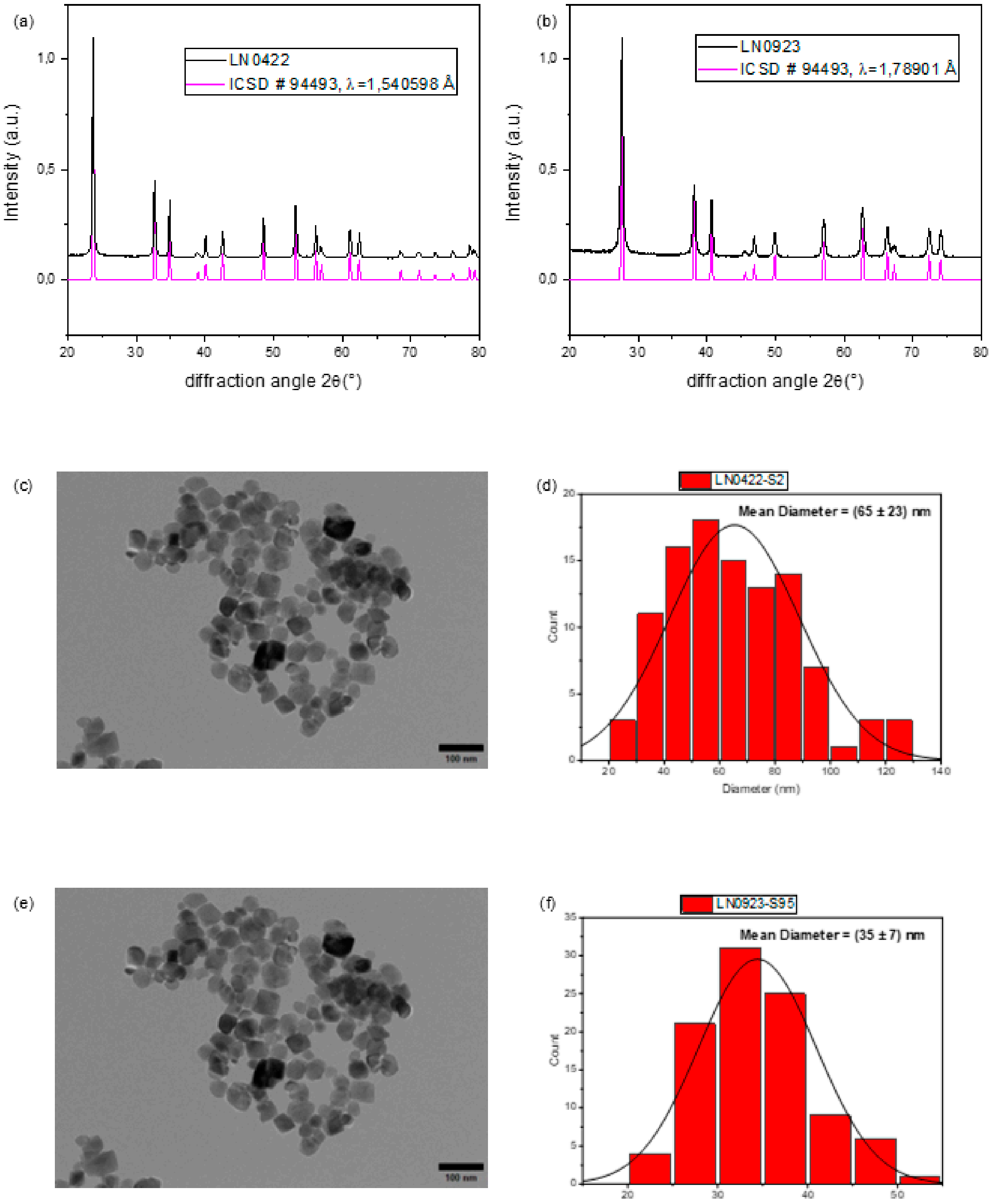

Figure S16. Bare LNO characterization. XRD patterns of the bare LNO (a) S2 and (b) S95 samples. From the XRD peak broadening of the different (hkl) reflections, the apparent nanocrystal size is estimated within the 31-47 nm range for S2, and within the 20-30 nm range for S95. TEM images (c, e) and size analysis over 100 nanocrystals (d, f) for LNO S2 and S95, respectively.

2.2 Coated LNO intermediates synthesis

The number average hydrodynamic diameter (D_H), PDI and ZP at each surface modification step is described in **Table S1**. Entries 1 to 3 in **Table S1** correspond to the preparation of **LNO@[GdL^D]** used in MRI studies; and entries 4 to 5 correspond to the **LNO@[LnL^A]** and **LNO@[LnL^D]** preparation for photoluminescence experiments. Despite variations in D_H between LNO batches, the trends across surface modification steps remained comparable to reported data.[1]

Table S1. DLS characterization of LNO precursors based on previously reported procedures.

Entry	Sample	Organic medium		Aqueous medium		Zeta potential (mV)	
		D_H (nm)	PDI	D_H (nm)	PDI	pH 7.4	pH 3
**1[a]**	**Bare LNO S2**	NA	NA	90.1 ± 3.3	0.10 ± 0.02	-25.4 ± 1.0	-28.1 ± 0.6
**2[a]**	**LNO@Si-COOH**	199.5 ± 11.8	0.14 ± 0.03	136.2 ± 5.3	0.08 ± 0.01	-47.7 ± 3.2	-8.45 ± 0.4
**3[a]**	**LNO@Si-Talys**	291.2 ± 8.7	0.21 ± 0.02	240.7 ± 45.5	0.28± 0.02	-42.2 ± 1.3	-16.1 ± 3.4
**4[b]**	**Bare LNO S95**	70.1 ± 4.2	0.18 ± 0.02	60.0 ± 4.3	0.20 ± 0.01	-36.4 ± 0.6	-14.7 ± 0.3
**5[b]**	**LNO@Si-COOH**	128.6 ± 19.1	0.19 ± 0.021	489.9 ± 194.9	0.24 ± 0.03	-33.8 ± 1.0	-18.5 ± 0.2
**6[a, c]**	**LNO@[Gd→TbL^D]**	506.5 ± 46.7	0.53 ± 0.07	805.4 ± 57.2	0.39 ± 0.06	-20.3 ± 2.5	7.4 ± 0.4

[a] Aq = PBS 0.1X, Org = EtOH.

[b] Aq = PBS 0.1X, Org = DMAc.

[c] Recycling procedure from the Gd conjugate.

2.3 LNO@[LnL^A] and LNO@[LnL^D] characterization

For each Ln atom, a comparative set of data for both conjugation chemistry is provided. We report (a) the DLS intensity size distribution of the replicate in PBS closest to the tabulated size value in the main text, and (b) the corresponding correlogram. The normalized FTIR spectra stack (c) highlights variation in the azide stretching band around 2100 cm-1 between the parent **LNO@Si-Talys** and the resulting conjugated LNOs. Panels (d)-(f) show a first set of STEM-EDX images of **LNO@[LnL^A]**, that can be compared with a second EDX elemental map (j) of the same sample grid. Similarly, panels (g)-(i) can be compared with panel (k) for **LNO@[LnL^D]**.

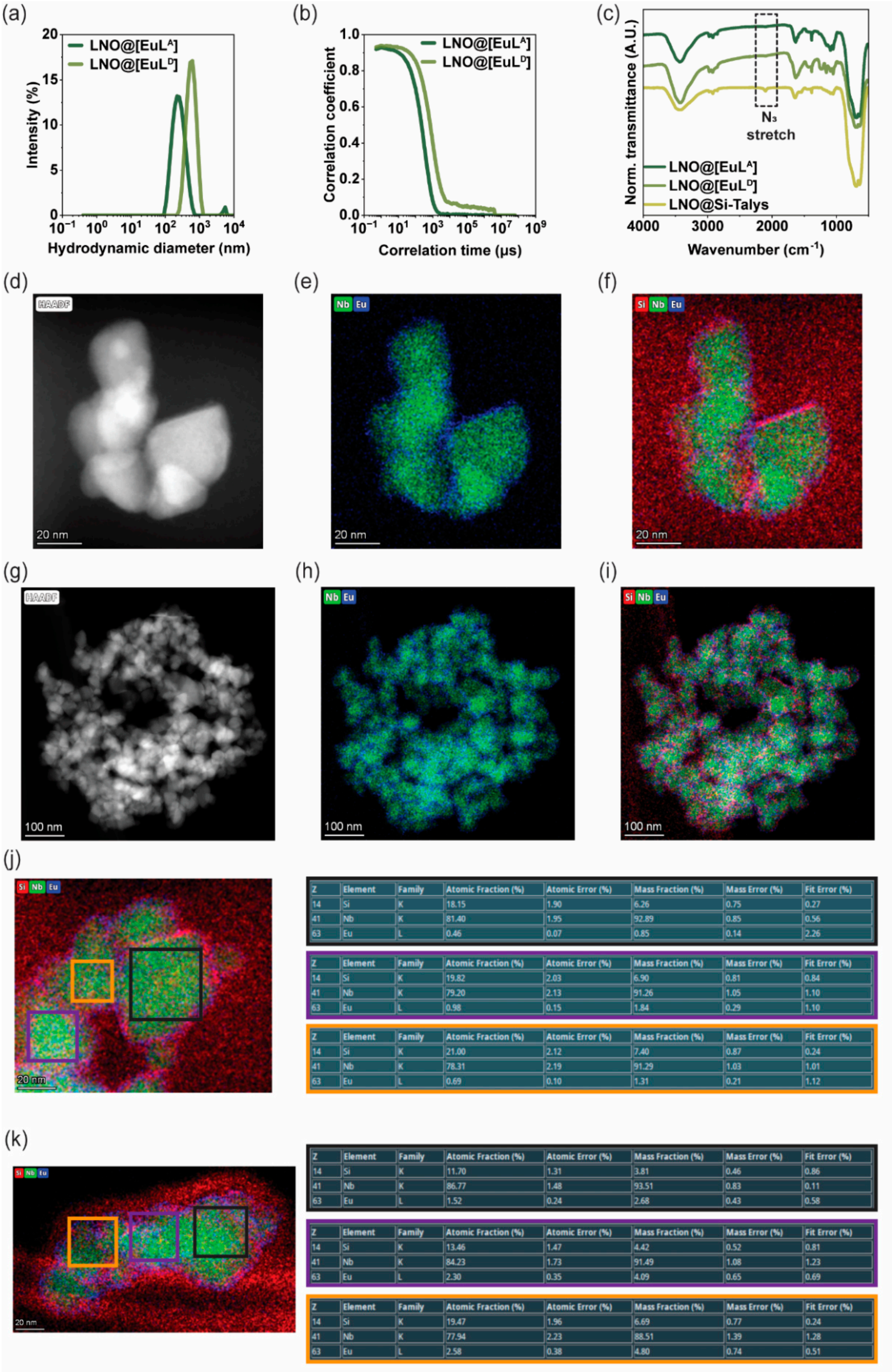

(j)

Z	Element	Family	Atomic Fraction (%)	Atomic Error (%)	Mass Fraction (%)	Mass Error (%)	Fit Error (%)
14	Si	K	18.15	1.90	6.26	0.75	0.27
41	Nb	K	81.40	1.95	92.89	0.85	0.56
63	Eu	L	0.46	0.07	0.85	0.14	2.26

Z	Element	Family	Atomic Fraction (%)	Atomic Error (%)	Mass Fraction (%)	Mass Error (%)	Fit Error (%)
14	Si	K	19.82	2.03	6.90	0.81	0.84
41	Nb	K	79.20	2.13	91.26	1.05	1.10
63	Eu	L	0.98	0.15	1.84	0.29	1.10

Z	Element	Family	Atomic Fraction (%)	Atomic Error (%)	Mass Fraction (%)	Mass Error (%)	Fit Error (%)
14	Si	K	21.00	2.12	7.40	0.87	0.24
41	Nb	K	78.31	2.19	91.29	1.03	1.01
63	Eu	L	0.69	0.10	1.31	0.21	1.12

(k)

Z	Element	Family	Atomic Fraction (%)	Atomic Error (%)	Mass Fraction (%)	Mass Error (%)	Fit Error (%)
14	Si	K	11.70	1.31	3.81	0.46	0.86
41	Nb	K	86.77	1.48	93.51	0.83	0.11
63	Eu	L	1.52	0.24	2.68	0.43	0.58

Z	Element	Family	Atomic Fraction (%)	Atomic Error (%)	Mass Fraction (%)	Mass Error (%)	Fit Error (%)
14	Si	K	13.46	1.47	4.42	0.52	0.81
41	Nb	K	84.23	1.73	91.49	1.08	1.23
63	Eu	L	2.30	0.35	4.09	0.65	0.69

Z	Element	Family	Atomic Fraction (%)	Atomic Error (%)	Mass Fraction (%)	Mass Error (%)	Fit Error (%)
14	Si	K	19.47	1.96	6.69	0.77	0.24
41	Nb	K	77.94	2.23	88.51	1.39	1.28
63	Eu	L	2.58	0.38	4.80	0.74	0.51

Figure S17. Comparative characterization of [Eu]-conjugated LNOs via CuAAC and SPAAC. DLS (a) intensity size distributions and (b) correlograms for the PBS replicate closest to the tabulated size value in the main text. Normalized stack of (c) FTIR spectra for parent **LNO@Si-Talys** and the resulting Eu conjugates. STEM (d) HAADF micrograph and EDX elemental map with (e) Nb and Eu, and (f) Si, Nb and Eu for CuAAC conjugation (thin film grid). STEM (d) HAADF micrograph and EDX elemental map with (e) Nb and Eu, and (f) Si, Nb and Eu for SPAAC conjugation (Lacey grid). (j) Estimation of the relative amount of Si, Nb and Eu in three regions of CuAAC sample (thin film grid). (k) Estimation of the relative amount of Si, Nb and Eu in three regions of SPAAC sample (Lacey grid). N.B. The images in panels (d)-(i) are the same as in **Figure 2** of the main text, and are presented here for direct visual comparison with the complementary elemental maps in panels (j)-(k).

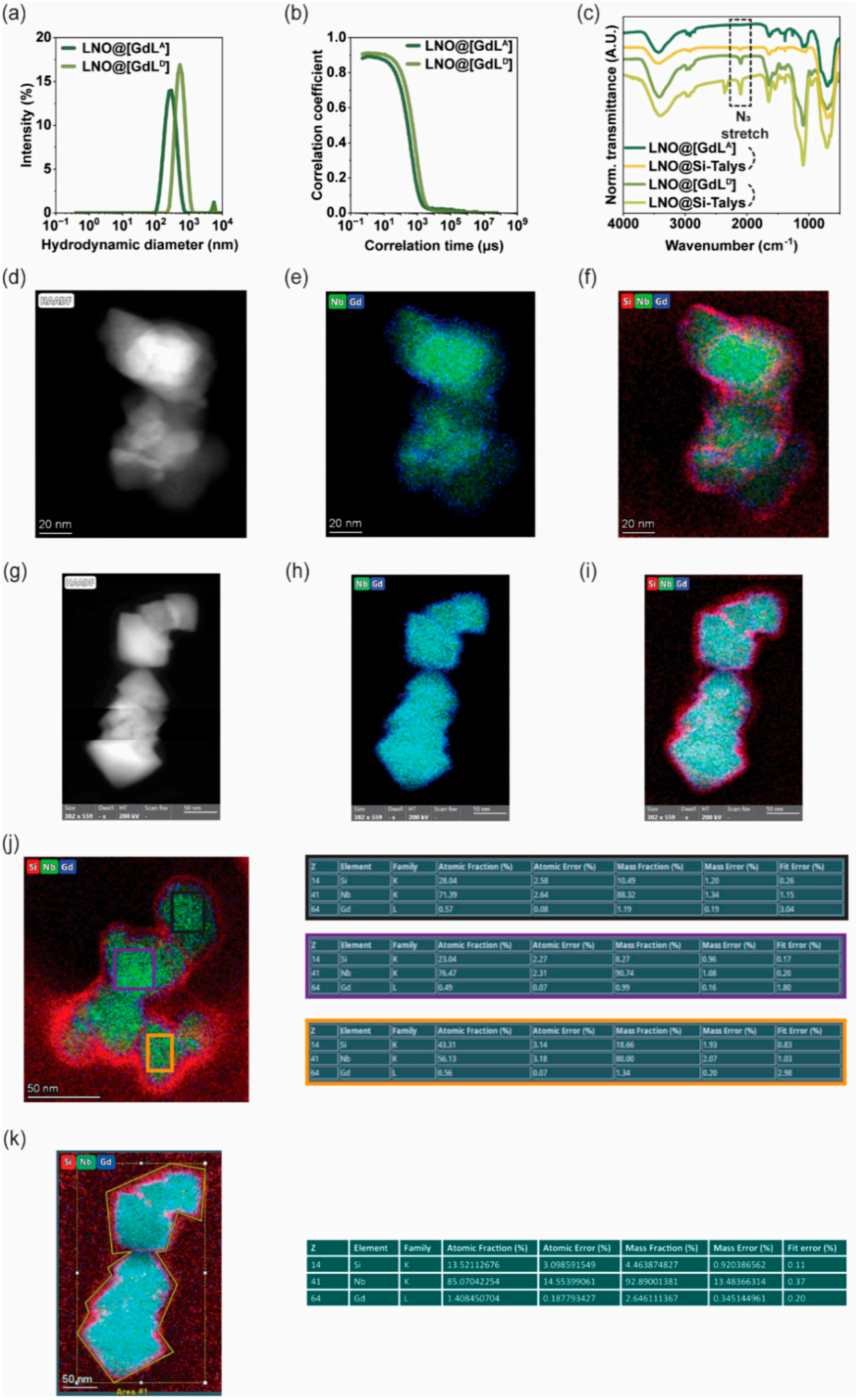

Z	Element	Family	Atomic Fraction (%)	Atomic Error (%)	Mass Fraction (%)	Mass Error (%)	Fit Error (%)
14	Si	K	28.04	2.58	10.49	1.20	0.26
41	Nb	K	71.39	2.64	88.32	1.34	1.15
64	Gd	L	0.57	0.08	1.19	0.19	3.04

Z	Element	Family	Atomic Fraction (%)	Atomic Error (%)	Mass Fraction (%)	Mass Error (%)	Fit Error (%)
14	Si	K	23.04	2.27	8.27	0.96	0.17
41	Nb	K	76.47	2.31	90.74	1.08	0.20
64	Gd	L	0.49	0.07	0.99	0.16	1.80

Z	Element	Family	Atomic Fraction (%)	Atomic Error (%)	Mass Fraction (%)	Mass Error (%)	Fit Error (%)
14	Si	K	43.31	3.14	18.66	1.93	0.83
41	Nb	K	56.13	3.18	80.00	2.07	1.03
64	Gd	L	0.56	0.07	1.34	0.20	2.98

Z	Element	Family	Atomic Fraction (%)	Atomic Error (%)	Mass Fraction (%)	Mass Error (%)	Fit error (%)
14	Si	K	13.52112676	3.098591549	4.463874827	0.920386562	0.11
41	Nb	K	85.07042254	14.55399061	92.89001381	13.48366314	0.37
64	Gd	L	1.408450704	0.187793427	2.646111367	0.345144961	0.20

Figure S18. Comparative characterization of [Gd]-conjugated LNOs via CuAAC and SPAAC. DLS (a) intensity size distributions and (b) correlograms for the PBS replicate closest to the tabulated size value in the main text. Normalized stack of (c) FTIR spectra for parent **LNO@Si-Talys** and the resulting Gd conjugates. STEM (d) HAADF micrograph and EDX elemental map with (e) Nb and Gd, and (f) Si, Nb and Gd for CuAAC conjugation (thin film grid). STEM (d) HAADF micrograph and EDX elemental map with (e) Nb and Gd, and (f) Si, Nb and Gd for SPAAC conjugation (Lacey grid). (j) Estimation of the relative amount of Si, Nb and Gd in three regions of CuAAC sample (thin film grid). (k) Estimation of the relative amount of Si, Nb and Gd in three regions of SPAAC sample (Lacey grid).

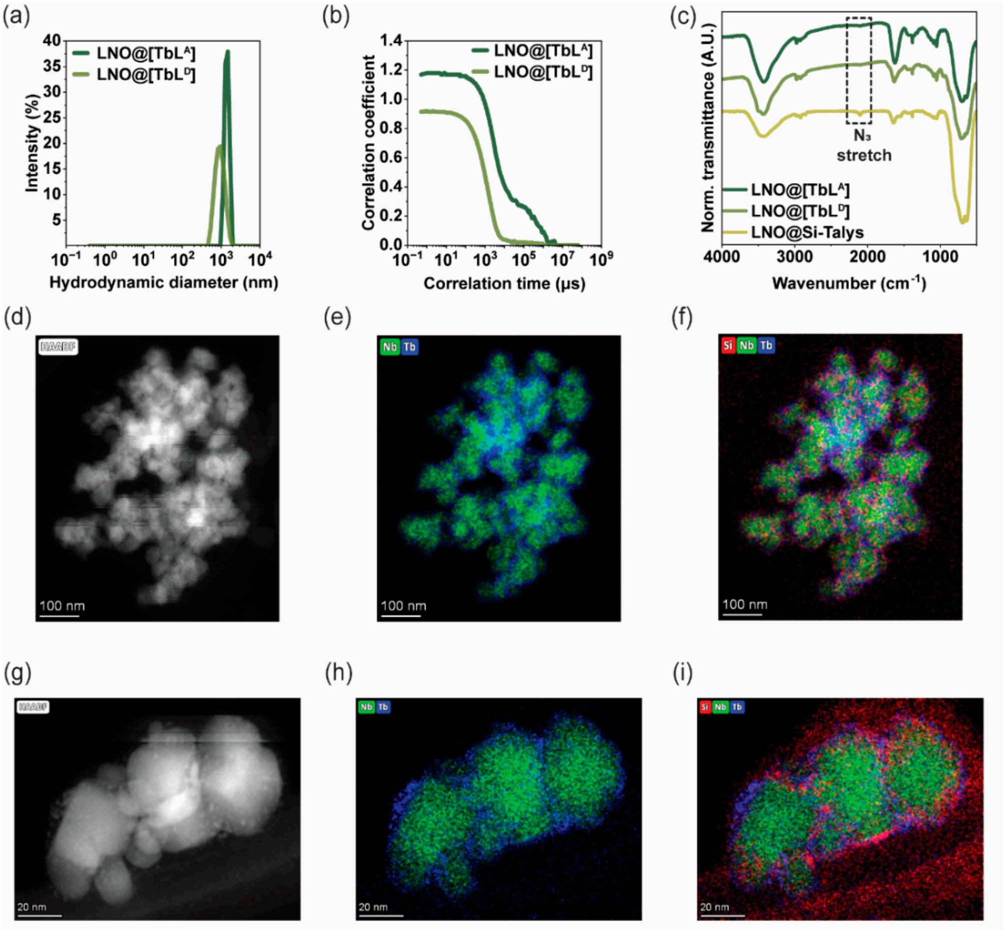

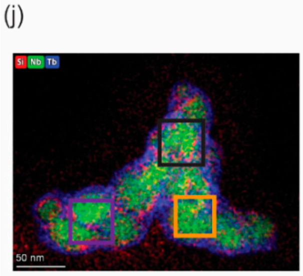

Z	Element	Family	Atomic Fraction (%)	Atomic Error (%)	Mass Fraction (%)	Mass Error (%)	Fit Error (%)
14	Si	K	5.84	0.63	1.69	0.18	1.48
41	Nb	K	82.10	2.17	78.57	2.74	0.19
65	Tb	L	12.06	1.72	19.74	2.63	0.27

Z	Element	Family	Atomic Fraction (%)	Atomic Error (%)	Mass Fraction (%)	Mass Error (%)	Fit Error (%)
14	Si	K	2.90	0.34	0.83	0.10	3.86
41	Nb	K	86.93	1.75	82.63	2.37	0.23
65	Tb	L	10.17	1.51	16.54	2.31	0.25

Z	Element	Family	Atomic Fraction (%)	Atomic Error (%)	Mass Fraction (%)	Mass Error (%)	Fit Error (%)
14	Si	K	4.62	0.51	1.33	0.14	2.15
41	Nb	K	83.60	2.06	79.51	2.67	0.20
65	Tb	L	11.78	1.70	19.16	2.58	0.38

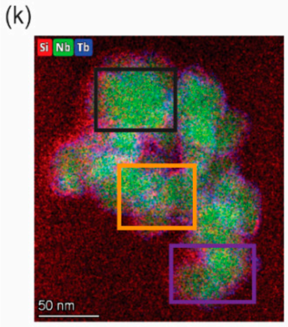

Z	Element	Family	Atomic Fraction (%)	Atomic Error (%)	Mass Fraction (%)	Mass Error (%)	Fit Error (%)
14	Si	K	17.21	1.79	5.83	0.68	0.29
41	Nb	K	80.97	1.99	90.69	1.14	0.03
65	Tb	L	1.82	0.27	3.49	0.55	0.39

Z	Element	Family	Atomic Fraction (%)	Atomic Error (%)	Mass Fraction (%)	Mass Error (%)	Fit Error (%)
14	Si	K	26.80	2.45	9.76	1.08	0.80
41	Nb	K	70.82	2.67	85.34	1.67	0.27
65	Tb	L	2.38	0.33	4.90	0.74	0.16

Z	Element	Family	Atomic Fraction (%)	Atomic Error (%)	Mass Fraction (%)	Mass Error (%)	Fit Error (%)
14	Si	K	19.91	1.98	6.83	0.77	0.74
41	Nb	K	77.34	2.27	87.82	1.47	0.14
65	Tb	L	2.75	0.40	5.35	0.82	0.15

Figure S19. Comparative characterization of [Tb]-conjugated LNOs via CuAAC and SPAAC. DLS (a) intensity size distributions and (b) correlograms for the PBS replicate closest to the tabulated size value in the main text. It is noteworthy that the LNO@[TbL^A] correlogram suggests the presence of a fluorescent impurity (y-intercept value > 1), which can explain the discrepancies with DLS and STEM data from the other CuAAC samples. Normalized stack of (c) FTIR spectra for parent **LNO@Si-Talys** and the resulting Tb conjugates. STEM (d) HAADF micrograph and EDX elemental map with (e) Nb and Tb, and (f) Si, Nb and Tb for CuAAC conjugation (Lacey grid). STEM (d) HAADF micrograph and EDX elemental map with (e) Nb and Tb, and (f) Si, Nb and Tb for SPAAC conjugation (Lacey grid). (j) Estimation of the relative amount of Si, Nb and Tb in three regions of CuAAC sample (Lacey grid). (k) Estimation of the relative amount of Si, Nb and Tb in three regions of SPAAC sample (Lacey grid).

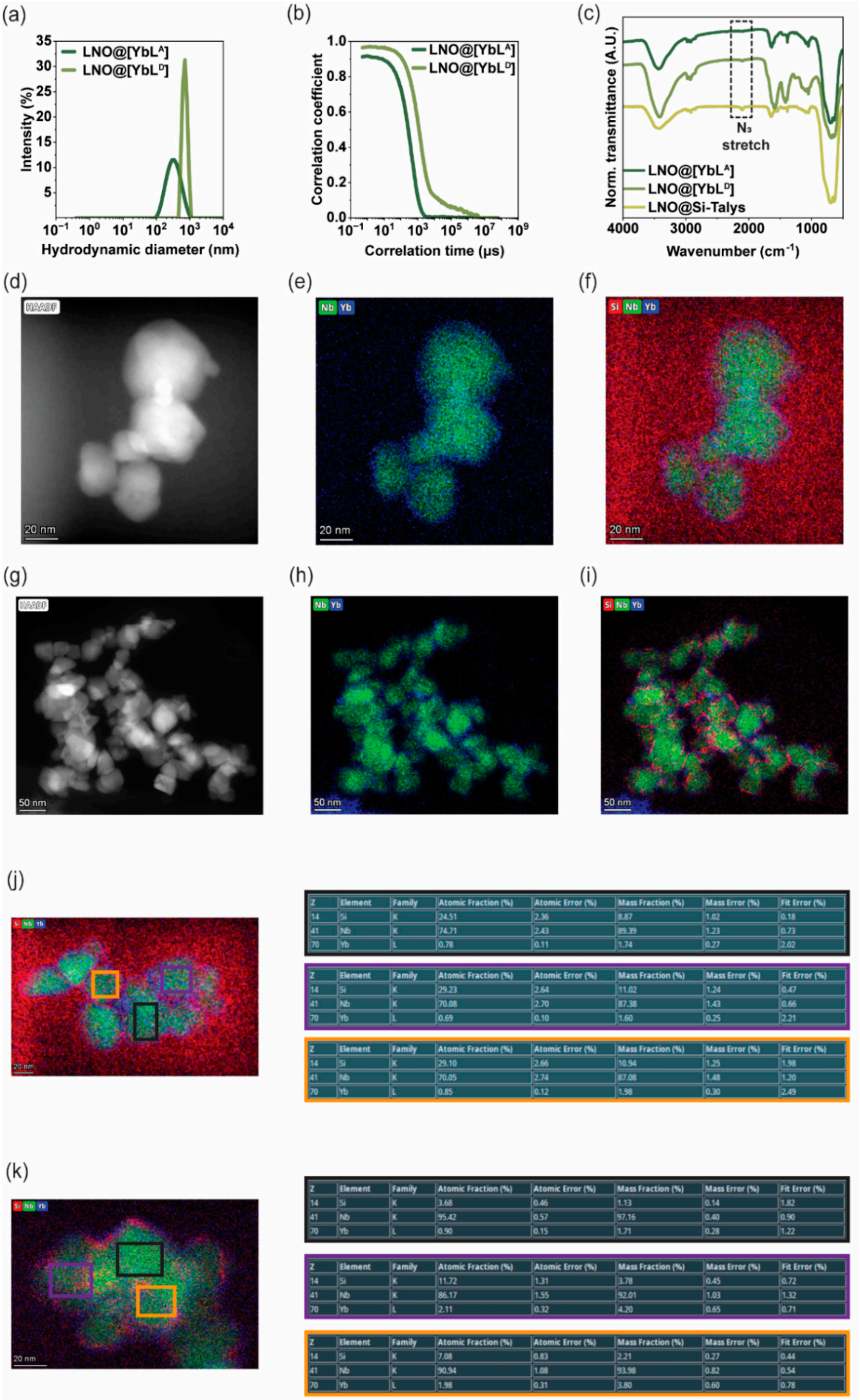

(j)

Z	Element	Family	Atomic Fraction (%)	Atomic Error (%)	Mass Fraction (%)	Mass Error (%)	Fit Error (%)
14	Si	K	24.51	2.36	8.87	1.02	0.18
41	Nb	K	74.71	2.43	89.39	1.23	0.73
70	Yb	L	0.78	0.11	1.74	0.27	2.02

Z	Element	Family	Atomic Fraction (%)	Atomic Error (%)	Mass Fraction (%)	Mass Error (%)	Fit Error (%)
14	Si	K	29.23	2.64	11.02	1.24	0.47
41	Nb	K	70.08	2.70	87.38	1.43	0.66
70	Yb	L	0.69	0.10	1.60	0.25	2.21

Z	Element	Family	Atomic Fraction (%)	Atomic Error (%)	Mass Fraction (%)	Mass Error (%)	Fit Error (%)
14	Si	K	29.10	2.66	10.94	1.25	1.98
41	Nb	K	70.05	2.74	87.08	1.48	1.20
70	Yb	L	0.85	0.12	1.98	0.30	2.49

(k)

Z	Element	Family	Atomic Fraction (%)	Atomic Error (%)	Mass Fraction (%)	Mass Error (%)	Fit Error (%)
14	Si	K	3.68	0.46	1.13	0.14	1.82
41	Nb	K	95.42	0.57	97.16	0.40	0.90
70	Yb	L	0.90	0.15	1.71	0.28	1.22

Z	Element	Family	Atomic Fraction (%)	Atomic Error (%)	Mass Fraction (%)	Mass Error (%)	Fit Error (%)
14	Si	K	11.72	1.31	3.78	0.45	0.72
41	Nb	K	86.17	1.55	92.01	1.03	1.32
70	Yb	L	2.11	0.32	4.20	0.65	0.71

Z	Element	Family	Atomic Fraction (%)	Atomic Error (%)	Mass Fraction (%)	Mass Error (%)	Fit Error (%)
14	Si	K	7.08	0.83	2.21	0.27	0.44
41	Nb	K	90.94	1.08	93.98	0.82	0.54
70	Yb	L	1.98	0.31	3.80	0.60	0.78

Figure S20. Comparative characterization of [Yb]-conjugated LNOs via CuAAC and SPAAC. DLS (a) intensity size distributions and (b) correlograms for the PBS replicate closest to the tabulated size value in the main text. Normalized stack of (c) FTIR spectra for parent **LNO@Si-Talys** and the resulting Yb conjugates. STEM (d) HAADF micrograph and EDX elemental map with (e) Nb and Yb, and (f) Si, Nb and Yb for CuAAC conjugation (thin film grid). STEM (d) HAADF micrograph and EDX elemental map with (e) Nb and Yb, and (f) Si, Nb and Yb for SPAAC conjugation (Lacey grid). (j) Estimation of the relative amount of Si, Nb and Yb in three regions of CuAAC sample (thin film grid). (k) Estimation of the relative amount of Si, Nb and Yb in three regions of SPAAC sample (Lacey grid).

3 Imaging experiments

3.1 MRI phantom imaging

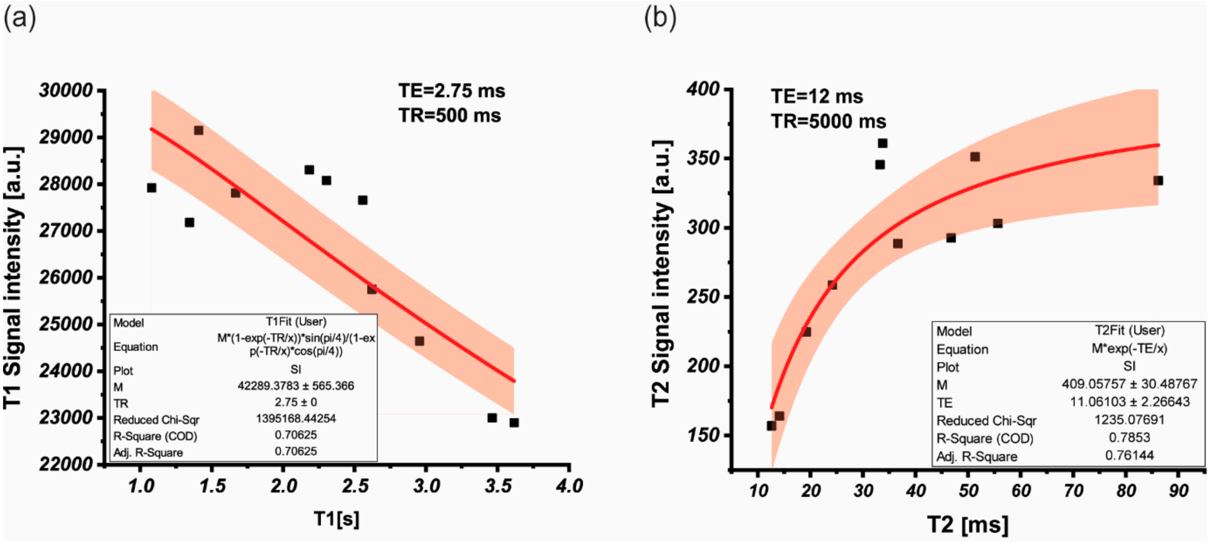

Figure S21. Detailed fitting parameters for (a) realistic monotonic signal intensity variations over T_1 range, and (b) mono-exponential signal intensity dependence over T_2 range, related to the NPs concentration.

3.2 Nonlinear optical microscopy & Ln luminescence via LNO SHG

To highlight that the colocalization of PL and SHG signals is associated with the presence of particles, we relied on a different custom-made multiphoton microscope. This system, pumped by an *Insight X3* OPO (Newport, Spectra-Physics) tuned at 790 nm is equipped with a piezo-stage for scanning the sample, a NA=1.3 oil immersion objective for excitation and a NA=0.9 objective for collection in transmission geometry. The signal is acquired by an EMCCD placed at the output of an imaging spectrometer used to spectrally separate SHG from PL. In **Figure S22**, we present an example of SHG (b) and PL (c) spatially-resolved maps overlaid to bright field images of the same sample region. For these measurements, the excitation polarization was optimized for maximum signal intensity

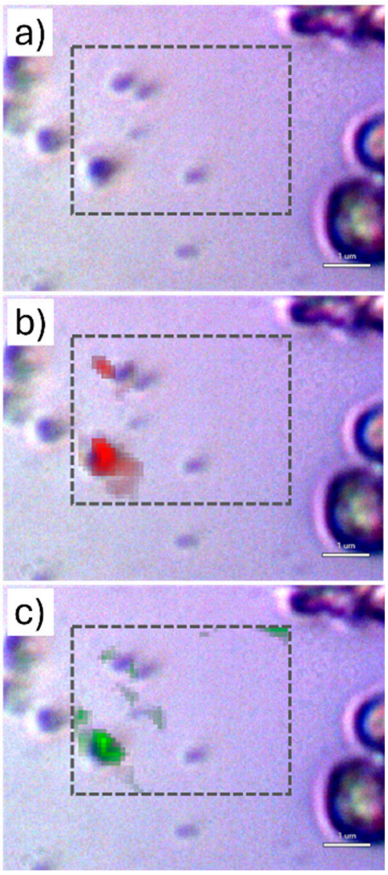

Figure S22. (a) Bright field optical image of **LNO@[EuL^{A}]** HNPs on glass substrate. (b) SHG intensity (red) and (c) PL intensity (green) maps overlaid to the bright field image. The dashed line shows the 5 µm x4 µm region of interest imaged with the scanning system.

3.3 Photophysical properties

Table S2. Lifetimes of **[LnL^A]** and **LNO@[LnL^A]** in DMSO or in H_2O (conc: 4 mM).

Entry	Compound	Solvent	λ_{ex}/nm	λ_{em}/nm	τ_1/µs	τ_2/µs
**1**	**H_3L^A**	DMSO	355	466	9.20 ± 0.89	0.69 ± 0.02
**2**		H_2O	361	436	8.16 ± 0.54	0.49 ± 0.01
**3**	**[GdL^A]**	DMSO	355	466	8.76 ± 0.08	1.11 ± 0.04
**4**		H_2O	297	411	7.56 ± 0.9	0.45 ± 0.01
**5**	**LNO@[GdL^A]**	DMSO	363	438	9.11 ± 0.06	1.29 ± 0.03
**6**		H_2O	297	393	5.01 ± 0.7	0.44 ± 0.01
**7**	**[EuL^A]**	DMSO	292	395	1229 ± 1.2	/
**8**		H_2O	286	612	755.8 ± 3.8	/
**9**	**LNO@[EuL^A]**	DMSO	292	612	922.8 ± 1.3	/
**10**		H_2O	286	613	113.5 ± 0.57	1.87 ± 0.10

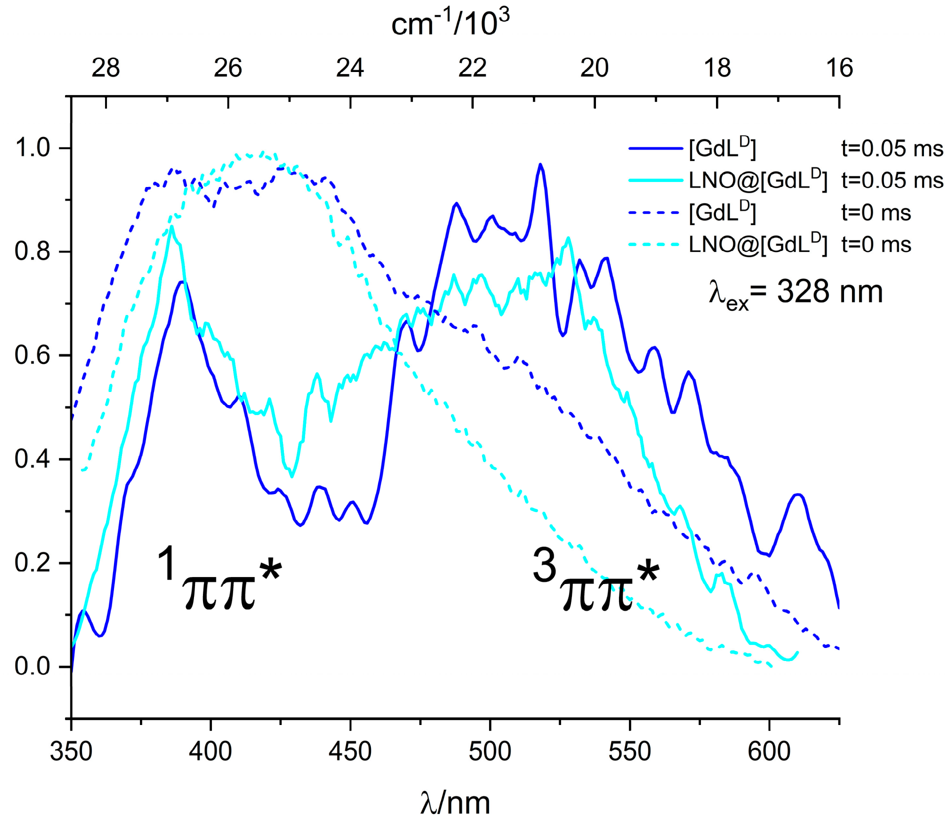

Figure S23. Normalized, corrected emission and excitation spectra of **[GdL^D]** and **LNO@[GdL^D]** recorded in the solid state at room temperature**.** Dotted lines: fluorescence spectra (t = 0 ms); solid lines: phosphorescence spectra (t = 0.05 ms).

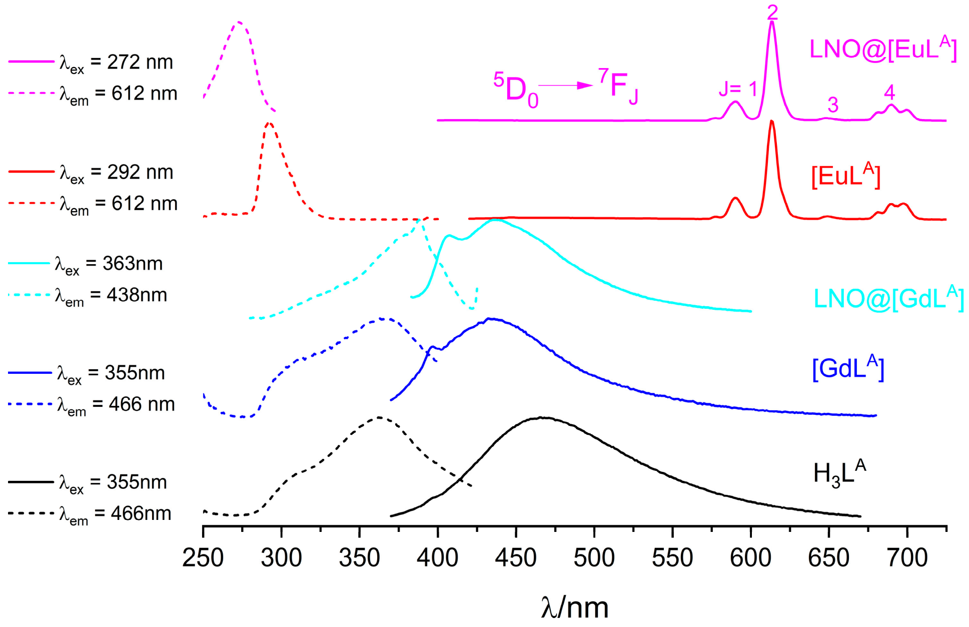

Figure S24. Normalized, corrected emission and excitation spectra of the **[LnL^A]** complexes and the corresponding nanoparticle systems **LNO@[LnL^A]** recorded in DMSO solutions (4 mM) at room temperature. Dotted lines represent excitation spectra recorded at the emission maxima, while solid lines correspond to emission spectra recorded at the excitation maxima.

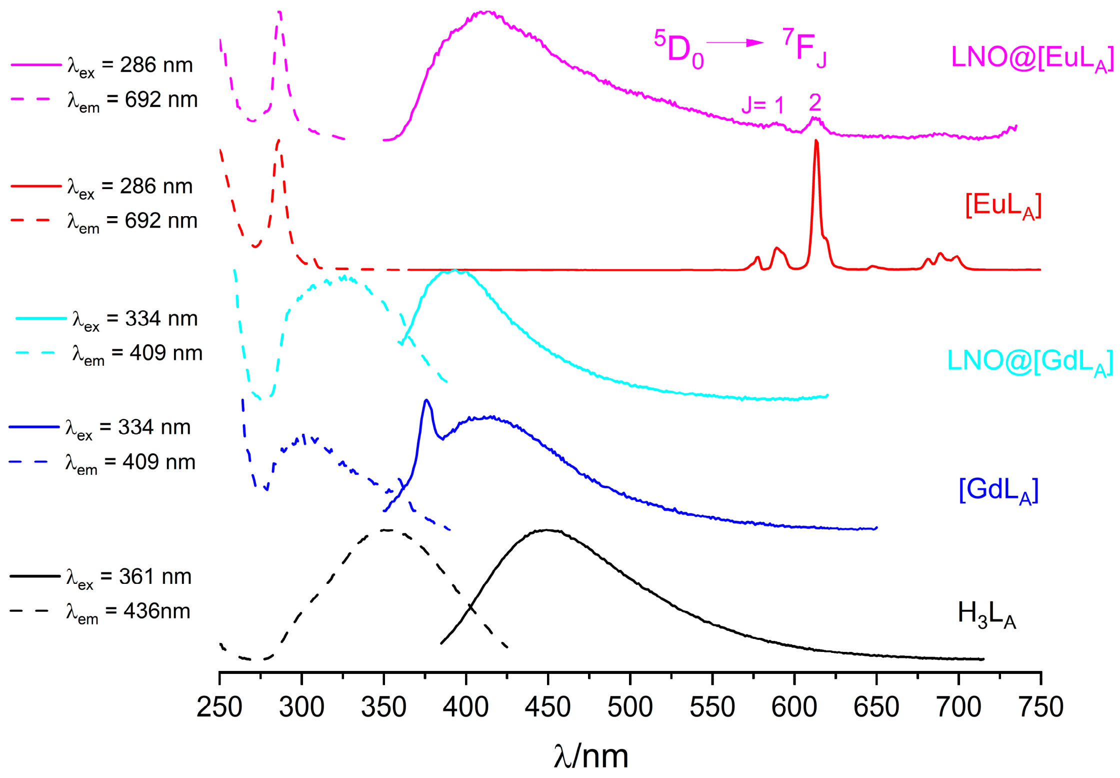

Figure S25. Normalized, corrected emission and excitation spectra of the **[LnL^A]** complexes and the corresponding nanoparticle systems **LNO@[LnL^A]** recorded in aqueous solutions (4 mM) at room temperature. Dotted lines represent excitation spectra recorded at the emission maxima, while solid lines correspond to emission spectra recorded at the excitation maxima.

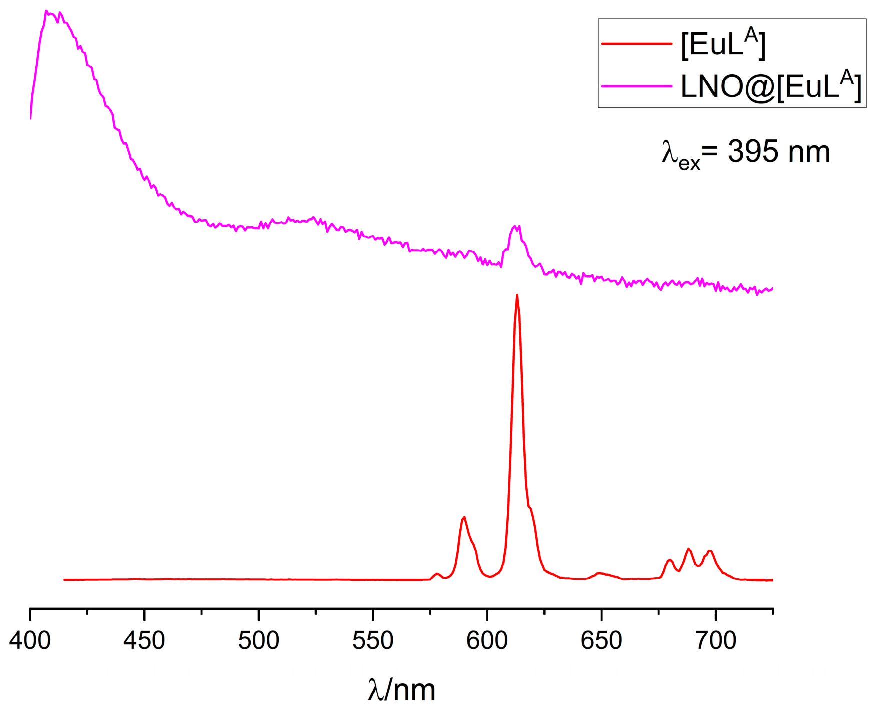

Figure S26. Normalized, corrected emission spectra of the **[EuL^A]** (red) and **LNO@[EuL^A]** (pink) recorded in the solid state at room temperature**,** upon excitation at 395 nm to populate the $^5L_6$ excited level of trivalent europium from the ground $^7F_0$ state. Comparing the pink traces in Figure S11 and Figure S9 highlights the significantly lower energy transfer to Eu when using direct excitation compared to ligand sensitization.

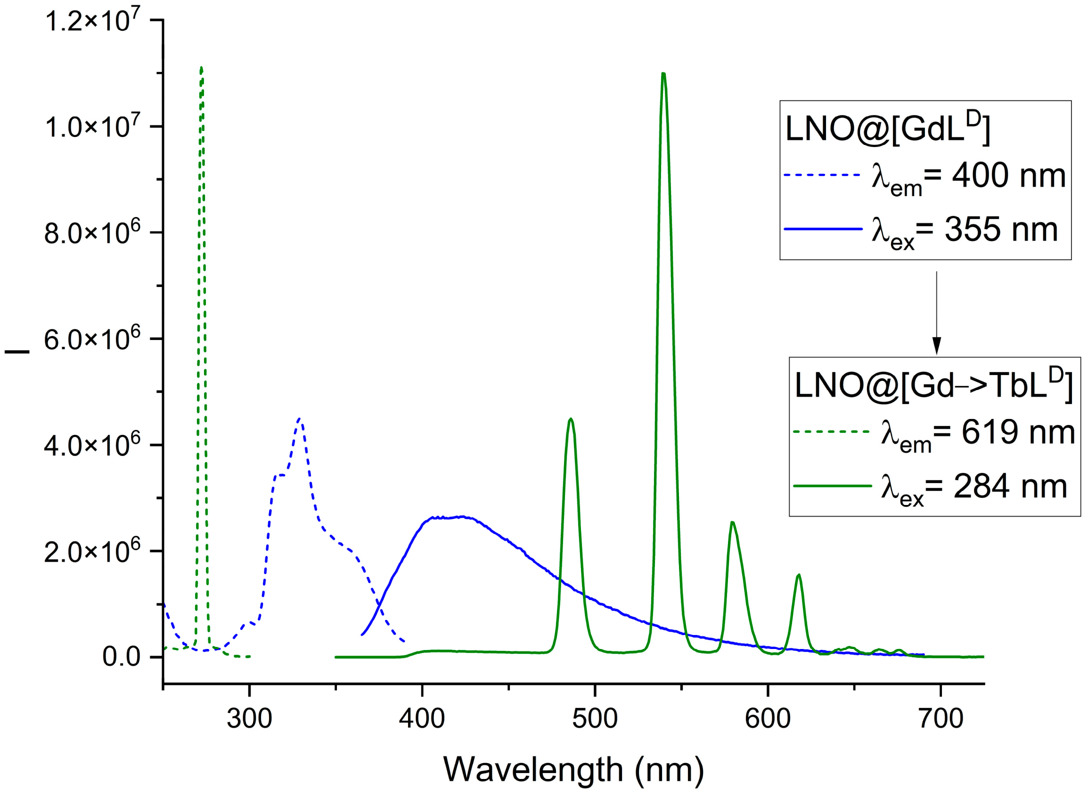

Figure S27. Emission and excitation corrected spectra of the **LNO@[GdL^{D}]** and **LNO@[Gd→TbL^{D}]** in H_2O at room temperature.

[1] A. Gheata, A. Spada, M. Wittwer, A. Dhouib, E. Molina, Y. Mugnier, S. Gerber-Lemaire, "Modulating the Surface Properties of Lithium Niobate Nanoparticles by Multifunctional Coatings Using Water-in-Oil Microemulsions" *Nanomaterials* **2023**, *13*, 522.
